# Supplementary material for: Differential circulating miRNA profiles identified miR-423-5p, miR-93-5p, and miR-4532 as potential biomarkers for cholangiocarcinoma diagnosis
Source: PeerJ. 2024 Dec 10;12:e18367. doi: 10.7717/peerj.18367 (PMC11639864; doi:10.7717/peerj.18367)
Supplement: Supplemental Information 2 — The down-regulated miRNA profile of CCA against HC was predicted by reactome. [file peerj-12-18367-s002.pdf]

reactome

Pathway Analysis Report

This report contains the pathway analysis results for the submitted sample ''. Analysis was performed against Reactome version 87 on 15/03/2024. The web link to these results is:

https://reactome.org/PathwayBrowser/#/ANALYSIS=MjAyNDAzMTQwNDMwMDVfMTU2Nzc%3D

Please keep in mind that analysis results are temporarily stored on our server. The storage period depends on usage of the service but is at least 7 days. As a result, please note that this URL is only valid for a limited time period and it might have expired.

Table of Contents

1. Introduction
2. Properties
3. Genome-wide overview
4. Most significant pathways
5. Pathways details
6. Identifiers found
7. Identifiers not found

1. Introduction

Reactome is a curated database of pathways and reactions in human biology. Reactions can be considered as pathway 'steps'. Reactome defines a 'reaction' as any event in biology that changes the state of a biological molecule. Binding, activation, translocation, degradation and classical biochemical events involving a catalyst are all reactions. Information in the database is authored by expert biologists, entered and maintained by Reactome's team of curators and editorial staff. Reactome content frequently cross-references other resources e.g. NCBI, Ensembl, UniProt, KEGG (Gene and Compound), ChEBI, PubMed and GO. Orthologous reactions inferred from annotation for Homo sapiens are available for 14 non-human species including mouse, rat, chicken, puffer fish, worm, fly and yeast. Pathways are represented by simple diagrams following an SBGN-like format.

Reactome's annotated data describe reactions possible if all annotated proteins and small molecules were present and active simultaneously in a cell. By overlaying an experimental dataset on these annotations, a user can perform a pathway over-representation analysis. By overlaying quantitative expression data or time series, a user can visualize the extent of change in affected pathways and its progression. A binomial test is used to calculate the probability shown for each result, and the p-values are corrected for the multiple testing (Benjamini–Hochberg procedure) that arises from evaluating the submitted list of identifiers against every pathway.

To learn more about our Pathway Analysis, please have a look at our relevant publications:

Fabregat A, Sidiropoulos K, Garapati P, Gillespie M, Hausmann K, Haw R, … D'Eustachio P (2016). The reactome pathway knowledgebase. Nucleic Acids Research, 44(D1), D481–D487. https://doi.org/10.1093/nar/gkv1351.

Fabregat A, Sidiropoulos K, Viteri G, Forner O, Marin-Garcia P, Arnau V, … Hermjakob H (2017). Reactome pathway analysis: a high-performance in-memory approach. BMC Bioinformatics, 18.

2. Properties

- This is an **overrepresentation** analysis: A statistical (hypergeometric distribution) test that determines whether certain Reactome pathways are over-represented (enriched) in the submitted data. It answers the question ‘Does my list contain more proteins for pathway X than would be expected by chance?’ This test produces a probability score, which is corrected for false discovery rate using the Benjamani-Hochberg method.
- 10 out of 13 identifiers in the sample were found in Reactome, where 277 pathways were hit by at least one of them.
- All non-human identifiers have been converted to their human equivalent.
- IntAct interactors were included to increase the analysis background. This greatly increases the size of Reactome pathways, which maximises the chances of matching your submitted identifiers to the expanded pathway, but will include interactors that have not undergone manual curation by Reactome and may include interactors that have no biological significance, or unexplained relevance.
- This report is filtered to show only results for species 'Homo sapiens' and resource 'UniProt'.
- The unique ID for this analysis (token) is MjAyNDAzMTQwNDMwMDVfMTU2Nzc%3D. This ID is valid for at least 7 days in Reactome's server. Use it to access Reactome services with your data.

3. Genome-wide overview

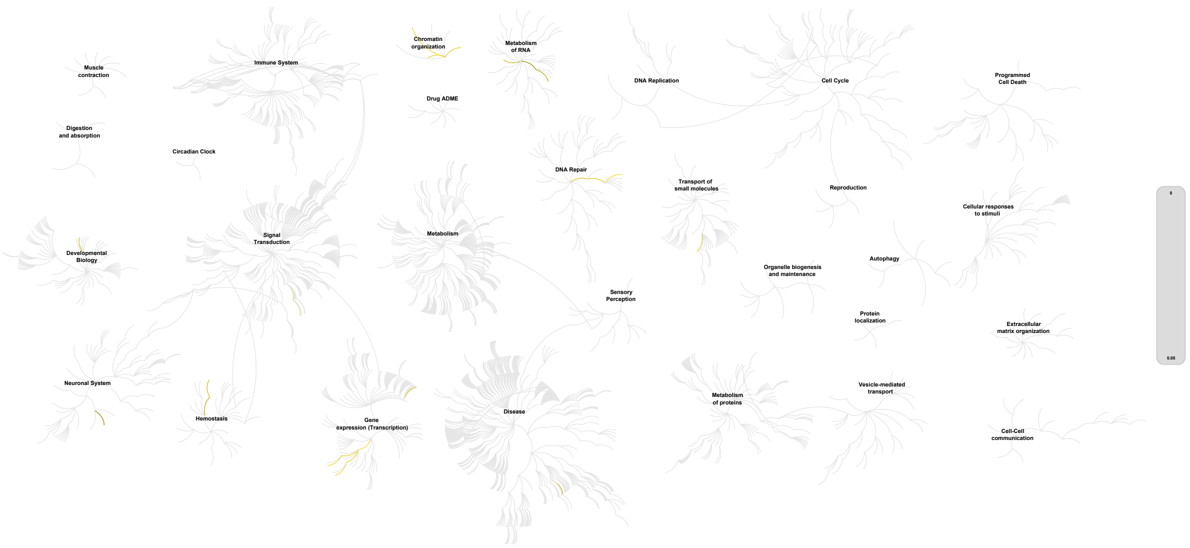

This figure shows a genome-wide overview of the results of your pathway analysis. Reactome pathways are arranged in a hierarchy. The center of each of the circular "bursts" is the root of one top-level pathway, for example "DNA Repair". Each step away from the center represents the next level lower in the pathway hierarchy. The color code denotes over-representation of that pathway in your input dataset. Light grey signifies pathways which are not significantly over-represented.

4. Most significant pathways

The following table shows the 25 most relevant pathways sorted by p-value.

Pathway name	Entities				Reactions	
	found	ratio	p-value	FDR*	found	ratio
Axonal growth inhibition (RHOA activation)	2 / 35	0.002	8.31e-04	0.116	5 / 6	4.08e-04
p75NTR regulates axonogenesis	2 / 35	0.002	8.31e-04	0.116	5 / 8	5.43e-04
Epigenetic regulation of gene expression	6 / 1,097	0.058	0.002	0.139	10 / 64	0.004
Termination of translesion DNA synthesis	2 / 62	0.003	0.003	0.139	4 / 9	6.11e-04
NoRC negatively regulates rRNA expression	3 / 239	0.013	0.003	0.139	3 / 7	4.75e-04
HDACs deacetylate histones	2 / 70	0.004	0.003	0.139	2 / 5	3.40e-04
B-WICH complex positively regulates rRNA expression	3 / 255	0.013	0.003	0.139	3 / 3	2.04e-04
Negative epigenetic regulation of rRNA expression	3 / 334	0.018	0.007	0.186	3 / 12	8.15e-04
Positive epigenetic regulation of rRNA expression	3 / 344	0.018	0.008	0.186	3 / 7	4.75e-04
Chromatin organization	4 / 674	0.035	0.008	0.186	16 / 85	0.006
Chromatin modifying enzymes	4 / 674	0.035	0.008	0.186	16 / 85	0.006
TFAP2 (AP-2) family regulates transcription of other transcription factors	1 / 7	3.67e-04	0.008	0.186	1 / 2	1.36e-04
RMTs methylate histone arginines	3 / 358	0.019	0.009	0.186	5 / 22	0.001
Inositol transporters	1 / 9	4.72e-04	0.011	0.216	1 / 3	2.04e-04
Translesion synthesis by Y family DNA polymerases bypasses lesions on DNA template	2 / 143	0.007	0.013	0.231	4 / 28	0.002
DNA Damage Bypass	2 / 162	0.008	0.016	0.268	4 / 34	0.002
Inhibition of Host mRNA Processing and RNA Silencing	1 / 15	7.87e-04	0.018	0.268	1 / 2	1.36e-04
Z-decay: degradation of maternal mRNAs by zygotically expressed factors	1 / 16	8.39e-04	0.019	0.268	2 / 4	2.72e-04
cGMP effects	1 / 16	8.39e-04	0.019	0.268	1 / 4	2.72e-04
Insulin-like Growth Factor-2 mRNA Binding Proteins (IGF2BPs/IMPs/VICKZs) bind RNA	1 / 17	8.91e-04	0.02	0.284	2 / 3	2.04e-04
Nitric oxide stimulates guanylate cyclase	1 / 23	0.001	0.027	0.309	1 / 7	4.75e-04
Processing of Intronless Pre-mRNAs	1 / 26	0.001	0.031	0.309	2 / 5	3.40e-04

Pathway name	Entities				Reactions	
	found	ratio	p-value	FDR*	found	ratio
Receptor-type tyrosine-protein phosphatases	1 / 37	0.002	0.044	0.309	1 / 6	4.08e-04
Processing of Capped Intronless Pre-mRNA	1 / 38	0.002	0.045	0.309	2 / 10	6.79e-04
Transcriptional activity of SMAD2/SMAD3:SMAD4 heterotrimer	3 / 731	0.038	0.056	0.309	4 / 51	0.003

* False Discovery Rate

5. Pathways details

For every pathway of the most significant pathways, we present its diagram, as well as a short summary, its bibliography and the list of inputs found in it.

1. Axonal growth inhibition (RHOA activation) (R-HSA-193634)

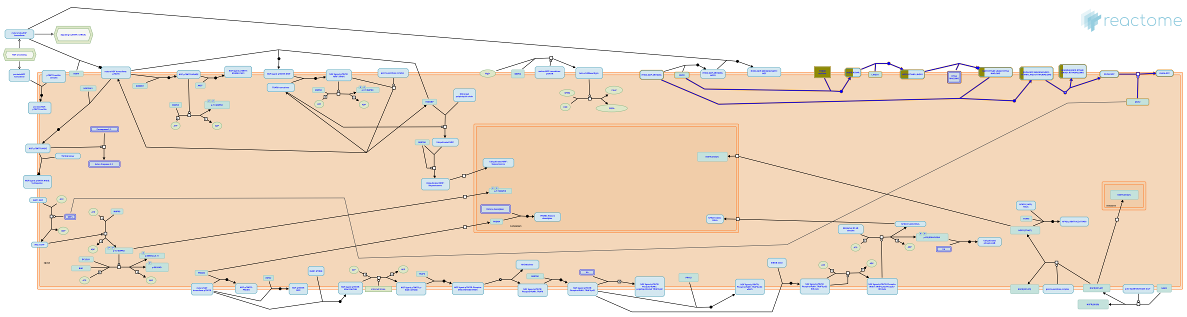

p75NTR can also form a receptor complex with the Nogo receptor (NgR). Such complexes mediates axonal outgrowth inhibitory signals of MDGIs (myelin-derived growth-inhibitors), such as Nogo66, myelin-associated glycoprotein (MAG), and oligodendrocyte myelin glycoprotein (OMGP).

References

Filbin MT (2003). Myelin-associated inhibitors of axonal regeneration in the adult mammalian CNS. Nat Rev Neurosci, 4, 703-13.

Edit history

Date	Action	Author
2006-10-10	Authored	Annibali D, Nasi S
2007-02-23	Created	Jassal B
2008-05-20	Edited	Jassal B
2008-05-20	Reviewed	Friedman WJ
2008-05-28	Reviewed	Chao MV
2023-11-16	Modified	Wright A

1 submitted entities found in this pathway, mapping to 1 Reactome entities

Input	UniProt Id
RTN4R	Q9BZR6

Interactors found in this pathway (1)

Input	UniProt Id	Interacts with	Input	UniProt Id	Interacts with
RTN4R	Q99M75	Q96FE5			

2. p75NTR regulates axonogenesis (R-HSA-193697)

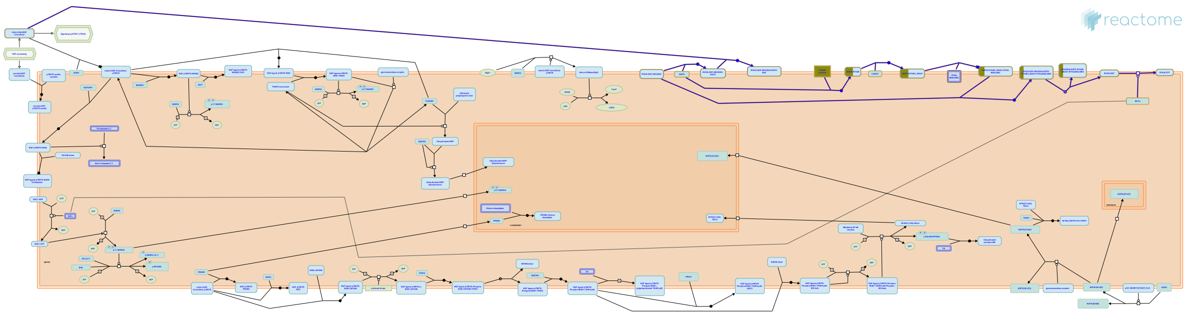

p75NTR modulates axonal growth by regulating the activity of small GTPases like RHOA and RHOB, that control the state of actin polymerization. The best studied is RHOA. In its active, GTP-bound form, RHOA rigidifies the actin cytoskeleton, thereby inhibiting axonal elongation and causing growth cone collapse. Depending on the ligand that binds to it, p75NTR can either promote or inhibit axonal growth, Neurotrophin binding leads to inhibition of RHOA activity and axonal growth. Axonal growth inhibition is caused by myelin molecules named MDGIs (myelin-derived growth inhibitors), such as NOGO, MAG, OMGP. MDGIs bind to a complex made up of p75NTR and the NOGO receptor, causing RHOA activation and axonal growth inhibition.

References

Li HY & Zhou XF (2007). Roles of glial p75NTR in axonal regeneration. J Neurosci Res, 85, 1601-5.

Edit history

Date	Action	Author
2006-10-10	Authored	Annibali D, Nasi S
2007-02-23	Created	Jassal B
2008-05-20	Edited	Jassal B
2008-05-20	Reviewed	Friedman WJ
2008-05-28	Reviewed	Chao MV
2023-11-16	Modified	Wright A

1 submitted entities found in this pathway, mapping to 1 Reactome entities

Input	UniProt Id
RTN4R	Q9BZR6

Interactors found in this pathway (1)

Input	UniProt Id	Interacts with	Input	UniProt Id	Interacts with
RTN4R	Q99M75	Q96FE5			

3. Epigenetic regulation of gene expression (R-HSA-212165)

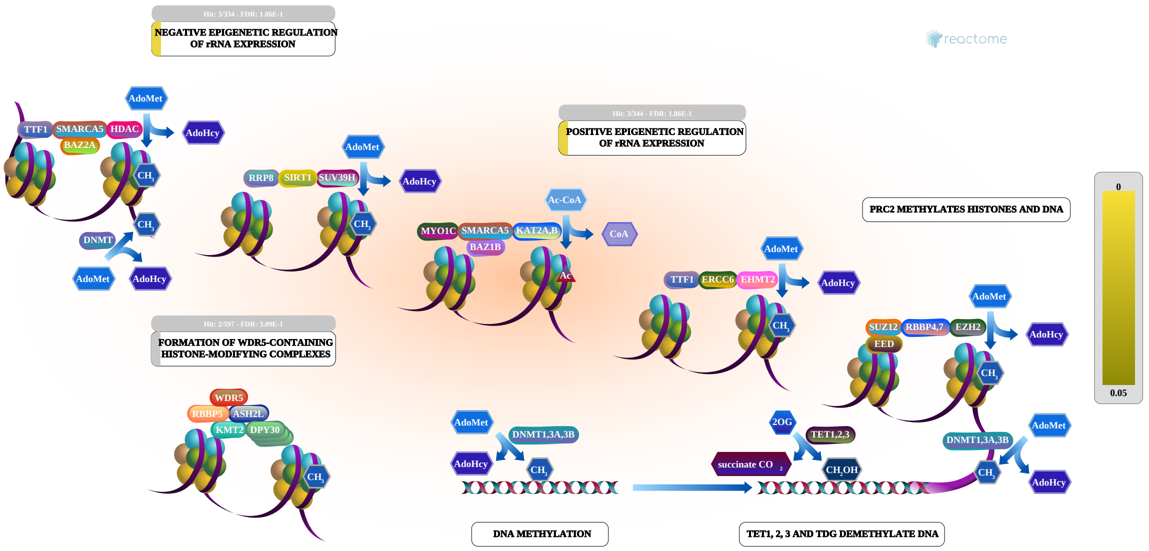

Cellular compartments: nucleoplasm.

Epigenetic processes regulate gene expression by modulating the frequency, rate, or extent of gene expression in a mitotically or meiotically heritable way that does not entail a change in the DNA sequence. Originally the definition applied only to heritability across generations but later also encompassed the heritable changes that occur during cellular differentiation within one organism.

Molecular analysis shows epigenetic changes comprise covalent modifications, such as methylation and acetylation, to DNA and histones. RNA interference has been implicated in the initiation of some epigenetic changes, for example transcriptional silencing of transposons. Proteins which bind to the modified DNA and histones are then responsible for repressing transcription and for maintaining the epigenetic modifications during cell division.

During differentiation, patterns of gene expression are established by polycomb complexes PRC1 and PRC2. PRC2 methylates histones and DNA to produce the initial marks of repression: trimethylated lysine-27 on histone H3 (H3K27me3) and 5-methylcytosine in DNA. PRC2, through its component EZH2 or, in some complexes, EZH1 trimethylates lysine-27 of histone H3. The H3K27me3 produced by PRC2 is bound by the Polycomb subunit of PRC1. PRC1 ubiquitinates histone H2A and maintains repression.

PRC2 and other epigenetic systems modulate gene expression through DNA methyation, the transfer of a methyl group from S-adenosylmethionine to the 5 position of cytosine in DNA by a family of DNA methyltransferases (DNMTs): DNMT1, DNMT3A, and DNMT3B.

In the reverse process TET1,2,3 and TDG demethylate DNA through the oxidation of the methyl group of 5-methylcytosine by TET enzymes and the excision of the oxidized product (5-formylcytosine or 5-carboxylcytosine) by TDG.

Ribosomal RNA (rRNA) genes are activated and deactivated according to the metabolic requirements of the cell. Positive epigenetic regulation of rRNA expression occurs through chromatin modifications produced by activators such as ERCC6 (CSB), the B-WICH complex, and histone acetylases such as KAT2B (PCAF). Negative epigenetic regulation of rRNA expression occurs through chromatin modifications produced by repressors such as the eNoSC complex, SIRT1, and the NoRC complex.

WDR5 is a component of six histone methyltransferases and three histone acetyltransferases involved in epigenetic regulation of gene expression (reviewed in Guarnaccia and Tansey 2018).

References

Tansey WP & Guarnaccia AD (2018). Moonlighting with WDR5: A Cellular Multitasker. J Clin Med, 7 .

Bird A & Jaenisch R (2003). Epigenetic regulation of gene expression: how the genome integrates intrinsic and environmental signals. Nat. Genet., 33, 245-54.

Edit history

Date	Action	Author
2008-02-09	Edited	May B, Gopinathrao G
2008-02-09	Authored	May B, Gopinathrao G
2008-02-09	Created	May B, Gopinathrao G
2009-07-06	Edited	May B
2014-02-26	Reviewed	Matthews L
2023-11-17	Modified	Wright A

2 submitted entities found in this pathway, mapping to 3 Reactome entities

Input	UniProt Id	Input	UniProt Id
SAP30	O75446, Q9HAJ7	SMARCA5	O60264

Interactors found in this pathway (5)

Input	UniProt Id	Interacts with	Input	UniProt Id	Interacts with
CEBPZ	Q03701	P49841	PDE9A	O76083-2	P61964
SAP30	O75446	Q92769, Q01658, Q13547	SMARCA5	O60264	Q9UIG0
YBX1	P67809	Q9NR30			

4. Termination of translesion DNA synthesis (R-HSA-5656169)

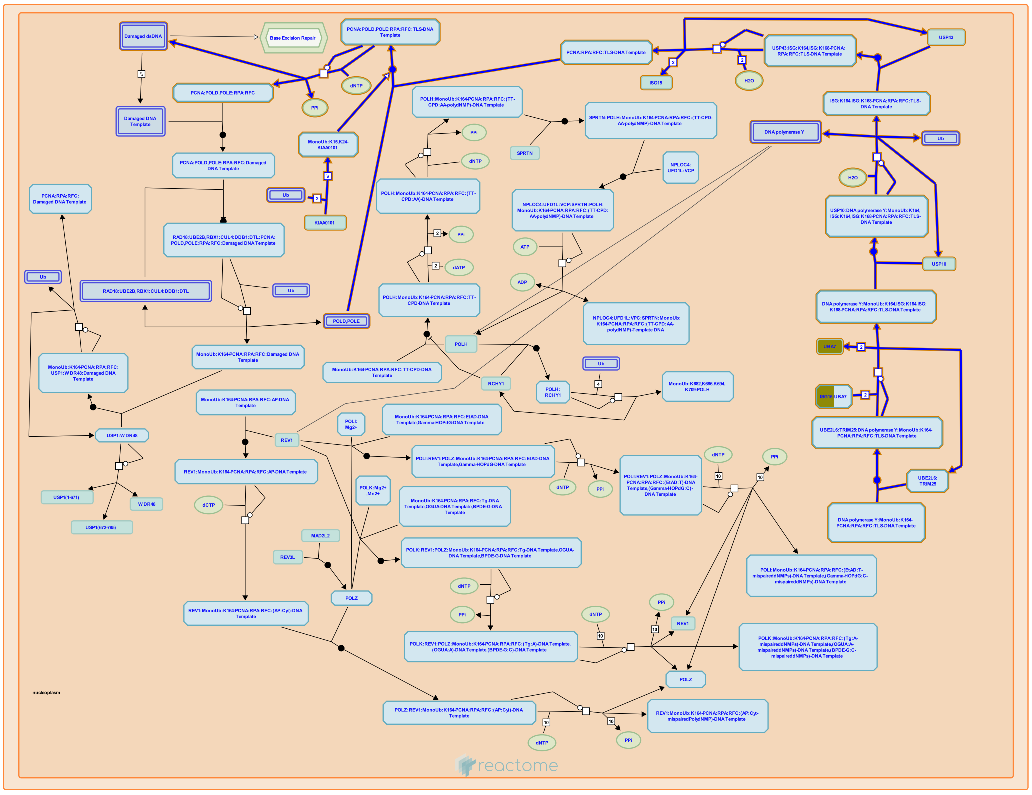

Cellular compartments: nucleoplasm.

The initiation and extent of translesion DNA synthesis (TLS) has to be tightly controlled in order to limit TLS-induced mutagenesis, caused by the low fidelity of TLS-participating DNA polymerases. Since monoubiquitination of PCNA at lysine residue K164 is a prerequisite for the assembly of TLS complexes on damaged DNA templates, PCNA deubiquitination is a key step in TLS termination that allows DNA polymerase switching from Y family DNA polymerases involved in TLS to replicative DNA polymerases delta and epsilon (Povlsen et al. 2012, Park et al. 2014).

References

Nielsen ML, Poulsen SL, Sylvestersen KB, Beli P, Choudhary C, Povlsen LK, ... Wagner SA (2012). Systems-wide analysis of ubiquitylation dynamics reveals a key role for PAF15 ubiquitylation in DNA-damage bypass. Nat. Cell Biol., 14, 1089-98.

Lee SW, Seol JH, Yang SW, Yu KR, Park JM, Ka SH, ... Chung CH (2014). Modification of PCNA by ISG15 plays a crucial role in termination of error-prone translesion DNA synthesis. Mol. Cell, 54, 626-38.

Edit history

Date	Action	Author
2014-12-11	Edited	Orlic-Milacic M
2014-12-11	Authored	Orlic-Milacic M
2014-12-11	Created	Orlic-Milacic M
2015-01-07	Reviewed	Borowiec JA
2023-11-28	Modified	Wright A

1 submitted entities found in this pathway, mapping to 1 Reactome entities

Input	UniProt Id
UBA7	P41226

Interactors found in this pathway (2)

Input	UniProt Id	Interacts with	Input	UniProt Id	Interacts with
UBA7	P41226	P05161	YBX1	P67809	Q14694

5. NoRC negatively regulates rRNA expression (R-HSA-427413)

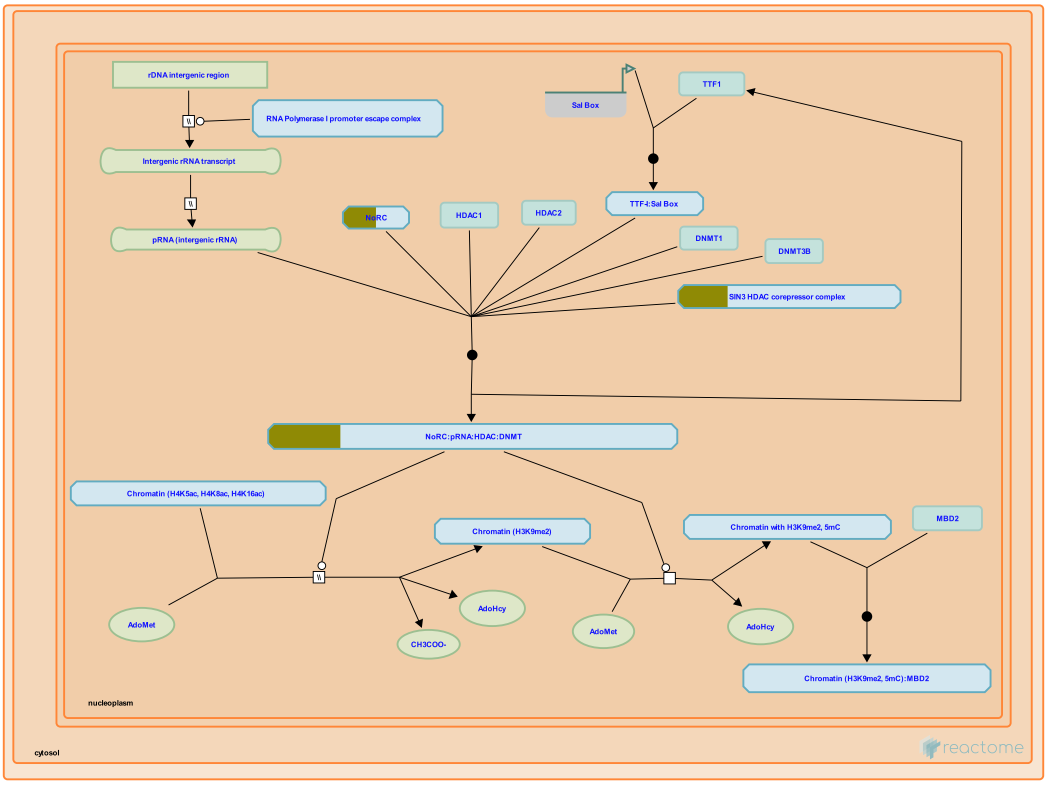

Cellular compartments: nucleoplasm.

The Nucleolar Remodeling Complex (NoRC) comprising TIP5 (BAZ2A) and the chromatin remodeller SNF2H (SMARCA5) silences rRNA gene (reviewed in Santoro and Grummt 2001, Grummt 2007, Preuss and Pikaard 2007, Birch and Zommerdijk 2008, McStay and Grummt 2008, Grummt and Langst 2013). The TAM domain of TIP5 (BAZ2A) binds promoter-associated RNA (pRNA) transcribed from the intergenic spacer region of rDNA. The pRNA bound by TIP5 is required to direct the complex to the main promoter of the rRNA gene possibly by triple helix formation between pRNA and the rDNA. The PHD domain of TIP5 binds histone H4 acetylated at lysine-16. Transcription Termination Factor-I (TTF-I) binds to a promoter-proximal terminator (T0 site) in the rDNA and interacts with the TIP5 subunit of NoRC. NoRC also interacts with the SIN3-HDAC complex, HDAC1, HDAC2, DNMT1, and DNMT3B. DNMT3B interacts with a triple helix formed by pRNA and the rDNA. HDAC1, DNMT1, and DNMT3B have been shown to be required for proper DNA methylation of silenced rRNA gene copies, although the catalytic activity of DNMT3B was not required.

References

Grummt I (2007). Different epigenetic layers engage in complex crosstalk to define the epigenetic state of mammalian rRNA genes. Hum Mol Genet, 16, R21-7.

Pikaard CS & Preuss S (2007). rRNA gene silencing and nucleolar dominance: insights into a chromosome-scale epigenetic on/off switch. Biochim Biophys Acta, 1769, 383-92.

Santoro R & Grummt I (2001). Molecular mechanisms mediating methylation-dependent silencing of ribosomal gene transcription. Mol Cell, 8, 719-25.

Grummt I & McStay B (2008). The epigenetics of rRNA genes: from molecular to chromosome biology. Annu Rev Cell Dev Biol, 24, 131-57.

Längst G & Grummt I (2013). Epigenetic control of RNA polymerase I transcription in mammalian cells. Biochim. Biophys. Acta, 1829, 393-404.

Edit history

Date	Action	Author
2009-06-19	Authored	May B
2009-06-20	Created	May B
2010-04-06	Edited	May B
2014-02-18	Reviewed	Shiao YH
2023-11-16	Modified	Wright A

2 submitted entities found in this pathway, mapping to 3 Reactome entities

Input	UniProt Id	Input	UniProt Id
SAP30	O75446, Q9HAJ7	SMARCA5	O60264

Interactors found in this pathway (1)

Input	UniProt Id	Interacts with	Input	UniProt Id	Interacts with
SAP30	O75446	Q92769, Q13547			

6. HDACs deacetylate histones (R-HSA-3214815)

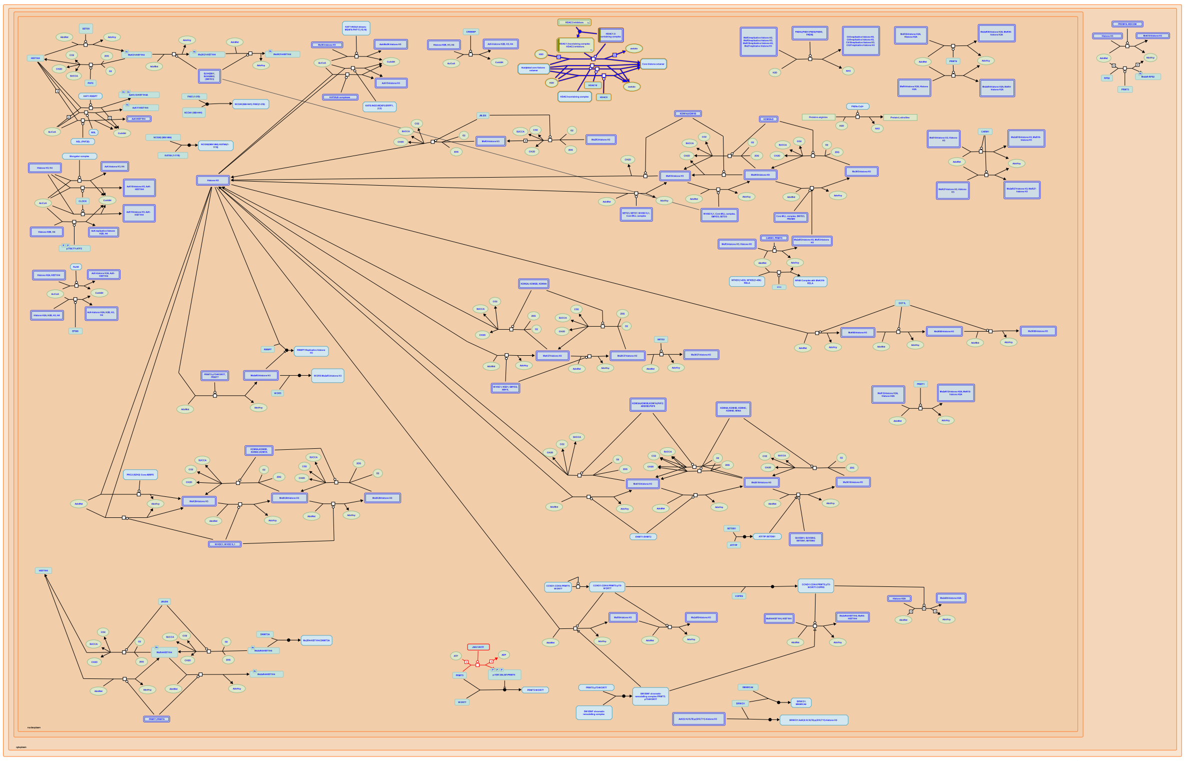

Lysine deacetylases (KDACs), historically referred to as histone deacetylases (HDACs), are divided into the Rpd3/Hda1 metal-dependent 'classical HDAC family' (de Ruijter et al. 2003, Verdin et al. 2003) and the unrelated sirtuins (Milne & Denu 2008). Phylogenetic analysis divides human KDACs into four classes (Gregoretti et al. 2004): Class I includes HDAC1, 2, 3 and 8; Class IIa includes HDAC4, 5, 7 and 9; Class IIb includes HDAC6 and 10; Class III are the sirtuins (SIRT1-7); Class IV has one member, HDAC11 (Gao et al. 2002). Class III enzymes use an NAD+ cofactor to perform deacetylation (Milne & Denu 2008, Yang & Seto 2008), the others classes use a metal-dependent mechanism (Gregoretti et al. 2004) to catalyze the hydrolysis of acetyl-L-lysine side chains in histone and non-histone proteins yielding L-lysine and acetate. X-ray crystal structures are available for four human HDACs; these structures have conserved active site residues, suggesting a common catalytic mechanism (Lombardi et al. 2011). They require a single transition metal ion and are typically studied in vitro as Zn2+-containing enzymes, though in vivo HDAC8 exhibits increased activity when substituted with Fe2+ (Gantt et al. 2006). The structurally-related enzyme acetylpolyamine amidohydrolase (APAH) (Leipe & Landsman 1997) exhibits optimal activity with Mn2+, followed closely by Zn2+ (Sakurada et al. 1996).

HDACs are often part of multi-protein transcriptional complexes that are recruited to gene promoters, regulating transcription without direct DNA binding. With the exception of HDAC8, all class I members can be catalytic subunits of multiprotein complexes (Yang & Seto 2008). HDAC1 and HDAC2 interact to form the catalytic core of several multisubunit complexes including Sin3, nucleosome remodeling deacetylase (NuRD) and corepressor of REST (CoREST) complexes (Grozinger & Schreiber 2002). HDAC3 is part of the silencing mediator of retinoic acid and thyroid hormone receptor (SMRT) complex or the homologous nuclear receptor corepressor (NCoR) (Li et al. 2000, Wen et al. 2000, Zhang et al. 2002, Yoon et al. 2003, Oberoi et al. 2011) which are involved in a wide range of processes including metabolism, inflammation, and circadian rhythms (Mottis et al. 2013).

Class IIa HDACs (HDAC4, -5, -7, and -9) shuttle between the nucleus and cytoplasm (Yang & Seto 2008, Haberland et al. 2009). The nuclear export of class IIa HDACs requires phosphorylation stimulated by calcium or other stimuli. They appear to have been evolutionarily inactivated as enzymes, having acquired a histidine substitution of the tyrosine residue in the active site of the mammalian deacetylase domain (H976 in humans) (Lahm et al. 2007, Schuetz et al. 2008). Instead they function as transcriptional corepressors for the MEF2 family of transcription factors (Yang & Gregoire 2005) .

Histones are the primary substrate for most HDACs except HDAC6 which is predominantly cytoplasmic and acts on alpha-tublin (Hubbert et al. 2002, Zhang et al. 2003, Boyault et al. 2007). HDACs also deacetylate proteins such as p53, E2F1, RelA, YY1, TFIIE, BCL6 and TFIIF (Glozak et al. 2005).

Histone deacetylases are targeted by structurally diverse compounds known as HDAC inhibitors (HDIs) (Marks et al. 2000). These can induce cytodifferentiation, cell cycle arrest and apoptosis of transformed cells (Marks et al. 2000, Bolden et al. 2006). Some HDIs have significant antitumor activity (Marks and Breslow 2007, Ma et al. 2009) and at least two are approved anti-cancer drugs.

The coordinates of post-translational modifications represented and described here follow UniProt standard practice whereby coordinates refer to the translated protein before any further processing. Histone literature typically refers to coordinates of the protein after the initiating methionine has been removed. Therefore the coordinates of post-translated residues in the Reactome database and described here are frequently +1 when compared with the literature.

References

van Gennip AH, Kemp S, Caron HN, van Kuilenburg AB & de Ruijter AJ (2003). Histone deacetylases (HDACs): characterization of the classical HDAC family. Biochem. J., 370, 737-49.

Cole KE, Dowling DP, Christianson DW & Lombardi PM (2011). Structure, mechanism, and inhibition of histone deacetylases and related metalloenzymes. Curr. Opin. Struct. Biol., 21, 735-43.

Edit history

Date	Action	Author
2013-03-12	Edited	Jupe S
2013-03-12	Authored	Jupe S
2013-03-12	Created	Jupe S
2014-05-08	Reviewed	Yang XJ
2023-11-17	Modified	Wright A

1 submitted entities found in this pathway, mapping to 2 Reactome entities

Input	UniProt Id
SAP30	O75446, Q9HAJ7

7. B-WICH complex positively regulates rRNA expression (R-HSA-5250924)

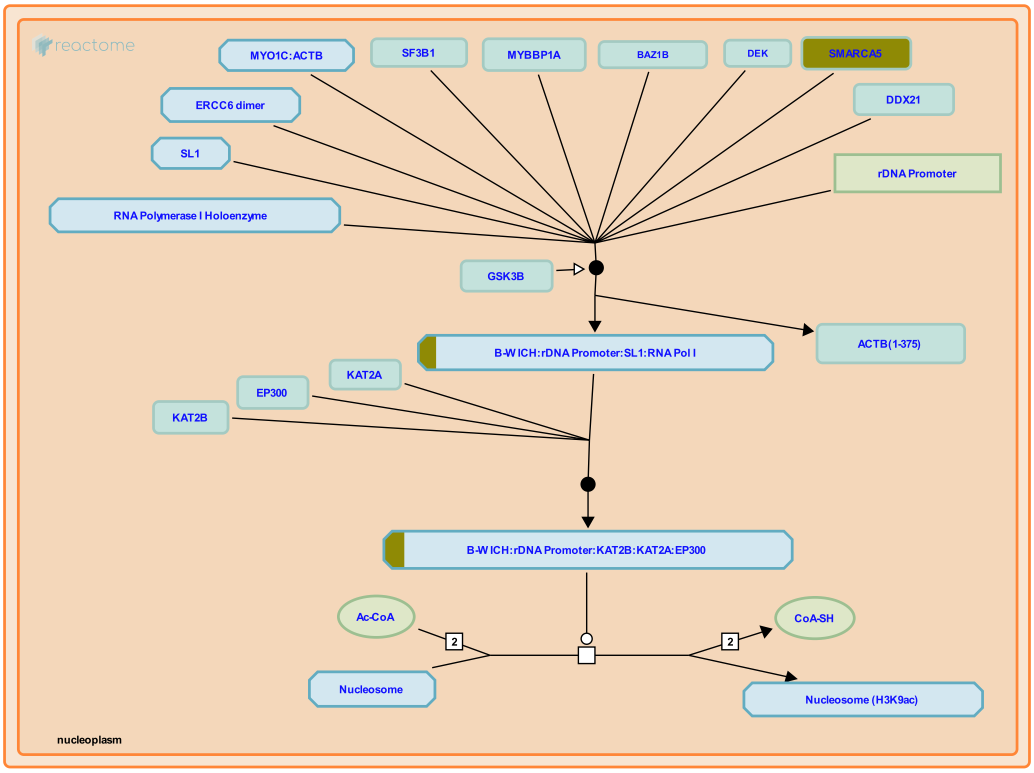

Cellular compartments: nucleoplasm.

The B-WICH complex is a large 3 Mdalton complex containing SMARCA5 (SNF2H), BAZ1B (WSTF), ERCC6 (CSB), MYO1C (Nuclear myosin 1c), SF3B1, DEK, MYBBP1A, and DDX21 (Cavellan et al. 2006, Percipalle et al. 2006, Vintermist et al. 2001, Sarshad et al. 2013, Shen et al. 2013, reviewed in Percipalle and Farrants 2006). B-WICH is found at active rRNA genes as well as at 5S rRNA and 7SL RNA genes. B-WICH appears to remodel chromatin and recruit histone acetyltransferases that modify histones to transcriptionally active states.

References

Farrants AK, Cavellán E, Percipalle P & Asp P (2006). The WSTF-SNF2h chromatin remodeling complex interacts with several nuclear proteins in transcription. J. Biol. Chem., 281, 16264-71.

Farrants AK, Scheer U, Cavellán E, Percipalle P, Voit R, Fomproix N, ... Grummt I (2006). The chromatin remodelling complex WSTF-SNF2h interacts with nuclear myosin 1 and has a role in RNA polymerase I transcription. EMBO Rep., 7, 525-30.

Böhm S, Ostlund Farrants AK, Sadeghifar F, Mansén A, Vintermist A, Percipalle P & Louvet E (2011). The chromatin remodelling complex B-WICH changes the chromatin structure and recruits histone acetyl-transferases to active rRNA genes. PLoS ONE, 6, e19184.

Farrants AK & Percipalle P (2006). Chromatin remodelling and transcription: be-WICHed by nuclear myosin 1. Curr. Opin. Cell Biol., 18, 267-74.

Lyu G, Zhou T, Zhu Q, Shen M, Zhang F, Gao Q, ... Tao W (2013). The chromatin remodeling factor CSB recruits histone acetyltransferase PCAF to rRNA gene promoters in active state for transcription initiation. PLoS ONE, 8, e62668.

Edit history

Date	Action	Author
2014-01-29	Edited	May B
2014-01-29	Authored	May B
2014-01-31	Created	May B
2015-11-07	Reviewed	Percipalle P
2023-11-16	Modified	Wright A

1 submitted entities found in this pathway, mapping to 1 Reactome entities

Input	UniProt Id
SMARCA5	O60264

Interactors found in this pathway (3)

Input	UniProt Id	Interacts with
CEBPZ	Q03701	P49841
YBX1	P67809	Q9NR30

Input	UniProt Id	Interacts with
SMARCA5	O60264	Q9UIG0

8. Negative epigenetic regulation of rRNA expression (R-HSA-5250941)

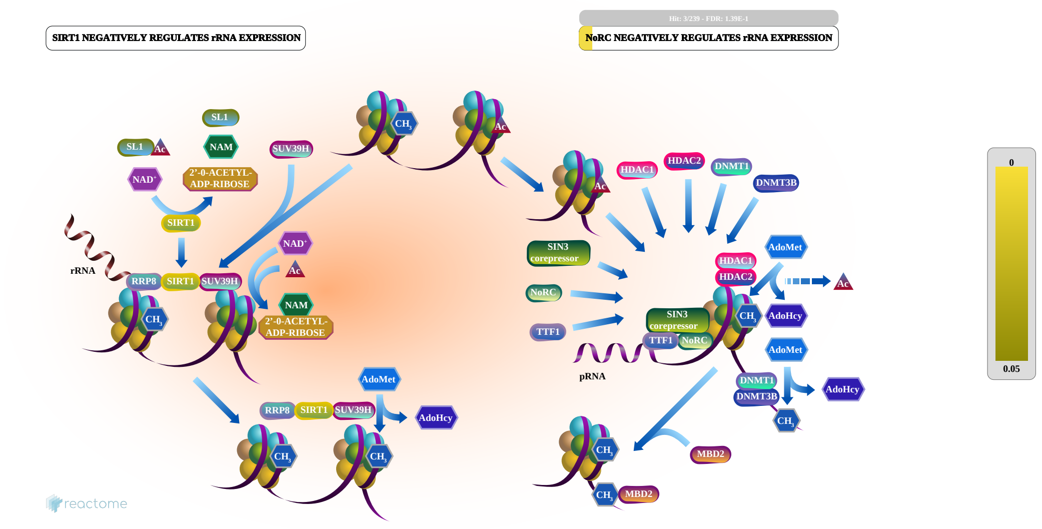

Cellular compartments: nucleoplasm.

Transcription of rRNA genes is controlled by epigenetic activation and repression (reviewed in McStay and Grummt 2008, Goodfellow and Zomerdijk 2012, Grummt and Langst 2013). About half of the roughly 400 rRNA genes are expressed and these have the modifications of active chromatin: unmethylated DNA and acetylated histones. Repressed genes generally have methylated DNA and histone H3 methylated at lysine-9. Regulators of repression include the eNoSC complex, SIRT1, and the NoRC complex.

SIRT1 negatively regulates rRNA expression as a subunit of the eNoSC complex, which deacetylates histone H3 and dimethylates lysine-9 of histone H3 (H3K9me2).

NoRC negatively regulates rRNA expression by shifting a nucleosome near the start of rRNA transcription into a more repressive location and recruiting Histone Deacetylase 1 and 2 (HDAC1, HDAC2) and DNA Methyltransferase 1 and 3b (DNMT1, DNMT3b).

References

Zomerdijk JC & Goodfellow SJ (2012). Basic mechanisms in RNA polymerase I transcription of the ribosomal RNA genes. Subcell. Biochem., 61, 211-36.

Grummt I & McStay B (2008). The epigenetics of rRNA genes: from molecular to chromosome biology. Annu Rev Cell Dev Biol, 24, 131-57.

Längst G & Grummt I (2013). Epigenetic control of RNA polymerase I transcription in mammalian cells. Biochim. Biophys. Acta, 1829, 393-404.

Edit history

Date	Action	Author
2014-01-29	Edited	May B
2014-01-29	Authored	May B
2014-01-31	Reviewed	May B

Date	Action	Author
2014-01-31	Created	May B
2023-11-17	Modified	Wright A

2 submitted entities found in this pathway, mapping to 3 Reactome entities

Input	UniProt Id	Input	UniProt Id
SAP30	O75446, Q9HAJ7	SMARCA5	O60264

Interactors found in this pathway (1)

Input	UniProt Id	Interacts with	Input	UniProt Id	Interacts with
SAP30	O75446	Q92769, Q13547			

9. Positive epigenetic regulation of rRNA expression (R-HSA-5250913)

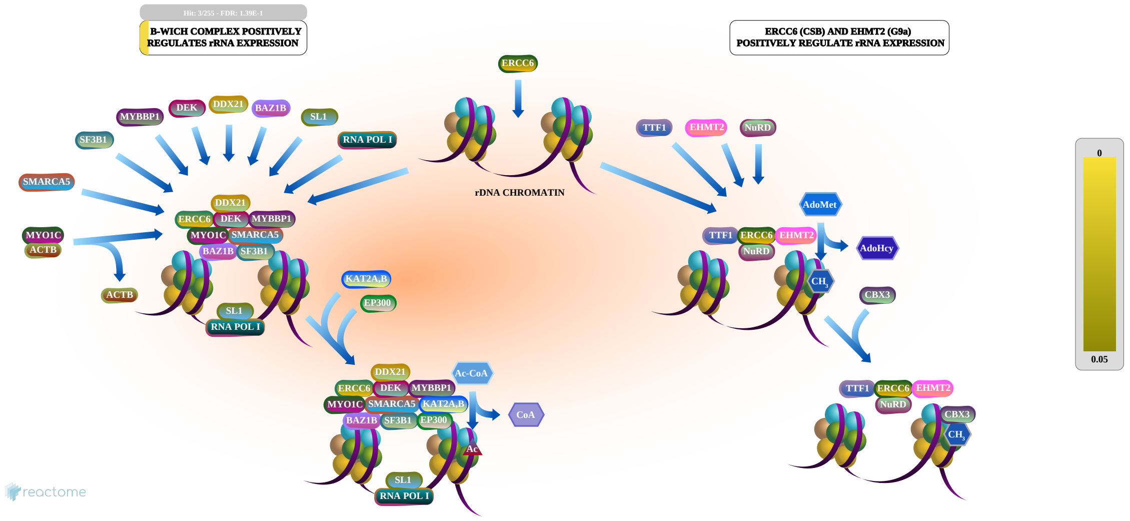

Cellular compartments: nucleoplasm.

Transcription of rRNA genes is controlled by epigenetic activation and repression according to the metabolic requirements of the cell (reviewed in Percipalle and Farrants 2006, McStay and Grummt 2008, Goodfellow and Zomerdijk 2012, Grummt and Langst 2013). Depending on the growth state of the cell, about half of the approximately 400 rRNA genes are expressed and these have the modifications characteristic of active chromatin: unmethylated DNA and acetylated histones. Repressed genes generally have methylated DNA and histone H3 methylated at lysine-9. Regulators of activation include ERCC6 (CSB), histone acetylases such as KAT2B (PCAF), and the B-WICH complex. Dysregulation of RNA polymerase I transcription plays a role in disease (reviewed in Hannan et al. 2013).

The B-WICH complex positively regulates rRNA expression by remodeling chromatin and recruiting histone acetyltransferases that modify histones to transcriptionally active states

ERCC6 (CSB) and EHMT2(G9a) positively regulate rRNA expression by ERCC6 recruiting the histone methyltransferase EHMT2 (also known as G9a) which dimethylates histone H3 at lysine-9 within the transcribed regions of rRNA genes.

ERCC6 (CSB) and KAT2B (PCAF) positively regulate rRNA expression by ERCC6 recruiting the histone acetyltransferase KAT2B to the promoter where KAT2B acetylates histone H4 at several lysine residues and histone H3 at lysine-9. The acetylated chromatin facilitates the assembly of RNA polymerase I initiation complex.

References

Zomerdijk JC & Goodfellow SJ (2012). Basic mechanisms in RNA polymerase I transcription of the ribosomal RNA genes. Subcell. Biochem., 61, 211-36.

Farrants AK & Percipalle P (2006). Chromatin remodelling and transcription: be-WICHed by nuclear myosin 1. Curr. Opin. Cell Biol., 18, 267-74.

Rothblum LI, Pearson RB, Sanij E, Hannan RD & Hannan KM (2013). Dysregulation of RNA polymerase I transcription during disease. Biochim. Biophys. Acta, 1829, 342-60.

Grummt I & McStay B (2008). The epigenetics of rRNA genes: from molecular to chromosome biology. Annu Rev Cell Dev Biol, 24, 131-57.

Längst G & Grummt I (2013). Epigenetic control of RNA polymerase I transcription in mammalian cells. Biochim. Biophys. Acta, 1829, 393-404.

Edit history

Date	Action	Author
2014-01-29	Edited	May B
2014-01-29	Authored	May B
2014-01-31	Created	May B
2015-11-07	Reviewed	Percipalle P
2023-11-16	Modified	Wright A

1 submitted entities found in this pathway, mapping to 1 Reactome entities

Input	UniProt Id
SMARCA5	O60264

Interactors found in this pathway (3)

Input	UniProt Id	Interacts with
CEBPZ	Q03701	P49841
YBX1	P67809	Q9NR30

Input	UniProt Id	Interacts with
SMARCA5	O60264	Q9UIG0

10. Chromatin organization (R-HSA-4839726)

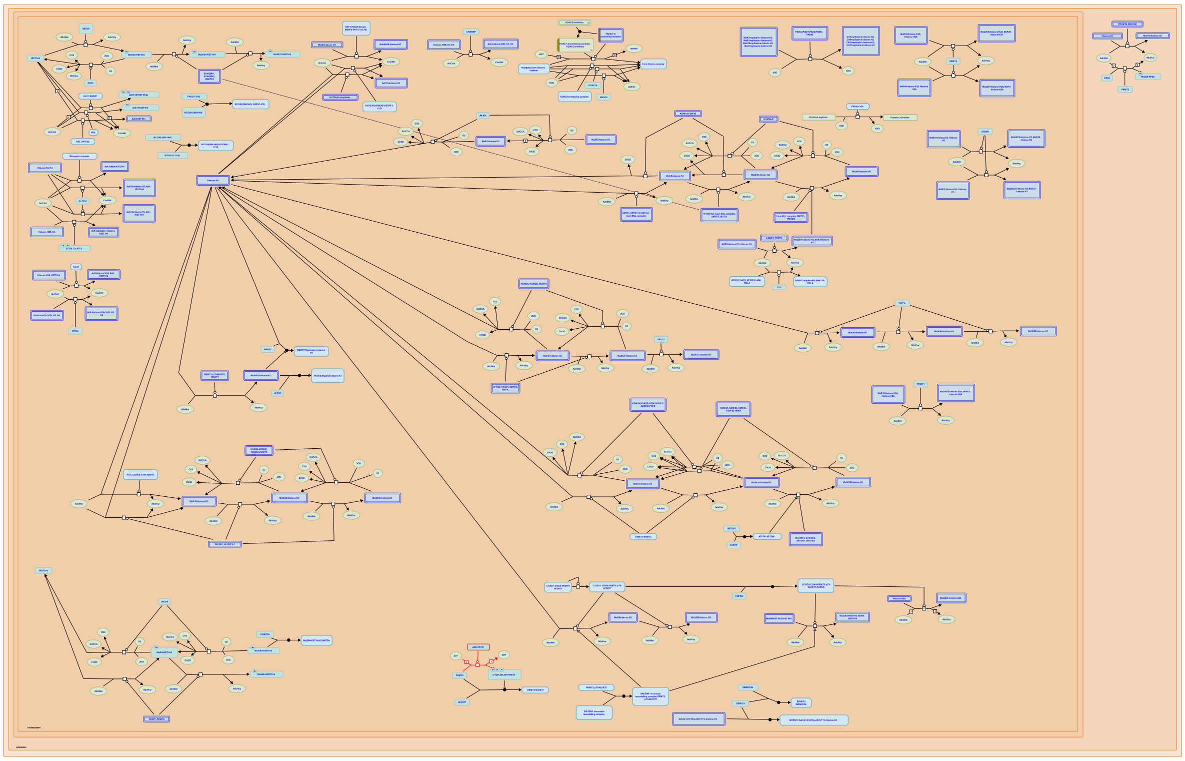

Cellular compartments: nucleoplasm.

Chromatin organization refers to the composition and conformation of complexes between DNA, protein and RNA. It is determined by processes that result in the specification, formation or maintenance of the physical structure of eukaryotic chromatin. These processes include histone modification, DNA modification, and transcription. The modifications are bound by specific proteins that alter the conformation of chromatin.

References

Gilchrist S, Bickmore WA & Gilbert N (2005). Chromatin organization in the mammalian nucleus. Int. Rev. Cytol., 242, 283-336.

Reinberg D & Li G (2011). Chromatin higher-order structures and gene regulation. Curr. Opin. Genet. Dev., 21, 175-86.

Edit history

Date	Action	Author
2013-11-02	Edited	May B
2013-11-02	Authored	May B
2013-11-02	Created	May B
2013-11-18	Reviewed	Karagiannis T
2023-11-17	Modified	Wright A

1 submitted entities found in this pathway, mapping to 2 Reactome entities

Input	UniProt Id
SAP30	O75446, Q9HAJ7

Interactors found in this pathway (3)

Input	UniProt Id	Interacts with
PDE9A	O76083-2	P61964
SMARCA5	O60264	P62805
SAP30	O75446	Q16576

11. Chromatin modifying enzymes (R-HSA-3247509)

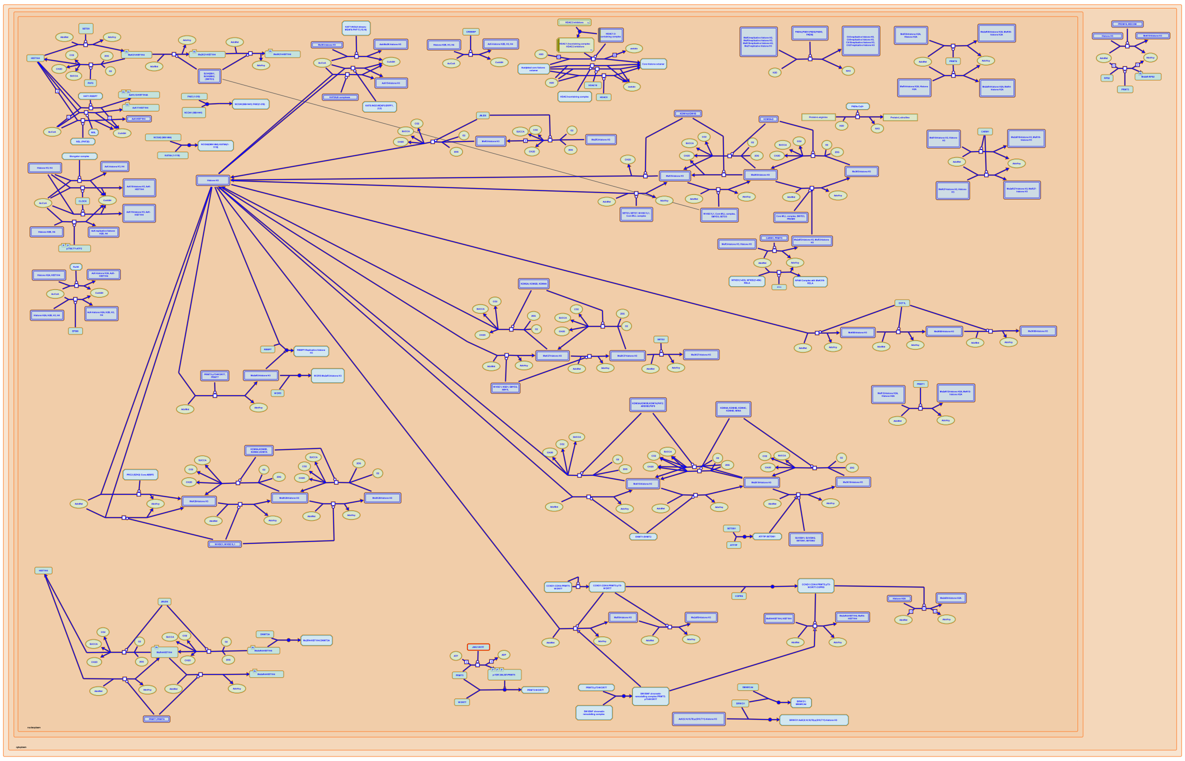

Eukaryotic DNA is associated with histone proteins and organized into a complex nucleoprotein structure called chromatin. This structure decreases the accessibility of DNA but also helps to protect it from damage. Access to DNA is achieved by highly regulated local chromatin decondensation.

The 'building block' of chromatin is the nucleosome. This contains ~150 bp of DNA wrapped around a histone octamer which consists of two each of the core histones H2A, H2B, H3 and H4 in a 1.65 left-handed superhelical turn (Luger et al. 1997, Andrews & Luger 2011).

Most organisms have multiple genes encoding the major histone proteins. The replication-dependent genes for the five histone proteins are clustered together in the genome in all metazoans. Human replication-dependent histones occur in a large cluster on chromosome 6 termed HIST1, a smaller cluster HIST2 on chromosome 1q21, and a third small cluster HIST3 on chromosome 1q42 (Marzluff et al. 2002). Histone genes are named systematically according to their cluster and location within the cluster.

The 'major' histone genes are expressed primarily during the S phase of the cell cycle and code for the bulk of cellular histones. Histone variants are usually present as single-copy genes that are not restricted in their expression to S phase, contain introns and are often polyadenylated (Old & Woodland 1984). Some variants have significant differences in primary sequence and distinct biophysical characteristics that are thought to alter the properties of nucleosomes. Others localize to specific regions of the genome. Some variants can exchange with pre-existing major histones during development and differentiation, referred to as replacement histones (Kamakaka & Biggins 2005). These variants can become the predominant species in differentiated cells (Pina & Suau 1987, Wunsch et al. 1991). Histone variants may have specialized functions in regulating chromatin dynamics.

The H2A histone family has the highest sequence divergence and largest number of variants. H2A.Z and H2A.XH2A are considered 'universal variants', found in almost all organisms (Talbert & Henikoff 2010). Variants differ mostly in the C-terminus, including the docking domain, implicated in interactions with the (H3-H4)x2 tetramer within the nucleosome, and in the L1 loop, which is the interaction interface of H2A-H2B dimers (Bonisch & Hake 2012). Canonical H2A proteins are expressed almost exclusively during S-phase. There are several nearly identical variants (Marzluff et al. 2002). No functional specialization of these canonical H2A isoforms has been demonstrated (Bonisch & Hake 2012). Reversible histone modifications such as acetylation and methylation regulate transcription from genomic DNA, defining the 'readability' of genes in specific tissues (Kouzarides 2007, Marmorstein & Trievel 2009, Butler et al. 2012).

N.B. The coordinates of post-translational modifications represented here follow Reactome standardized naming, which includes the UniProt standard practice whereby coordinates refer to the translated protein before any further processing. Histone literature typically refers to coordinates of the protein after the initiating methionine has been removed; therefore the coordinates of post-translated histone residues described here are frequently +1 when compared with the literature. For more information on Reactome's standards for naming pathway events, the molecules that participate in them and representation of post-translational modifications, please refer to Naming Conventions on the Reactome Wiki or Jupe et al. 2014.

References

Edit history

Date	Action	Author
2013-03-12	Authored	Jupe S
2013-04-04	Created	Jupe S
2013-11-18	Edited	Jupe S
2013-11-18	Reviewed	Karagiannis T
2023-11-17	Modified	Wright A

1 submitted entities found in this pathway, mapping to 2 Reactome entities

Input	UniProt Id
SAP30	O75446, Q9HAJ7

Interactors found in this pathway (3)

Input	UniProt Id	Interacts with	Input	UniProt Id	Interacts with
PDE9A	O76083-2	P61964	SAP30	O75446	Q16576
SMARCA5	O60264	P62805			

12. TFAP2 (AP-2) family regulates transcription of other transcription factors (R-HSA-8866906)

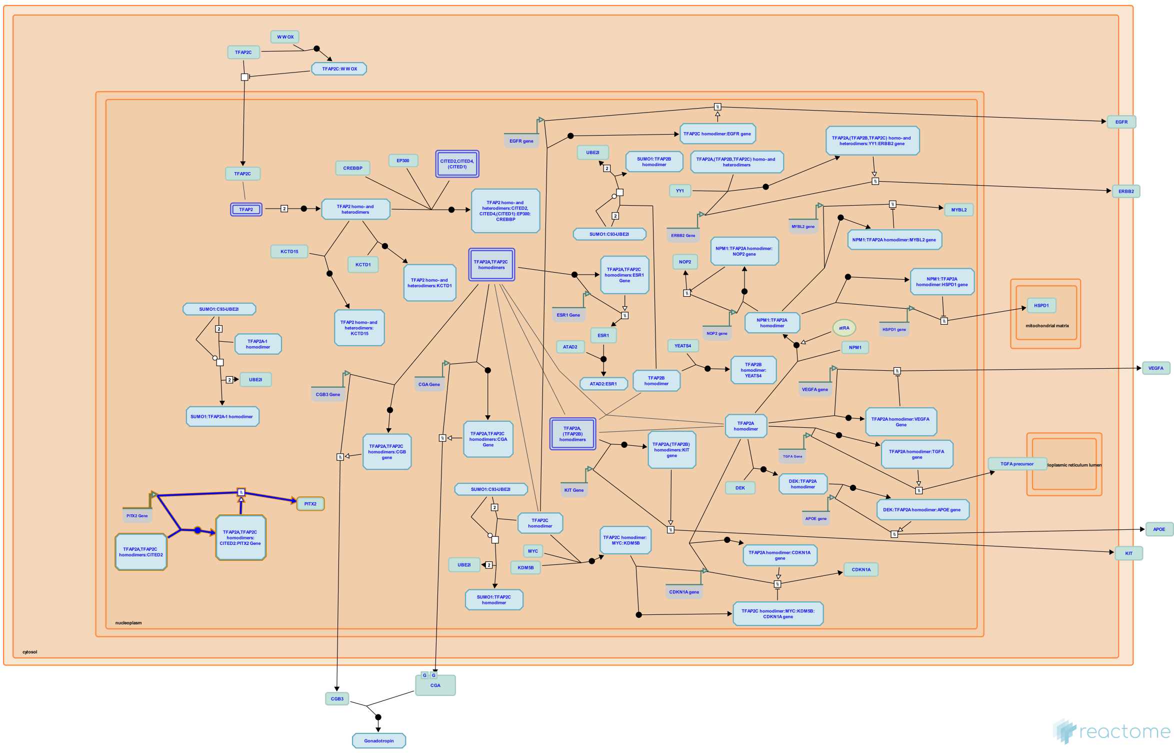

Homodimers and possibly heterodimers of TFAP2A and TFAP2C, in complex with CITED2, stimulate transcription of the PITX2 gene, involved in left-right patterning and heart development (Bamforth et al. 2004, Li et al. 2012).

References

Anderson RH, Bhattacharya S, Farthing CR, Broadbent C, Neubauer S, Bragança J, ... Brown NA (2004). Cited2 controls left-right patterning and heart development through a Nodal-Pitx2c pathway. Nat. Genet., 36, 1189-96.

Pan H, Guan L, Li Q, Su D & Ma X (2012). CITED2 mutation links congenital heart defects to dysregulation of the cardiac gene VEGF and PITX2C expression. Biochem. Biophys. Res. Commun., 423, 895-9.

Edit history

Date	Action	Author
2016-03-14	Edited	Orlic-Milacic M
2016-03-14	Authored	Orlic-Milacic M
2016-04-04	Created	Orlic-Milacic M
2016-05-04	Reviewed	Dawid IB, Zarelli VE
2016-05-17	Reviewed	Bogachek MV, Weigel RJ
2023-03-08	Modified	Matthews L

Interactors found in this pathway (1)

Input	UniProt Id	Interacts with	Input	UniProt Id	Interacts with
YBX1	P67809	Q99697			

13. RMTs methylate histone arginines (R-HSA-3214858)

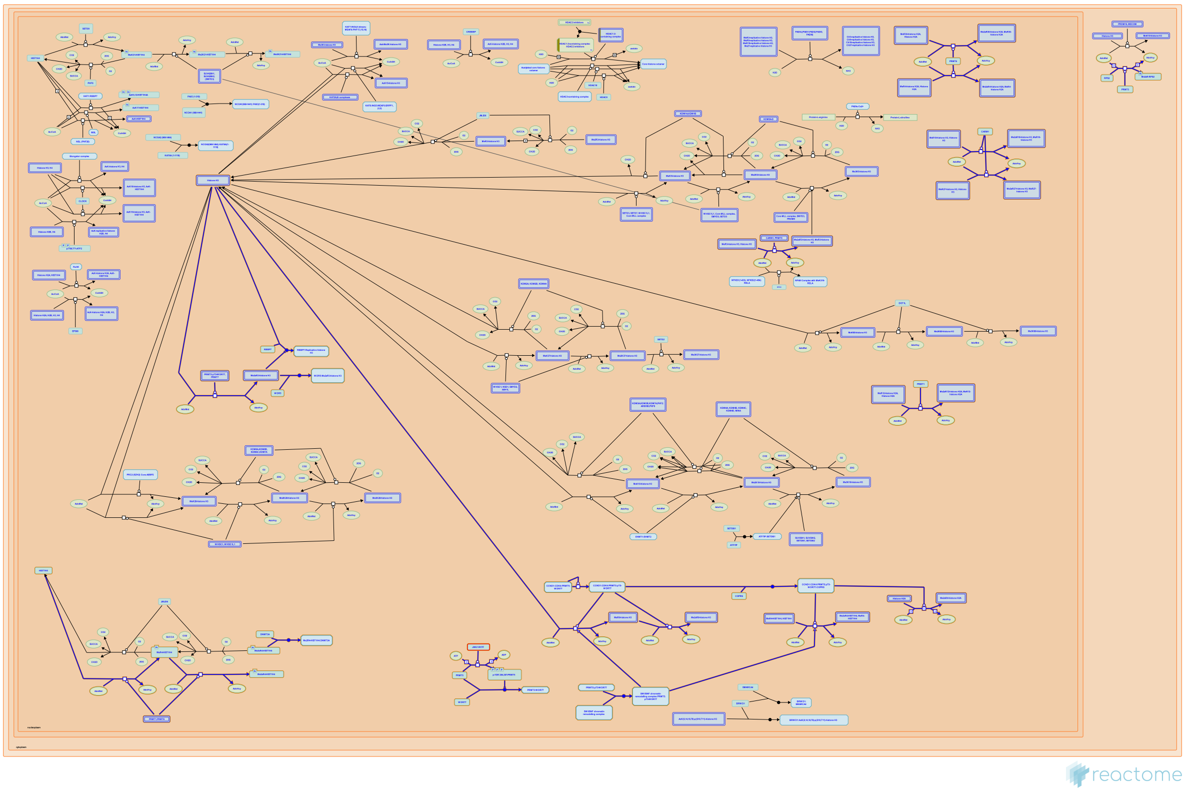

Arginine methylation is a common post-translational modification; around 2% of arginine residues are methylated in rat liver nuclei (Boffa et al. 1977). Arginine can be methylated in 3 different ways: monomethylarginine (MMA); NG,NG-asymmetric dimethylarginine (ADMA) and NG,N'G-symmetric dimethylarginine (SDMA). The formation of MMA, ADMA and SDMA in mammalian cells is carried out by members of a family of nine protein arginine methyltransferases (PRMTs) (Bedford & Clarke 2009).

Type I, II and III PRMTs generate MMA on one of the two terminal guanidino nitrogen atoms. Subsequent generation of asymmetric dimethylarginine (ADMA) is catalysed by the type I enzymes PRMT1, PRMT2, PRMT3, co-activator-associated arginine methyltransferase 1 (CARM1), PRMT6 and PRMT8. Production of symmetric dimethylarginine (SDMA) is catalysed by the type II enzymes PRMT5 and PRMT7. On certain substrates, PRMT7 also functions as a type III enzyme, generating MMA only. PRMT9 activity has not been characterized. No known enzyme is capable of both ADMA and SDMA modifications. Arginine methylation is regarded as highly stable; no arginine demethylases are known (Yang & Bedford 2013).

Most PRMTs methylate glycine- and arginine-rich (GAR) motifs in their substrates (Boffa et al. 1977). CARM1 methylates a proline-, glycine- and methionine-rich (PGM) motif (Cheng et al. 2007). PRMT5 can dimethylate arginine residues in GAR and PGM motifs (Cheng et al. 2007, Branscombe et al. 2001).

PRMTs are widely expressed and are constitutively active as purified recombinant proteins. However, PRMT activity can be regulated through PTMs, association with regulatory proteins, subcellular compartmentalization and factors that affect enzyme-substrate interactions. The target sites of PRMTs are influenced by the presence of other PTMs on their substrates. The best characterized examples of this are for histones. Histone H3 lysine-19 acetylation (H3K18ac) primes the histone tail for asymmetric dimethylation at arginine-18 (H3R17me2a) by CARM1 (An et al. 2003, Daujat et al. 2002, Yue et al. 2007). H3 lysine-10 acetylation (H3K9ac) blocks arginine-9 symmetric dimethylation (H3R8me2s) by PRMT5 (Pal et al. 2004). H4R3me2a catalyzed by PRMT1 favours subsequent acetylation of the histone H4 tail (Huang et al. 2005). At the same time histone H4 lysine-5 acetylation (H4K5ac) makes the H4R3 motif a better substrate for PRMT5 compared with PRMT1, thereby moving the balance from an activating ADMA mark to a suppressive SDMA mark at the H4R3 motif (Feng et al. 2011). Finally methylation of Histone H3 on arginine-3 (H3R2me2a) by PRMT6 blocks methylation of H3 lysine-5 by the MLL complex (H3K4me3), and vice versa, methylation of H3K4me3 prevents H3R2me2a methylation (Guccione et al. 2007, Kirmizis et al. 2007, Hyllus et al. 2007).

N.B. The coordinates of post-translational modifications represented and described here follow UniProt standard practice whereby coordinates refer to the translated protein before any further processing. Histone literature typically refers to coordinates of the protein after the initiating methionine has been removed. Therefore the coordinates of post-translated residues in the Reactome database and described here are frequently +1 when compared with the literature.

References

Bedford MT & Yang Y (2013). Protein arginine methyltransferases and cancer. Nat. Rev. Cancer, 13, 37-50.

Bedford MT & Clarke SG (2009). Protein arginine methylation in mammals: who, what, and why. Mol. Cell, 33, 1-13.

Edit history

Date	Action	Author
2013-03-12	Authored	Jupe S
2013-03-12	Created	Jupe S
2013-03-15	Edited	Jupe S
2014-05-09	Reviewed	Guccione E
2014-07-23	Reviewed	Fischle W
2023-11-17	Modified	Wright A

Interactors found in this pathway (3)

Input	UniProt Id	Interacts with
PDE9A	O76083-2	P61964
SMARCA5	O60264	P62805

Input	UniProt Id	Interacts with
SAP30	O75446	Q16576

14. Inositol transporters (R-HSA-429593)

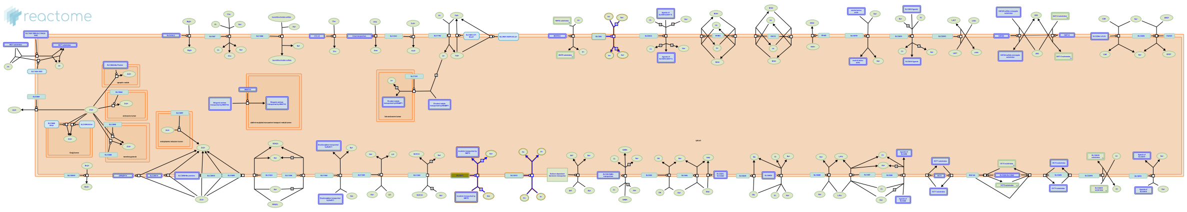

Myo-Inositol is a neutral cyclic polyol, abundant in mammalian tissues. It plays important roles; it is a precursor to phosphatidylinositols (PtdIns) and to the inositol phosphates (IP), which serve as second messengers and as key regulators of many cell functions. It can also serve as a compatible osmolyte during volume regulation in many tissues where cells are exposed to hyperosmotic conditions. Three members of the glucose transporter families are inositol transporters. Two (SMIT1 and SMIT2) couple myo-inositol transport with two Na+ ions. Unlike SMIT1, SMIT2 also transports D-chiro_inositol. The third transporter (HMIT), couples myo-inositol transport with a proton.

References

Edit history

Date	Action	Author
2009-07-17	Edited	Jassal B
2009-07-17	Authored	Jassal B
2009-07-17	Created	Jassal B
2009-08-24	Reviewed	He L
2023-11-17	Modified	Wright A

1 submitted entities found in this pathway, mapping to 1 Reactome entities

Input	UniProt Id
SLC5A11	Q8WWX8

15. Translesion synthesis by Y family DNA polymerases bypasses lesions on DNA template (R-HSA-110313)

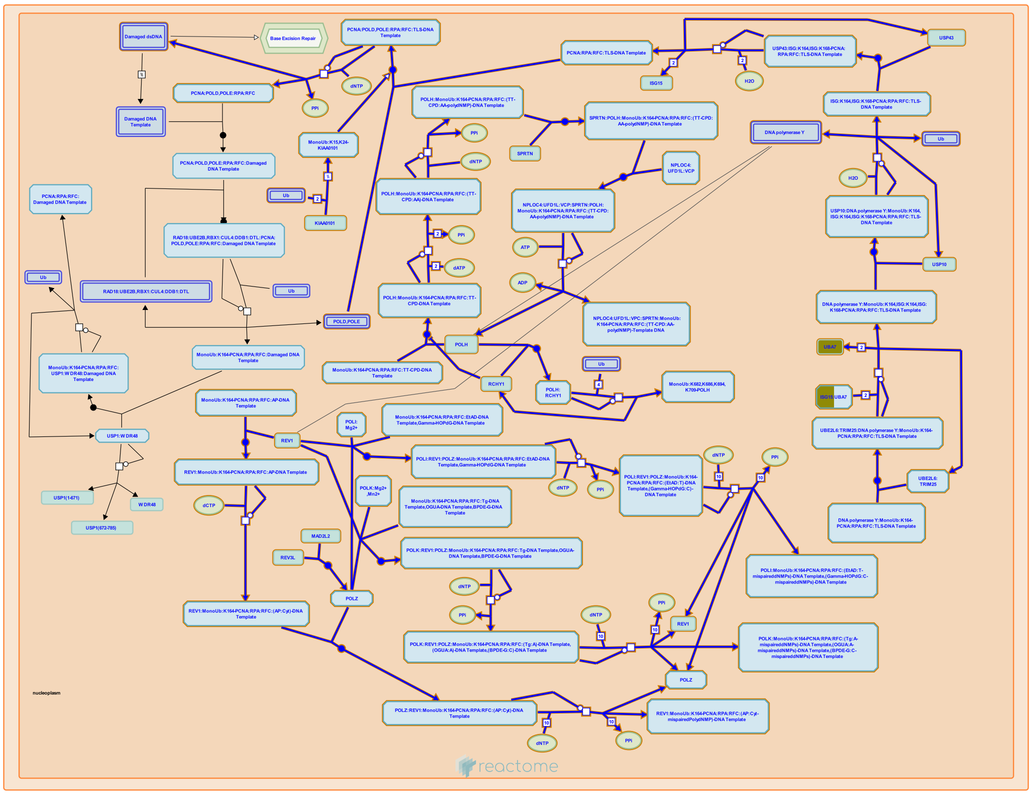

Cellular compartments: nucleoplasm.

Ubiquitous environmental and endogenous genotoxic agents cause DNA lesions that can interfere with normal DNA metabolism including DNA replication, eventually resulting in mutations that lead to carcinogenesis and/or cell death. Cells possess repair mechanisms like nucleotide excision and base excision repair pathways to maintain the integrity of the genome. However, some types of lesions are repaired very inefficiently and others may not be recognized and repaired before the lesion-containing DNA undergoes DNA replication. To prevent acute cell death through arrested DNA replication at unrepaired lesions, cells have a mechanism, referred to as translesion synthesis (TLS), which allows DNA synthesis to proceed past lesions. TLS depends on the Y family of DNA polymerases (Lindahl and Wood 1999, Masutani et al. 2000, Yang 2014).

References

Lindahl T & Wood RD (1999). Quality control by DNA repair. Science, 286, 1897-905.

Yang W (2014). An overview of Y-Family DNA polymerases and a case study of human DNA polymerase ?. Biochemistry, 53, 2793-803.

Kusumoto R, Iwai S, Masutani C & Hanaoka F (2000). Mechanisms of accurate translesion synthesis by human DNA polymerase eta. EMBO J, 19, 3100-9.

Edit history

Date	Action	Author
2004-01-29	Authored	Gopinathrao G
2004-01-29	Created	Gopinathrao G

Date	Action	Author
2014-12-11	Revised	Orlic-Milacic M
2014-12-11	Edited	Orlic-Milacic M
2015-01-07	Reviewed	Borowiec JA
2023-11-17	Modified	Wright A

1 submitted entities found in this pathway, mapping to 1 Reactome entities

Input	UniProt Id
UBA7	P41226

Interactors found in this pathway (2)

Input	UniProt Id	Interacts with	Input	UniProt Id	Interacts with
UBA7	P41226	P05161	YBX1	P67809	Q14694

16. DNA Damage Bypass (R-HSA-73893)

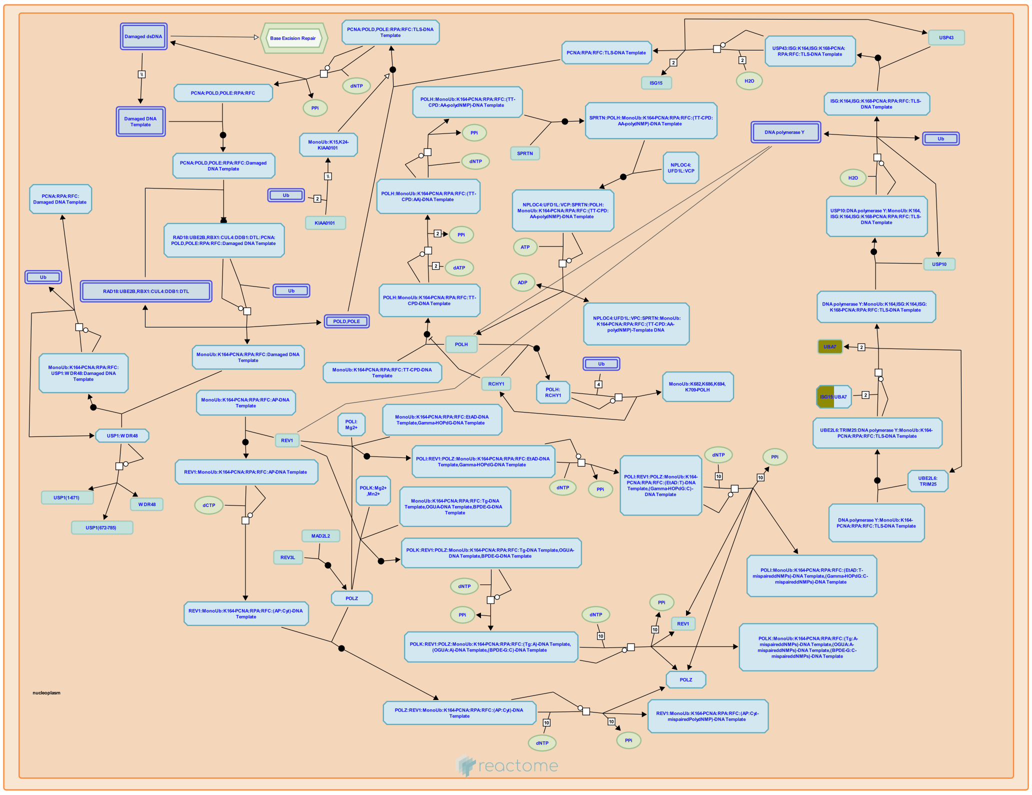

Cellular compartments: nucleoplasm.

In addition to various processes for removing lesions from the DNA, cells have developed specific mechanisms for tolerating unrepaired damage during the replication of the genome. These mechanisms are collectively called DNA damage bypass pathways. The Y family of DNA polymerases plays a key role in DNA damage bypass.

Y family DNA polymerases, REV1, POLH (DNA polymerase eta), POLK (DNA polymerase kappa) and POLI (DNA polymerase iota), as well as the DNA polymerase zeta (POLZ) complex composed of REV3L and MAD2L2, are able to carry out translesion DNA synthesis (TLS) or replicative bypass of damaged bases opposite to template lesions that arrest high fidelity, highly processive replicative DNA polymerase complexes delta (POLD) and epsilon (POLE). REV1, POLH, POLK, POLI and POLZ lack 3'->5' exonuclease activity and exhibit low fidelity and weak processivity. The best established TLS mechanisms are annotated here. TLS details that require substantial experimental clarification have been omitted. For recent and past reviews of this topic, please refer to Lehmann 2000, Friedberg et al. 2001, Zhu and Zhang 2003, Takata and Wood 2009, Ulrich 2011, Saugar et al. 2014.

References

Saugar I, Tercero JA & Ortiz-Bazán MA (2014). Tolerating DNA damage during eukaryotic chromosome replication. Exp. Cell Res., 329, 170-177.

Ulrich HD (2011). Timing and spacing of ubiquitin-dependent DNA damage bypass. FEBS Lett., 585, 2861-7.

Lehmann AR (2000). Replication of UV-damaged DNA: new insights into links between DNA polymerases, mutagenesis and human disease. Gene, 253, 1-12.

Kisker C, Friedberg EC & Fischhaber PL (2001). Error-prone DNA polymerases: novel structures and the benefits of infidelity. Cell, 107, 9-12.

Wood RD & Takata K (2009). Bypass specialists operate together. EMBO J., 28, 313-4.

Edit history

Date	Action	Author
2003-10-15	Created	Gopinathrao G
2004-02-02	Authored	Gopinathrao G
2014-12-11	Revised	Orlic-Milacic M
2014-12-11	Edited	Orlic-Milacic M
2014-12-11	Authored	Orlic-Milacic M
2015-01-07	Reviewed	Borowiec JA
2023-11-17	Modified	Wright A

1 submitted entities found in this pathway, mapping to 1 Reactome entities

Input	UniProt Id
UBA7	P41226

Interactors found in this pathway (2)

Input	UniProt Id	Interacts with	Input	UniProt Id	Interacts with
UBA7	P41226	P05161	YBX1	P67809	Q14694

17. Inhibition of Host mRNA Processing and RNA Silencing (R-HSA-168315)

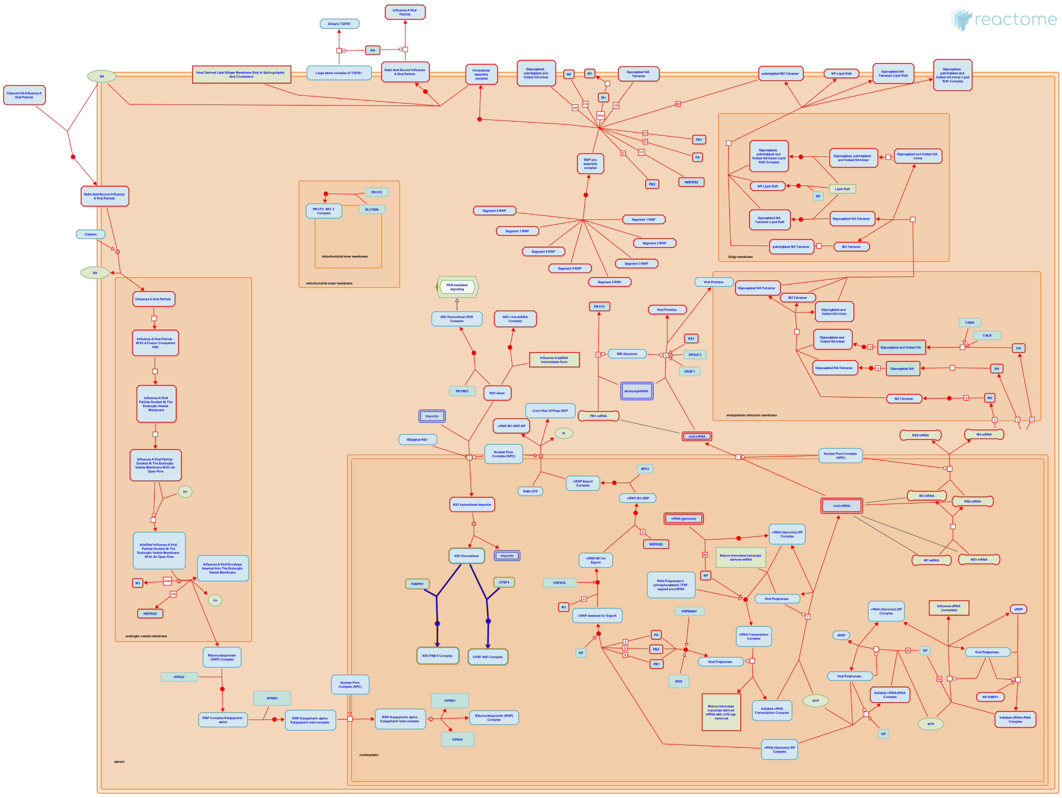

Diseases: influenza.

The Influenza Virus NS1 protein inhibits the cleavage and polyadenylation specificity factor CPSF and the PABII components of the host cell 3' end processing machinery, preventing efficient 3' end processing of host pre-mRNAs. NS1 also inhibits the splicing of pre-mRNAs, resulting in their retention within the host cell nucleus.

References

Chen Z, Krug RM & Li Y (1999). Influenza A virus NS1 protein targets poly(A)-binding protein II of the cellular 3'-end processing machinery. EMBO J, 18, 2273-83.

Lu Y, Krug RM & Qian XY (1994). The influenza virus NS1 protein: a novel inhibitor of pre-mRNA splicing. Genes Dev, 8, 1817-28.

Beloso A, Ortin J & Fortes P (1994). Influenza virus NS1 protein inhibits pre-mRNA splicing and blocks mRNA nucleocytoplasmic transport. EMBO J, 13, 704-12.

Edit history

Date	Action	Author
2004-05-12	Reviewed	Gale M Jr
2007-04-17	Created	
2013-11-18	Edited	Gillespie ME
2013-11-18	Authored	Gillespie ME
2023-10-12	Modified	Weiser JD

Interactors found in this pathway (1)

Input	UniProt Id	Interacts with	Input	UniProt Id	Interacts with
YBX1	P67809	Q86U42			

18. Z-decay: degradation of maternal mRNAs by zygotically expressed factors (R-HSA-9820865)

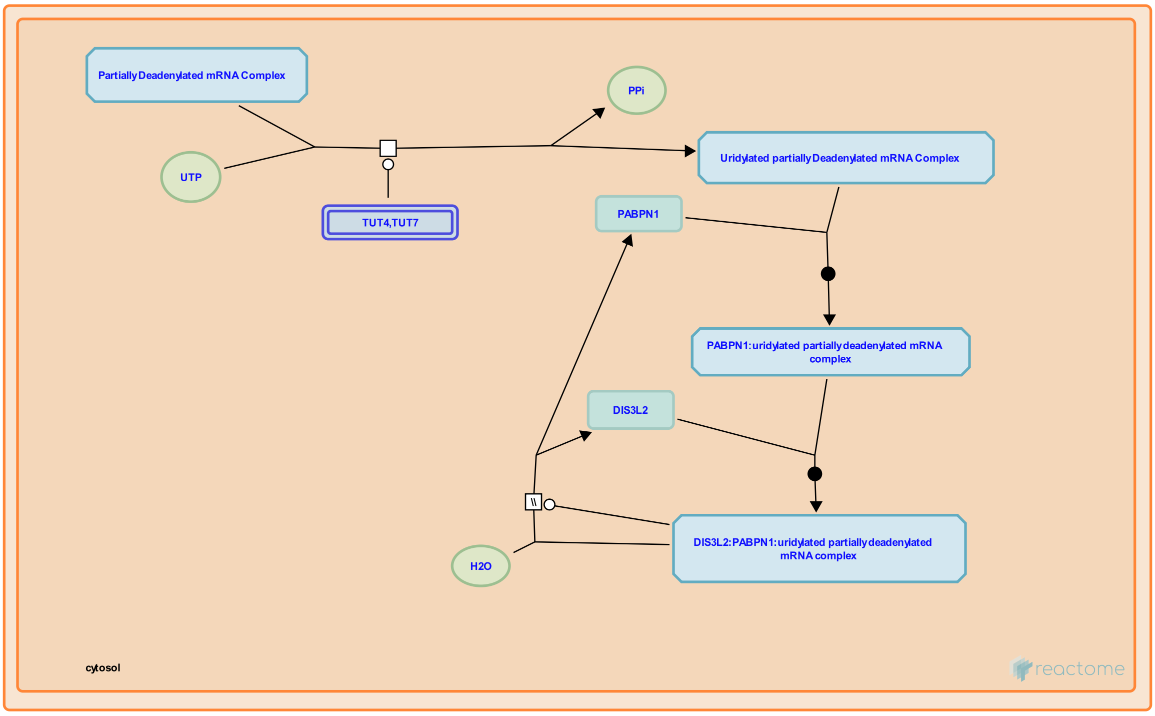

Cellular compartments: cytosol.

Maternal transcripts accumulate in the oocyte during oogenesis. Subsets of maternal transcripts are degraded during later development of the unfertilized oocyte and after fertilization of the oocyte. Zygotic decay (Z-decay) refers to the degradation of maternal transcripts by factors expressed by the zygotic genome after fertilization. In the zygote the YAP1:TEAD4 complex activates expression of TUT4 and TUT7 which then uridylate the 3' ends of specific, partially deadenylated maternal transcripts (inferred from mouse zygotes in Sha et al. 2020). The terminal uridylate residues recruit PABPN1 which recruits the 3'-5' ribonuclease DIS3L2 to degrade the mRNA (inferred from mouse homologs in Zhao et al. 2022). Absence of TUT4, TUT7, or PABPN1 results in altered mRNA abundances (inferred from mouse zygotes in Morgan et al. 2017, Sha et al. 2020, Zhao et al. 2022) and infertility (Morgan et al. 2017, Zhao et al. 2022). BTG4 expressed in oocytes and present in zygotes also plays a role in Z-decay possibly by recruiting the CCR4-NOT complex to deadenylate mRNAs prior to uridylation (inferred from mouse zygotes in Sha et al. 2020). Similar patterns of expression and mRNA decay are observed in human and mouse zygotes (Sha et al. 2020).

References

Zhao LW, Fan HY, Zhu YZ, Shen L, Pi SB & Wu YW (2022). Nuclear poly(A) binding protein 1 (PABPN1) mediates zygotic genome activation-dependent maternal mRNA clearance during mouse early embryonic development. Nucleic Acids Res, 50, 458-472.

Li S, Fan HY, Zhang S, Zheng W, Lin G, Ou XH, ... Guo L (2020). Dynamics and clinical relevance of maternal mRNA clearance during the oocyte-to-embryo transition in humans. Nat Commun, 11, 4917.

Azzi C, Much C, DiGiacomo M, Morgan M, Vitsios DM, Moreira PN, ... Collier P (2017). mRNA 3' uridylation and poly(A) tail length sculpt the mammalian maternal transcriptome. Nature, 548, 347-351.

Li S, Fan HY, Zhu YZ, Shen L, Ou XH, Chen L, ... Jiang Y (2020). Characterization of zygotic genome activation-dependent maternal mRNA clearance in mouse. Nucleic Acids Res, 48, 879-894.

Edit history

Date	Action	Author
2022-11-20	Edited	May B
2022-11-20	Authored	May B
2022-11-21	Created	May B
2023-08-08	Modified	May B
2023-08-08	Reviewed	Xie W

Interactors found in this pathway (1)

Input	UniProt Id	Interacts with	Input	UniProt Id	Interacts with
YBX1	P67809	Q86U42			

19. cGMP effects (R-HSA-418457)

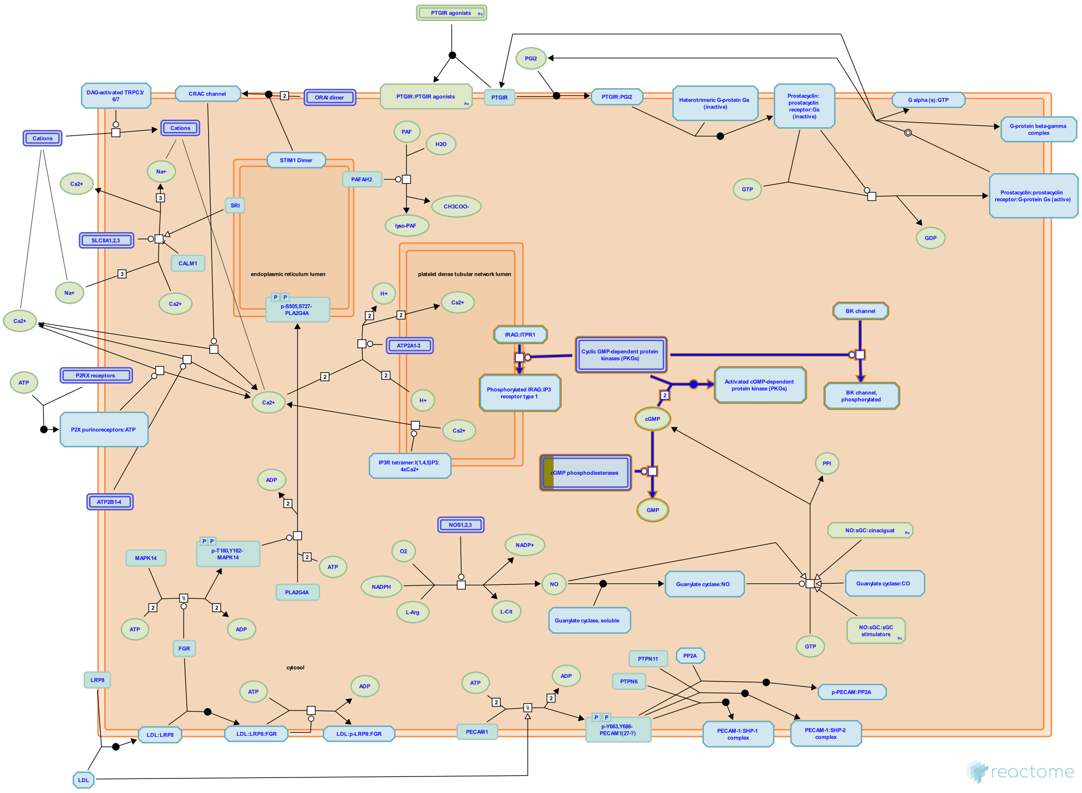

Cellular compartments: cytosol.

Cyclic guanosine monophosphate (cGMP) is an important secondary messenger synthesized by guanylate cyclases. cGMP has effects on phosphodiesterases (PDE), ion-gated channels, and the cG-MP-dependent protein kinases (cGK, Protein Kinase G or PKG). It is involved in regulation of several physiological functions including vasodilation, platelet aggregation and neurotransmission. Elevation of intracellular cGMP activates PKG (Haslam et al. 1999) which regulates several intracellular molecules and pathways including the vasodilator-stimulated phosphoprotein (VASP) (Halbrugge et al. 1990) and the ERK pathway (Hood and Granger 1998, Li et al. 2001). cGMP mediates nitric oxide (NO)-induced vascular smooth muscle relaxation (Furchgott and Vanhoutte 1989). Phosphodiesterase 5 (PDE5) hydrolyzes cGMP; the PDE5 inhibitor sildenafil (Viagra) increases intracellular cGMP and thereby can be used as a treatment for erectile dysfunction (Corbin and Francis 1999). The role of the cGMP and PKG in platelet activation was controversial as increases in platelet cGMP levels were observed in response to both platelet agonists (thrombin, ADP or collagen) and inhibitors (NO donors such as sodium nitroprusside), but it is currently accepted that PKG inhibits platelet activation (Haslam et al. 1999). Consistent with this, nitric oxide (NO) donors that inhibit platelet activation enhance intracellular cGMP (Haslam et al. 1999). cGMP also plays an important stimulatory role in GPIb-IX-mediated platelet activation. Platelet responses to cGMP have been proposed to be biphasic, consisting of an early stimulatory response that promotes platelet activation followed by a delayed platelet inhibition that serves to limit the size of platelet aggregates (Li et al 2003).

References

Murad F, Warner TD, Sheng H & Mitchell JA (1994). Effects of cyclic GMP on smooth muscle relaxation. Adv Pharmacol, 26, 171-94.

Hofmann F, Mülsch A, Walter U, Lohmann SM, Münzel T & Feil R (2003). Physiology and pathophysiology of vascular signaling controlled by guanosine 3',5'-cyclic monophosphate-dependent protein kinase [corrected]. Circulation, 108, 2172-83.

Edit history

Date	Action	Author
2009-04-22	Created	Jupe S
2009-06-03	Authored	Akkerman JW
2010-06-07	Edited	Jupe S
2010-06-07	Reviewed	Kunapuli SP
2023-11-17	Modified	Wright A

1 submitted entities found in this pathway, mapping to 1 Reactome entities

Input	UniProt Id
PDE9A	O76083

20. Insulin-like Growth Factor-2 mRNA Binding Proteins (IGF2BPs/IMPs/VICKZs) bind RNA (R-HSA-428359)

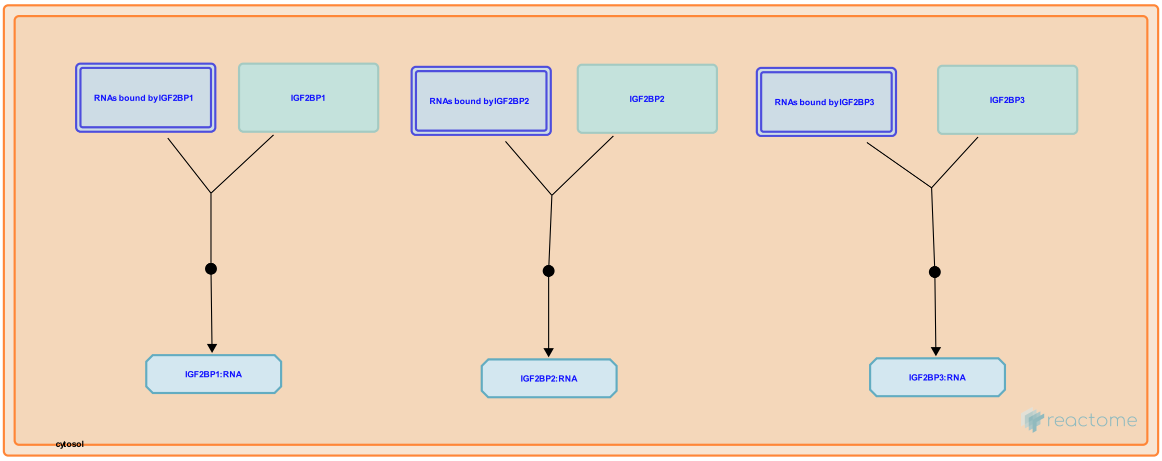

Cellular compartments: cytosol.

Insulin-like Growth Factor-2 mRNA Binding Proteins (IGF2BPs) bind specific sets of RNA and regulate their translation, stability, and subcellular localization. IGF2BP1, IGF2BP2, and IGF2BP3 bind about 8400 protein-coding transcripts. The target RNAs contain the sequence motif CAUH (where H is A, U, or, C) and binding of IGFBPs increases the stability of the target RNAs.

References

Nielsen J, Wewer UM, Christiansen J, Johnsen AH, Lykke-Andersen J & Nielsen FC (1999). A family of insulin-like growth factor II mRNA-binding proteins represses translation in late development. Mol Cell Biol, 19, 1262-70.

Nielsen J, Christiansen J & Nielsen FC (2001). A family of IGF-II mRNA binding proteins (IMP) involved in RNA trafficking. Scand J Clin Lab Invest Suppl, 234, 93-9.

Yisraeli JK (2005). VICKZ proteins: a multi-talented family of regulatory RNA-binding proteins. Biol Cell, 97, 87-96.

Rubinstein AM, Maizels Y, Oberman F, Rand K & Yisraeli JK (2007). VICKZ proteins mediate cell migration via their RNA binding activity. RNA, 13, 1558-69.

Wewer UM, Hansen TV, Christiansen J, Borup R, Jønson L, Vikesaa J & Nielsen FC (2006). RNA-binding IMPs promote cell adhesion and invadopodia formation. EMBO J, 25, 1456-68.

Edit history

Date	Action	Author
2009-07-05	Edited	May B
2009-07-05	Authored	May B
2009-07-05	Created	May B
2010-05-30	Reviewed	Chao JA, Singer RH
2023-10-12	Modified	Weiser JD

Interactors found in this pathway (1)

Input	UniProt Id	Interacts with	Input	UniProt Id	Interacts with
YBX1	P67809	Q9NZI8, Q9Y6M1			

21. Nitric oxide stimulates guanylate cyclase (R-HSA-392154)

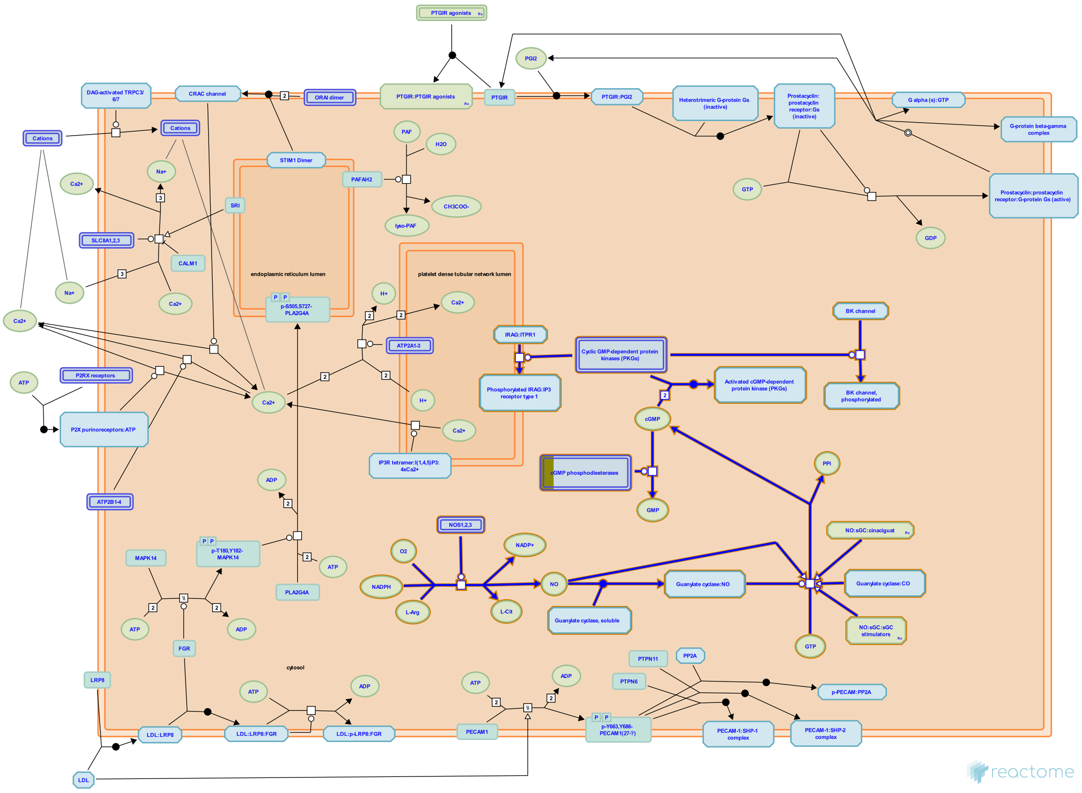

Cellular compartments: cytosol.

Nitric Oxide (NO) inhibits smooth muscle cell proliferation and migration, oxidation of low-density lipoproteins, and platelet aggregation and adhesion. It can stimulate vasodilatation of the endothelium, disaggregation of preformed platelet aggregates and inhibits activated platelet recruitment to the aggregate. NO is synthesized from L-arginine by a family of isoformic enzymes known as nitric oxide synthase (NOS). Three isoforms, namely endothelial, neuronal, and inducible NOS (eNOS, nNOS, and iNOS, respectively), have been identified. The eNOS isoform is found in the endothelium and platelets. NO regulation of cyclic guanosine-3,5-monophosphate (cGMP), via activation of soluble guanylate cyclase, is the principal mechanism of negative control over platelet activity. Defects in this control mechanism have been associated with platelet hyperaggregability and associated thrombosis.

References

Koesling D & Friebe A (2003). Regulation of nitric oxide-sensitive guanylyl cyclase. Circ Res, 93, 96-105.

Edit history

Date	Action	Author
2009-02-27	Created	Jupe S
2009-06-03	Authored	Akkerman JW
2010-06-07	Edited	Jupe S
2010-06-07	Reviewed	Kunapuli SP
2023-11-17	Modified	Wright A

1 submitted entities found in this pathway, mapping to 1 Reactome entities

Input	UniProt Id
PDE9A	O76083

22. Processing of Intronless Pre-mRNAs (R-HSA-77595)

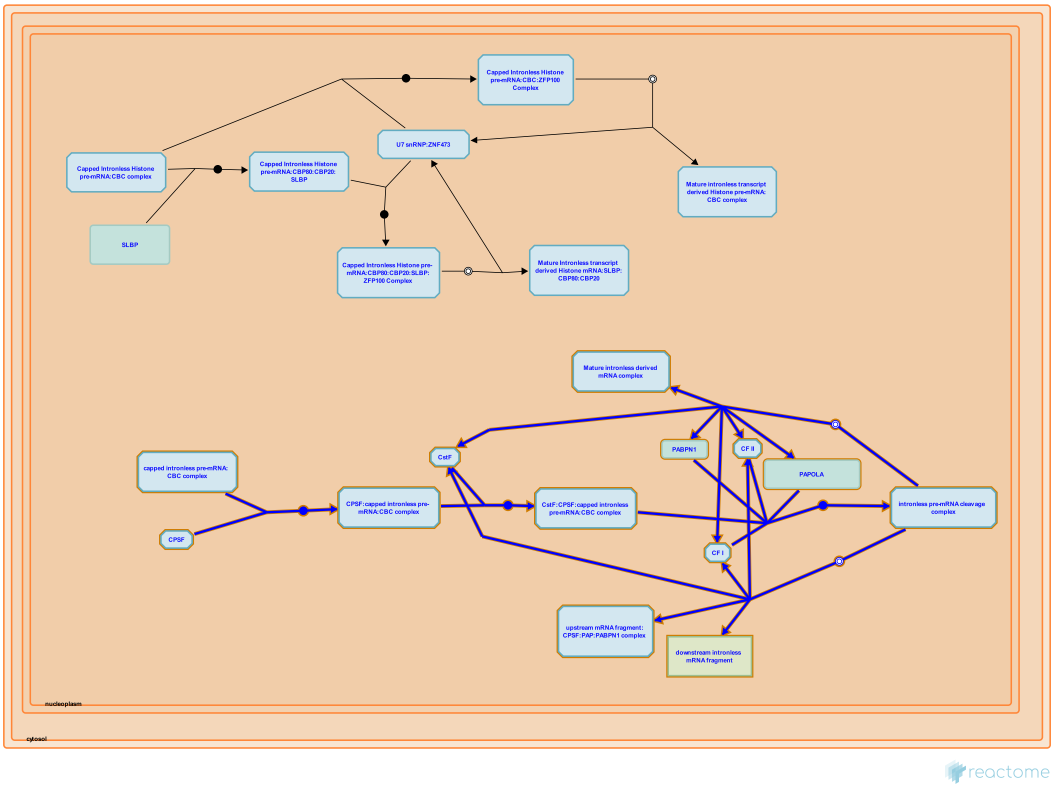

Cellular compartments: nucleoplasm.

The 3' ends of eukaryotic mRNAs are generated by posttranscriptional processing of an extended primary transcript. For almost all RNAs, 3' processing consists of two steps: The mRNA is first cleaved at a particular phosphodiester bond downstream of the coding sequence. The upstream fragment then receives a poly(A) tail of approximately 250 adenylate residues whereas the downstream fragment is degraded. The two partial reactions are coupled so that reaction intermediates are usually undetectable. While 3' processing can be studied as an isolated event in vitro, it appears to be connected to transcription, splicing and transcription termination in vivo.

References

Furger A, Dye MJ & Proudfoot NJ (2002). Integrating mRNA processing with transcription. Cell, 108, 501-12.

Edit history

Date	Action	Author
2003-06-05	Authored	Wahle E
2003-06-05	Created	Wahle E
2023-11-15	Edited	Gillespie ME
2023-11-17	Modified	Wright A

Interactors found in this pathway (1)

Input	UniProt Id	Interacts with	Input	UniProt Id	Interacts with
YBX1	P67809	Q86U42			

23. Receptor-type tyrosine-protein phosphatases (R-HSA-388844)

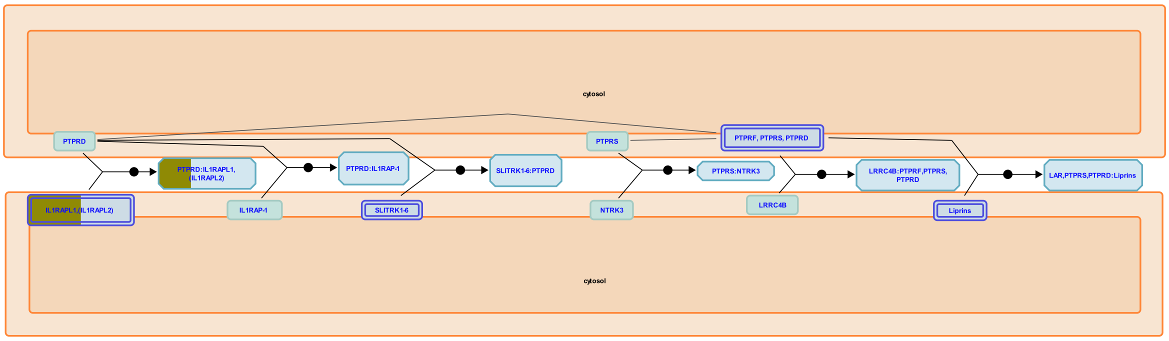

Cellular compartments: plasma membrane.

Like neurexins, Receptor-like protein tyrosine phosphatases (RPTPs) make trans-synaptic adhesion complexes with multiple postsynaptic binding partners to regulate synapse organization. The type IIa RPTPs include three members, Receptor-type tyrosine-protein phosphatase F (PTPRF) sometimes referred to as leukocyte common antigen-related (LAR), Receptor-type tyrosine-protein phosphatase sigma (PTPRS) and Receptor-type tyrosine-protein phosphatase delta (PTPRD). These proteins contain typical cell adhesion immunoglobulin-like (Ig) and fibronectin III (FNIII) domains, suggesting the involvement of RPTPs in cell-cell and cell-matrix interactions. To date, six different types of postsynaptic organizers for type-IIa RPTPs have been reported: interleukin-1 receptor accessory protein (IL1RAP, IL-1RAcP) (Yoshida et al. 2012), IL-1RAcP-like-1 (IL1RAPL1) (Yoshida et al. 2011), Neurotrophin receptor tyrosine kinase 3 (NTRK3, TrkC) (Takahashi et al. 2011), Leucine-rich repeat-containing protein 4B (LRRC4B, Netrin-G ligand-3, NGL-3) (Woo et al. 2009, Kwon et al. 2010), the Slit- and Trk-like (Slitrk) family proteins (Takahashi et al. 2012, Yim et al. 2013, Yamagata et al. 2015) and the liprins (Serra-Pagès et al. 1998, Dunah et al. 2005).

References

Craig AM & Takahashi H (2013). Protein tyrosine phosphatases PTPÎ´, PTPÏƒ, and LAR: presynaptic hubs for synapse organization. Trends Neurosci., 36, 522-34.

Edit history

Date	Action	Author
2008-12-16	Authored	Garapati P V
2008-12-16	Created	Garapati P V
2017-02-02	Edited	Jupe S
2017-02-03	Reviewed	Ko J
2023-11-16	Modified	Wright A

1 submitted entities found in this pathway, mapping to 1 Reactome entities

Input	UniProt Id
IL1RAPL2	Q9NP60

24. Processing of Capped Intronless Pre-mRNA (R-HSA-75067)

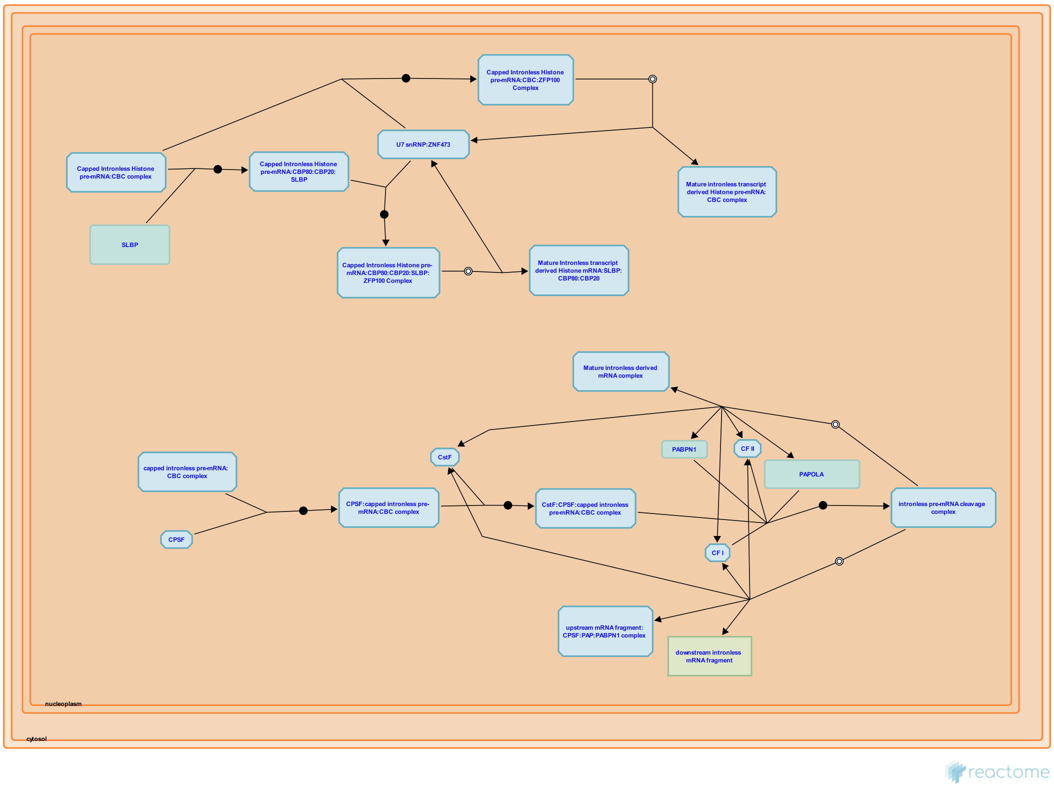

Cellular compartments: nucleoplasm.

Co-transcriptional pre-mRNA splicing is not obligatory. Pre-mRNA splicing begins co-transcriptionally and often continues post-transcriptionally. Human genes contain an average of nine introns per gene, which cannot serve as splicing substrates until both 5' and 3' ends of each intron are synthesized. Thus the time that it takes for pol II to synthesize each intron defines a minimal time and distance along the gene in which splicing factors can be recruited. The time that it takes for pol II to reach the end of the gene defines the maximal time in which splicing could occur co-transcriptionally. Thus, the kinetics of transcription can affect the kinetics of splicing.

There are two classes of intronless pre-mRNAs (mRNAs expressed from genes that lack introns). The first class encodes the replication dependent histone mRNAs. These mRNAs have unique 3' ends that do not have a polyA tail. The replication dependent histone mRNAs in all metazoans, as well as Chlamydomonas and Volvox fall into this class.

The second class of mRNAs end in polyA tails, which are formed by a mechanism similar to that which poly-adenylate pre-mRNAs containing introns. In the intronless genes there is a different method of replacing the 3' splice site that activates polyadenylation.

References

Gall JG (2001). Cajal bodies: the first 100 years. Annu Rev Cell Dev Biol, 16, 273-300.

Marzluff WF & Sanchez R (2002). The stem-loop binding protein is required for efficient translation of histone mRNA in vivo and in vitro. Mol Cell Biol, 22, 7093-104.

Marzluff WF & Dominski Z (1999). Formation of the 3' end of histone mRNA. Gene, 239, 1-14.

Marzluff WF, Maltais LJ, Woods KR, Jin J & Gongidi P (2002). The human and mouse replication-dependent histone genes. Genomics, 80, 487-98.

Frey MR & Matera AG (1995). Coiled bodies contain U7 small nuclear RNA and associate with specific DNA sequences in interphase human cells. Proc Natl Acad Sci U S A, 92, 5915-9.

Edit history

Date	Action	Author
2003-08-22	Authored	Marzluff WF
2004-06-23	Created	Wahle E, Marzluff WF
2023-11-15	Edited	Joshi-Tope G
2023-11-17	Modified	Wright A

Interactors found in this pathway (1)

Input	UniProt Id	Interacts with	Input	UniProt Id	Interacts with
YBX1	P67809	Q86U42			

25. Transcriptional activity of SMAD2/SMAD3:SMAD4 heterotrimer (R-HSA-2173793)

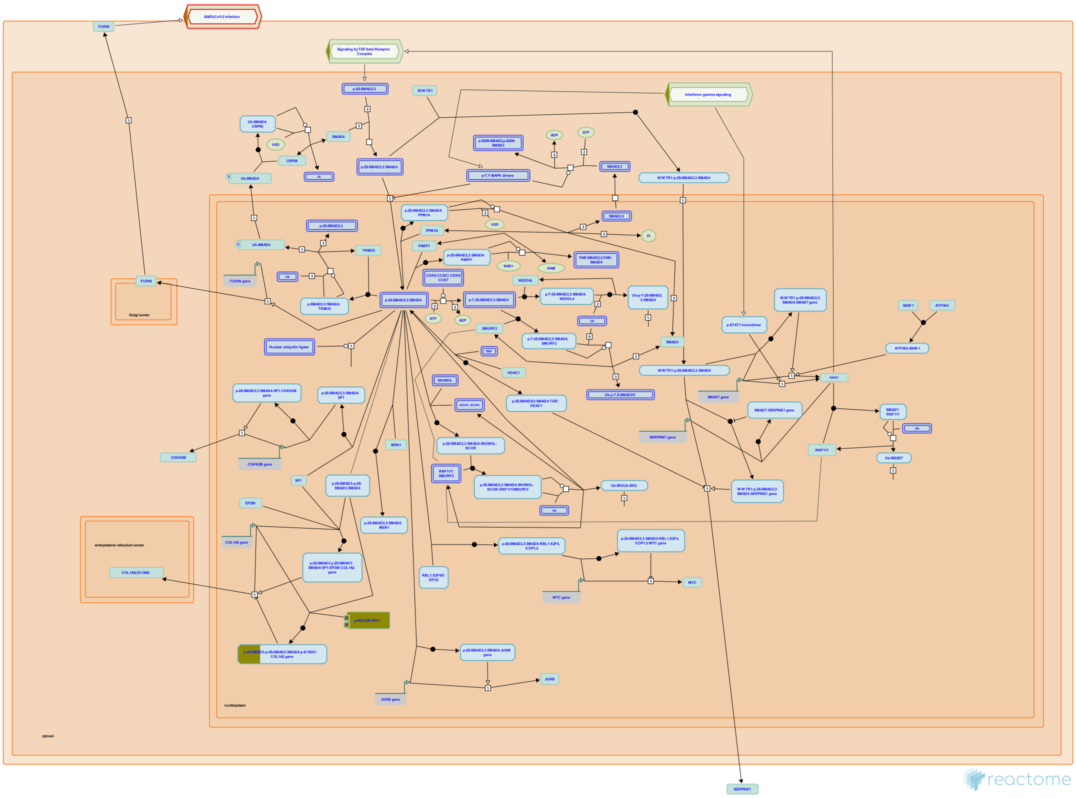

In the nucleus, SMAD2/3:SMAD4 heterotrimer complex acts as a transcriptional regulator. The activity of SMAD2/3 complex is regulated both positively and negatively by association with other transcription factors (Chen et al. 2002, Varelas et al. 2008, Stroschein et al. 1999, Wotton et al. 1999). In addition, the activity of SMAD2/3:SMAD4 complex can be inhibited by nuclear protein phosphatases and ubiquitin ligases (Lin et al. 2006, Dupont et al. 2009).

References

Massague J, Siegel PM, Chen CR & Kang Y (2002). E2F4/5 and p107 as Smad cofactors linking the TGFbeta receptor to c-myc repression. Cell, 110, 19-32.

Zhou Q, Luo K, Zhou S, Stroschein SL & Wang W (1999). Negative feedback regulation of TGF-beta signaling by the SnoN oncoprotein. Science, 286, 771-4.

Stinchfield MJ, Montagner M, Morsut L, Piccolo S, Inui M, Moro S, ... Cordenonsi M (2009). FAM/USP9x, a deubiquitinating enzyme essential for TGFbeta signaling, controls Smad4 monoubiquitination. Cell, 136, 123-35.

Massague J, Lee S, Lo RS & Wotton D (1999). A Smad transcriptional corepressor. Cell, 97, 29-39.

Wrana JL, Peerani R, Rao BM, Samavarchi-Tehrani P, Sakuma R, Zandstra PW, ... Dembowy J (2008). TAZ controls Smad nucleocytoplasmic shuttling and regulates human embryonic stem-cell self-renewal. Nat Cell Biol, 10, 837-48.

Edit history

Date	Action	Author
2012-04-02	Created	Orlic-Milacic M

Date	Action	Author
2012-04-05	Authored	Orlic-Milacic M
2012-04-10	Edited	Jassal B
2012-05-14	Reviewed	Huang T
2012-11-14	Reviewed	Chen YG
2022-05-02	Reviewed	Contreras O
2022-05-09	Edited	Orlic-Milacic M
2023-11-17	Modified	Wright A

1 submitted entities found in this pathway, mapping to 1 Reactome entities

Input	UniProt Id
YBX1	P67809

Interactors found in this pathway (2)

Input	UniProt Id	Interacts with	Input	UniProt Id	Interacts with
SAP30	O75446	Q13547	SMARCA5	O60264	P01106

6. Identifiers found

Below is a list of the input identifiers that have been found or mapped to an equivalent element in Reactome, classified by resource.

10 of the submitted entities were found, mapping to 12 Reactome entities

Input	UniProt Id	Input	UniProt Id	Input	UniProt Id
IL1RAPL2	Q9NP60	MICAL1	Q8TDZ2	PDE9A	O76083
RTN4R	Q9BZR6	SAP30	O75446, Q9HAJ7	SLC5A11	Q8WWX8
SLC7A10	Q9NS82	SMARCA5	O60264	UBA7	P41226
YBX1	P67809				

Interactors (8)

Input	UniProt Id	Interacts with	Input	UniProt Id	Interacts with
CEBPZ	Q03701	P49841	CTRL	P40313	O00501
PDE9A	O76083-2	P61964	RTN4R	Q99M75	Q96FE5
SAP30	O75446	Q92769, Q01658, Q13547	SMARCA5	O60264	Q9UIG0
UBA7	P41226	P05161	YBX1	P67809	Q9NR30

7. Identifiers not found

These 3 identifiers were not found neither mapped to any entity in Reactome.

FAP	FRMPD1	SCRG1
