# Supplementary material for: Differential circulating miRNA profiles identified miR-423-5p, miR-93-5p, and miR-4532 as potential biomarkers for cholangiocarcinoma diagnosis
Source: PeerJ. 2024 Dec 10;12:e18367. doi: 10.7717/peerj.18367 (PMC11639864; doi:10.7717/peerj.18367)
Supplement: Supplemental Information 3 — The up-regulated miRNA profile of CCA against HC was predicted by reactome. [file peerj-12-18367-s003.pdf]

reactome

Pathway Analysis Report

This report contains the pathway analysis results for the submitted sample ''. Analysis was performed against Reactome version 87 on 15/03/2024. The web link to these results is:

https://reactome.org/PathwayBrowser/#/ANALYSIS=MjAyNDAzMTQwMzAyMjJfMTU2NTY%3D

Please keep in mind that analysis results are temporarily stored on our server. The storage period depends on usage of the service but is at least 7 days. As a result, please note that this URL is only valid for a limited time period and it might have expired.

Table of Contents

1. Introduction
2. Properties
3. Genome-wide overview
4. Most significant pathways
5. Pathways details
6. Identifiers found
7. Identifiers not found

1. Introduction

Reactome is a curated database of pathways and reactions in human biology. Reactions can be considered as pathway 'steps'. Reactome defines a 'reaction' as any event in biology that changes the state of a biological molecule. Binding, activation, translocation, degradation and classical biochemical events involving a catalyst are all reactions. Information in the database is authored by expert biologists, entered and maintained by Reactome's team of curators and editorial staff. Reactome content frequently cross-references other resources e.g. NCBI, Ensembl, UniProt, KEGG (Gene and Compound), ChEBI, PubMed and GO. Orthologous reactions inferred from annotation for Homo sapiens are available for 14 non-human species including mouse, rat, chicken, puffer fish, worm, fly and yeast. Pathways are represented by simple diagrams following an SBGN-like format.

Reactome's annotated data describe reactions possible if all annotated proteins and small molecules were present and active simultaneously in a cell. By overlaying an experimental dataset on these annotations, a user can perform a pathway over-representation analysis. By overlaying quantitative expression data or time series, a user can visualize the extent of change in affected pathways and its progression. A binomial test is used to calculate the probability shown for each result, and the p-values are corrected for the multiple testing (Benjamini–Hochberg procedure) that arises from evaluating the submitted list of identifiers against every pathway.

To learn more about our Pathway Analysis, please have a look at our relevant publications:

Fabregat A, Sidiropoulos K, Garapati P, Gillespie M, Hausmann K, Haw R, … D'Eustachio P (2016). The reactome pathway knowledgebase. Nucleic Acids Research, 44(D1), D481–D487. https://doi.org/10.1093/nar/gkv1351.

Fabregat A, Sidiropoulos K, Viteri G, Forner O, Marin-Garcia P, Arnau V, … Hermjakob H (2017). Reactome pathway analysis: a high-performance in-memory approach. BMC Bioinformatics, 18.

2. Properties

- This is an **overrepresentation** analysis: A statistical (hypergeometric distribution) test that determines whether certain Reactome pathways are over-represented (enriched) in the submitted data. It answers the question 'Does my list contain more proteins for pathway X than would be expected by chance?' This test produces a probability score, which is corrected for false discovery rate using the Benjamani-Hochberg method.
- 71 out of 88 identifiers in the sample were found in Reactome, where 1275 pathways were hit by at least one of them.
- All non-human identifiers have been converted to their human equivalent.
- IntAct interactors were included to increase the analysis background. This greatly increases the size of Reactome pathways, which maximises the chances of matching your submitted identifiers to the expanded pathway, but will include interactors that have not undergone manual curation by Reactome and may include interactors that have no biological significance, or unexplained relevance.
- This report is filtered to show only results for species 'Homo sapiens' and resource 'all resources'.
- The unique ID for this analysis (token) is MjAyNDAzMTQwMzAyMjJfMTU2NTY%3D. This ID is valid for at least 7 days in Reactome's server. Use it to access Reactome services with your data.

3. Genome-wide overview

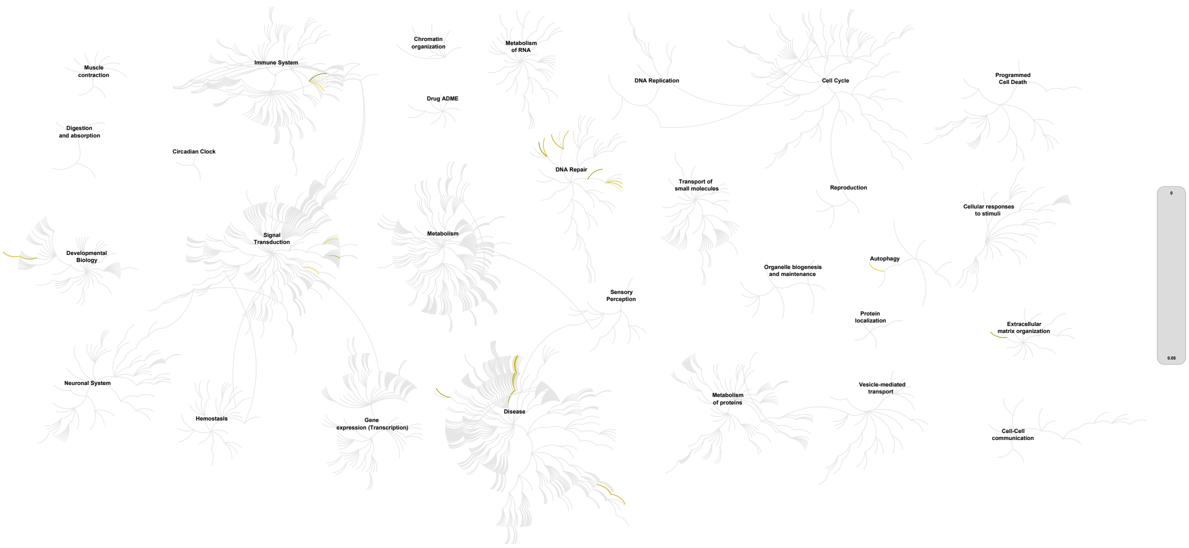

This figure shows a genome-wide overview of the results of your pathway analysis. Reactome pathways are arranged in a hierarchy. The center of each of the circular "bursts" is the root of one top-level pathway, for example "DNA Repair". Each step away from the center represents the next level lower in the pathway hierarchy. The color code denotes over-representation of that pathway in your input dataset. Light grey signifies pathways which are not significantly over-represented.

4. Most significant pathways

The following table shows the 25 most relevant pathways sorted by p-value.

Pathway name	Entities				Reactions	
	found	ratio	p-value	FDR*	found	ratio
TGFBR1 KD Mutants in Cancer	2 / 6	2.62e-04	9.68e-04	0.495	1 / 1	6.79e-05
Loss of Function of TGFBR1 in Cancer	2 / 7	3.06e-04	0.001	0.495	2 / 2	1.36e-04
SMAD2/3 Phosphorylation Motif Mutants in Cancer	2 / 7	3.06e-04	0.001	0.495	1 / 1	6.79e-05
RHO GTPases Activate Rhotekin and Rhophilins	3 / 29	0.001	0.001	0.495	5 / 6	4.08e-04
TGF-beta receptor signaling in EMT (epithelial to mesenchymal transition)	3 / 32	0.001	0.002	0.523	6 / 6	4.08e-04
Interleukin-4 and Interleukin-13 signaling	9 / 363	0.016	0.006	0.678	6 / 47	0.003
Late endosomal microautophagy	4 / 99	0.004	0.007	0.678	3 / 3	2.04e-04
Translesion synthesis by POLI	2 / 22	9.60e-04	0.012	0.678	3 / 3	2.04e-04
Translesion synthesis by POLK	2 / 23	0.001	0.013	0.678	3 / 3	2.04e-04
Gap-filling DNA repair synthesis and ligation in TC-NER	3 / 66	0.003	0.014	0.678	2 / 2	1.36e-04
TGFBR2 MSI Frameshift Mutants in Cancer	1 / 2	8.73e-05	0.015	0.678	1 / 1	6.79e-05
Sema4D induced cell migration and growth-cone collapse	2 / 25	0.001	0.015	0.678	4 / 7	4.75e-04
Gap-filling DNA repair synthesis and ligation in GG-NER	2 / 27	0.001	0.018	0.678	2 / 2	1.36e-04
Sema4D in semaphorin signaling	2 / 31	0.001	0.023	0.678	5 / 13	8.83e-04
Membrane binding and targetting of GAG proteins	2 / 32	0.001	0.024	0.678	3 / 4	2.72e-04
Synthesis And Processing Of GAG, GAGPOL Polyproteins	2 / 33	0.001	0.026	0.678	3 / 5	3.40e-04
RHOC GTPase cycle	3 / 85	0.004	0.026	0.678	6 / 6	4.08e-04
Dual incision in TC-NER	3 / 86	0.004	0.027	0.678	7 / 7	4.75e-04
Loss of Function of TGFBR2 in Cancer	1 / 4	1.75e-04	0.029	0.678	2 / 2	1.36e-04
TGFBR1 LBD Mutants in Cancer	1 / 4	1.75e-04	0.029	0.678	1 / 1	6.79e-05
TGFBR2 Kinase Domain Mutants in Cancer	1 / 4	1.75e-04	0.029	0.678	1 / 1	6.79e-05
Molecules associated with elastic fibres	2 / 39	0.002	0.035	0.678	4 / 10	6.79e-04

Pathway name	Entities				Reactions	
	found	ratio	p-value	FDR*	found	ratio
Recognition of DNA damage by PCNA-containing replication complex	2 / 40	0.002	0.036	0.678	5 / 6	4.08e-04
Defective Inhibition of DNA Recombination at Telomere Due to DAXX Mutations	1 / 5	2.18e-04	0.037	0.678	1 / 1	6.79e-05
Loss of Function of SMAD2/3 in Cancer	2 / 41	0.002	0.038	0.678	1 / 2	1.36e-04

* False Discovery Rate

5. Pathways details

For every pathway of the most significant pathways, we present its diagram, as well as a short summary, its bibliography and the list of inputs found in it.

1. TGFBR1 KD Mutants in Cancer (R-HSA-3656532)

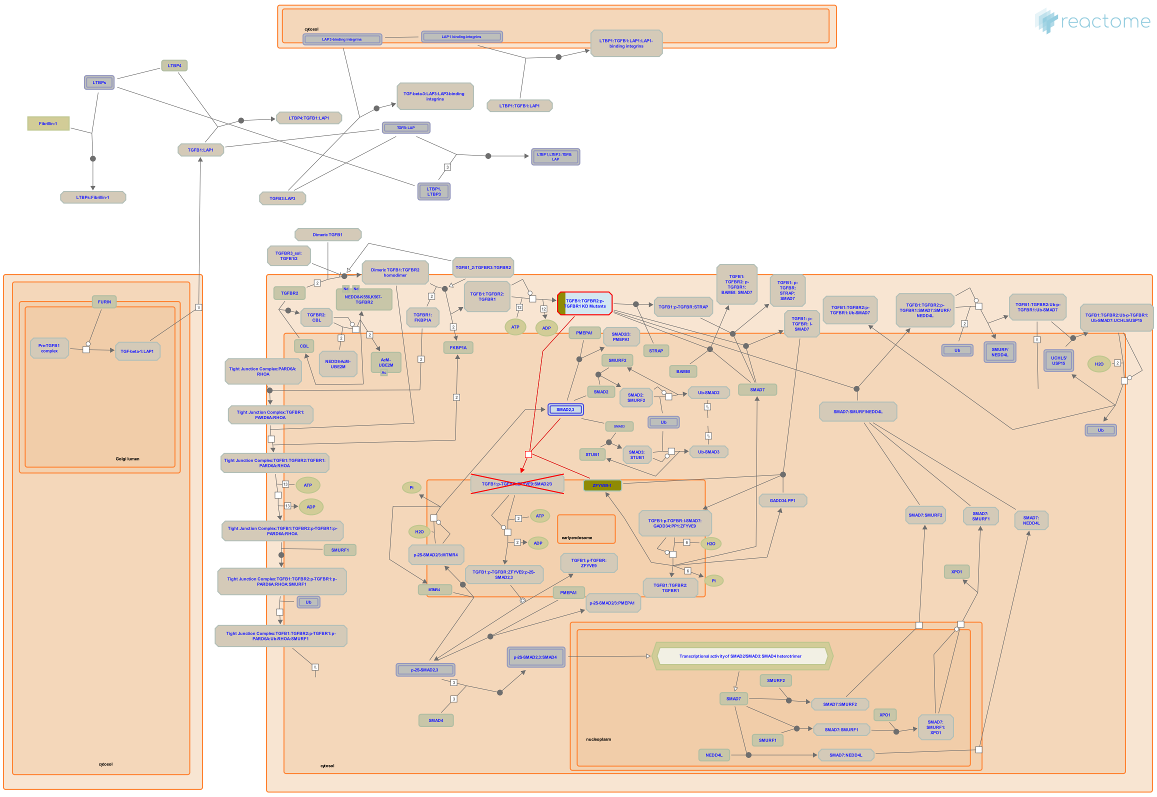

Diseases: cancer.

Mutations in the kinase domain (KD) of TGF-beta receptor 1 (TGFBR1) have been found in Ferguson-Smith tumor i.e. multiple self-healing squamous epithelioma - MSSE (Goudie et al. 2011), breast cancer (Chen et al. 1998), ovarian cancer (Chen et al. 2001) and head-and-neck cancer (Chen et al. 2001). KD mutations reported in MSSE are nonsense and frameshift mutations that cause premature termination of TGFBR1 translation, resulting in truncated receptors that lack substantial portions of the kinase domain, or cause nonsense-mediated decay of mutant transcripts. A splice site KD mutation c.806-2A>C is predicted to result in the skipping of exon 5 and the absence of KD amino acid residues 269-324 from the mutant receptor. The splice site mutant is expressed at the cell surface but unresponsive to TGF-beta stimulation (Goudie et al. 2004).

TGFBR1 KD mutations reported in breast, ovarian and head-and-neck cancer are missense mutations, and it appears that these mutant proteins are partially functional but that their catalytic activity or protein stability is decreased (Chen et al. 1998, Chen et al. 2001a and b). These mutants are not shown.

References

McNiff J, Leffell D, Chen T, Rimm DL, Wells RG, Yan W & Reiss M (2001). Novel inactivating mutations of transforming growth factor-beta type I receptor gene in head-and-neck cancer metastases. Int. J. Cancer, 93, 653-61.

Gerdes AM, Reversade B, Lee H, Ferguson-Smith MA, Whittaker S, Christie L, ... Verma C (2011). Multiple self-healing squamous epithelioma is caused by a disease-specific spectrum of mutations in TGFBR1. Nat. Genet., 43, 365-9.

Garrigue-Antar L, Chen T, Reiss M & Carter D (1998). Transforming growth factor beta type I receptor kinase mutant associated with metastatic breast cancer. Cancer Res., 58, 4805-10.

Colligan B, Chen T, Graff JR, Hurst B, Dehner B, Pemberton J, ... Triplett J (2001). Transforming growth factor-beta receptor type I gene is frequently mutated in ovarian carcinomas. Cancer Res., 61, 4679-82.

Edit history

Date	Action	Author
2013-06-05	Created	Orlic-Milacic M
2013-08-08	Edited	Orlic-Milacic M
2013-08-08	Reviewed	Meyer S, Akhurst RJ
2013-08-08	Authored	Meyer S, Akhurst RJ, Orlic-Milacic M
2023-11-28	Modified	Wright A

2 submitted entities found in this pathway, mapping to 2 Reactome entities

Input	UniProt Id	Input	UniProt Id
TGFB1	P01137	ZFYVE9	O95405-1

2. Loss of Function of TGFBR1 in Cancer (R-HSA-3656534)

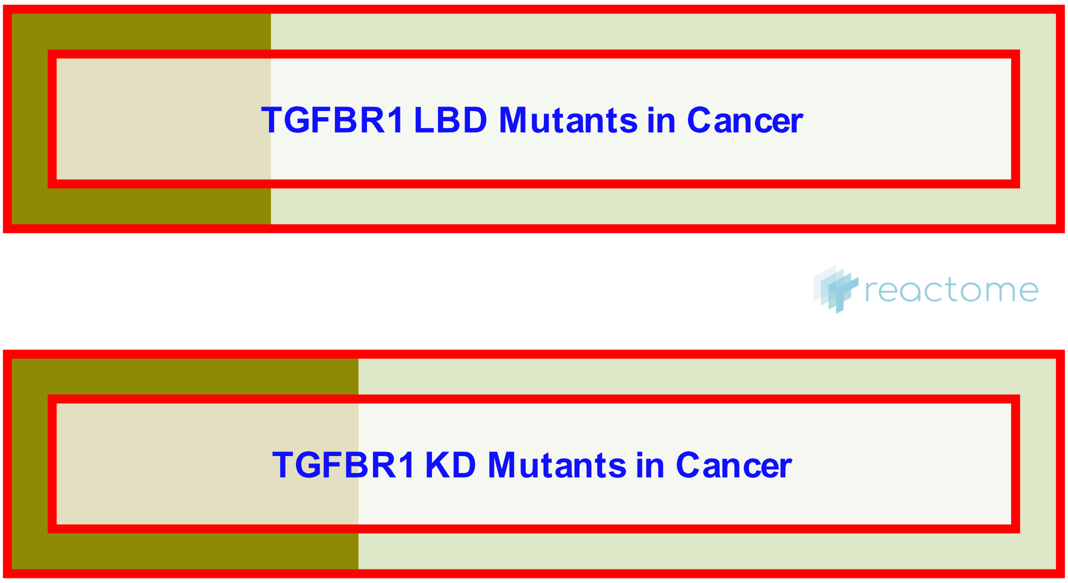

Diseases: cancer.

TGF-beta receptor 1 (TGFBR1) loss-of-function is a less frequent mechanism for inactivation of TGF-beta signaling in cancer compared to SMAD4 and TGFBR2 inactivation. Genomic deletion of TGFBR1 locus has been reported in pancreatic cancer (Goggins et al. 1998), biliary duct cancer (Goggins et al. 1998) and lymphoma (Schiemann et al. 1999), while loss-of-function mutations have been reported in breast (Chen et al. 1998) and ovarian cancer (Chen et al. 2001), metastatic head-and-neck cancer (Chen et al. 2001), and in Ferguson-Smith tumors (multiple self-healing squamous epithelioma - MSSE) (Goudie et al. 2011). Loss-of-function mutations mainly affect the ligand-binding extracellular domain of TGFBR1 and the kinase domain of TGFBR1 (Goudie et al. 2011). In the mouse model of colorectal cancer, Tgfbr1 haploinsufficiency cooperates with Apc haploinsufficiency in the development of intestinal tumors (Zeng et al. 2009).

References

McNiff J, Leffell D, Chen T, Rimm DL, Wells RG, Yan W & Reiss M (2001). Novel inactivating mutations of transforming growth factor-beta type I receptor gene in head-and-neck cancer metastases. Int. J. Cancer, 93, 653-61.

Gerdes AM, Reversade B, Lee H, Ferguson-Smith MA, Whittaker S, Christie L, ... Verma C (2011). Multiple self-healing squamous epithelioma is caused by a disease-specific spectrum of mutations in TGFBR1. Nat. Genet., 43, 365-9.

Garrigue-Antar L, Chen T, Reiss M & Carter D (1998). Transforming growth factor beta type I receptor kinase mutant associated with metastatic breast cancer. Cancer Res., 58, 4805-10.

Phukan S, Zeng Q, Pasche B, Yang GY, Liao J, Xu Y, ... Sadim M (2009). Tgfbr1 haploinsufficiency is a potent modifier of colorectal cancer development. Cancer Res., 69, 678-86.

Colligan B, Chen T, Graff JR, Hurst B, Dehner B, Pemberton J, ... Triplett J (2001). Transforming growth factor-beta receptor type I gene is frequently mutated in ovarian carcinomas. Cancer Res., 61, 4679-82.

Edit history

Date	Action	Author
2013-06-05	Created	Orlic-Milacic M
2013-08-08	Edited	Orlic-Milacic M
2013-08-08	Reviewed	Meyer S, Akhurst RJ
2013-08-08	Authored	Meyer S, Akhurst RJ, Orlic-Milacic M
2023-10-12	Modified	Weiser JD

2 submitted entities found in this pathway, mapping to 2 Reactome entities

Input	UniProt Id	Input	UniProt Id
TGFB1	P01137	ZFYVE9	O95405-1

3. SMAD2/3 Phosphorylation Motif Mutants in Cancer (R-HSA-3304356)

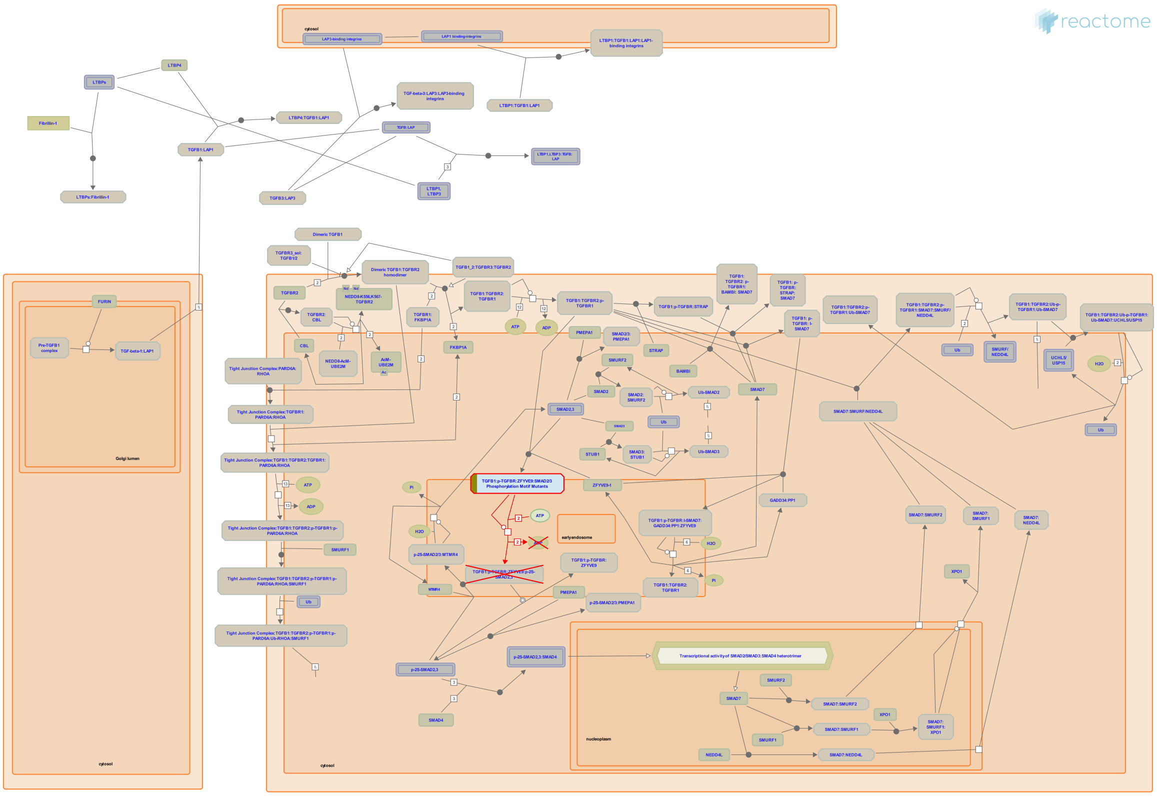

Diseases: cancer.

The conserved phosphorylation motif Ser-Ser-X-Ser at the C-terminus of SMAD2 and SMAD3 is subject to disruptive mutations in cancer. The last two serine residues in this conserved motif, namely Ser465 and Ser467 in SMAD2 and Ser423 and Ser425 in SMAD3, are phosphorylated by the activated TGF beta receptor complex (Macias Silva et al. 1996, Nakao et al. 1997). Once phosphorylated, SMAD2 and SMAD3 form transcriptionally active heterotrimers with SMAD4 (Chacko et al. 2001, Chacko et al. 2004). Phosphorylation motif mutants of SMAD2 and SMAD3 cannot be activated by the TGF-beta receptor complex either because serine residues are substituted with amino acid residues that cannot be phosphorylated or because the phosphorylation motif is deleted from the protein sequence or truncated (Fleming et al. 2013).

References

Shi G, De Caestecker M, Lin K, Chacko BM, Hayward LJ, Tiwari A, ... Lam S (2004). Structural basis of heteromeric smad protein assembly in TGF-beta signaling. Mol Cell, 15, 813-23.

Wrana JL, Attisano L, Abdollah S, Hoodless PA, Pirone R & Macias-Silva M (1996). MADR2 is a substrate of the TGFbeta receptor and its phosphorylation is required for nuclear accumulation and signaling. Cell, 87, 1215-24.

Correia JJ, Lam SS, de Caestecker MP, Qin B, Chacko BM & Lin K (2001). The L3 loop and C-terminal phosphorylation jointly define Smad protein trimerization. Nat. Struct. Biol., 8, 248-53.

Souchelnytskyi S, Engstrom U, ten Dijke P, Heldin CH, Wernstedt C & Tamaki K (1997). Phosphorylation of Ser465 and Ser467 in the C terminus of Smad2 mediates interaction with Smad4 and is required for transforming growth factor-beta signaling. J Biol Chem, 272, 28107-15.

Mouradov D, Jorissen RN, Jones IT, Tsui C, Palmieri M, Sieber OM, ... Zhao Q (2013). SMAD2, SMAD3 and SMAD4 mutations in colorectal cancer. Cancer Res., 73, 725-35.

Edit history

Date	Action	Author
2013-04-23	Created	Orlic-Milacic M
2013-05-03	Edited	Jassal B
2013-08-08	Edited	Orlic-Milacic M
2013-08-08	Reviewed	Meyer S, Akhurst RJ
2013-08-08	Authored	Meyer S, Akhurst RJ, Orlic-Milacic M
2023-11-28	Modified	Wright A

2 submitted entities found in this pathway, mapping to 2 Reactome entities

Input	UniProt Id	Input	UniProt Id
TGFB1	P01137	ZFYVE9	O95405-1

4. RHO GTPases Activate Rhotekin and Rhophilins (R-HSA-5666185)

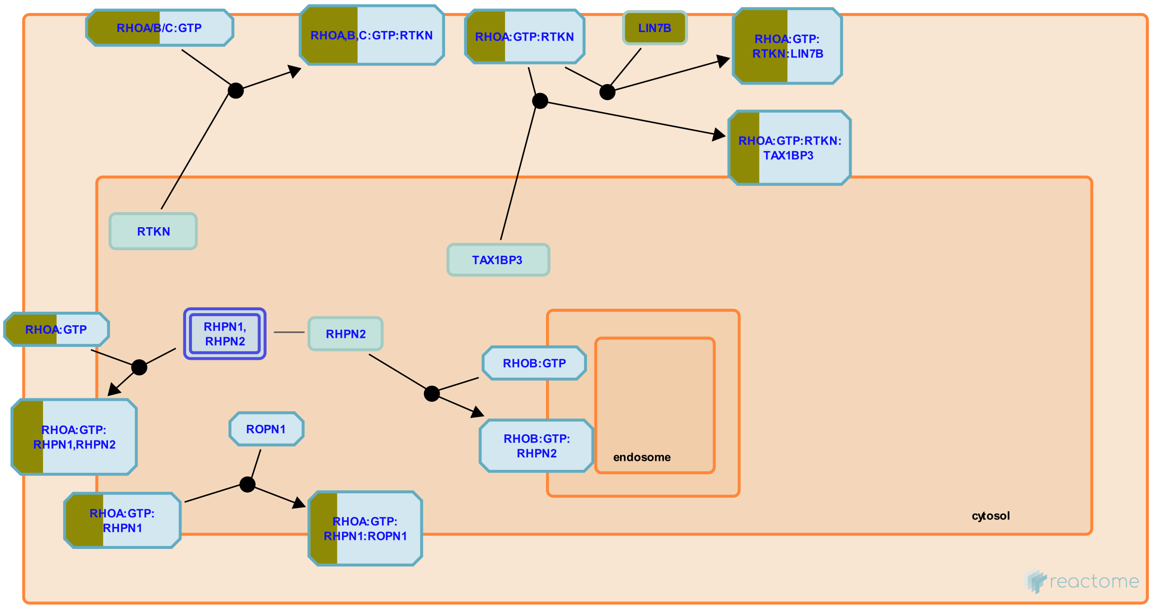

Rhotekin (RTKN) is a protein with an N-terminally located RHO GTPase binding domain, that shares a limited sequence homology with PKNs and rhophilins. RTKN binds to GTP-bound RHOA, RHOB and RHOC and can inhibit their GTPase activity (Reid et al. 1996, Fu et al. 2000), which can be corroborated by protein kinase D-mediated phosphorylation of RTKN (Pusapati et al. 2012). RTKN is implicated in the establishment of cell polarity (Sudo et al. 2006), septin organization (Ito et al. 2005, Sudo et al. 2007) and stimulation of SRF-mediated transcription (Reynaud et al. 2000). RTKN can have an anti-apoptotic effect that depends on the activation of NFKB (NF-kappaB) (Liu et al. 2004). RTKN2 (rhotekin-2) is another rhotekin exclusively expressed in lymphocytes (Collier et al. 2004). The function and the mechanism of action of RTKN2 are unknown.

Rhophillins include two family members - rhophilin-1 (RHNP1) and rhophilin-2 (RHPN2) with ~75% sequence identity. A RHO GTPase binding domain is located at the N-terminus of rhophilins, followed by a BRO1 domain (characteristic of proteins involved in protein kinase C signaling) and a C-terminal PDZ domain. RHOA:GTP binds both RHPN1 and RHPN2 and these interactions may be involved in organization of the actin cytoskeleton and/or cell motility (Watanabe et al. 1996, Fujita et al. 2000, Peck et al. 2002). RHOB:GTP recruits RHPN2 to endosomes which may be involved in the function of thyroid cells (Mircescu et al. 2002).

References

Bouker KB, Burbelo PD, Peck JW, Oberst M & Bowden E (2002). The RhoA-binding protein, rhophilin-2, regulates actin cytoskeleton organization. J. Biol. Chem., 277, 43924-32.

Morishita R, Ito H, Nagata K, Sudo K, Iwamoto I & Asano T (2007). SEPT9 sequence alternations causing hereditary neuralgic amyotrophy are associated with altered interactions with SEPT4/SEPT11 and resistance to Rho/Rhotekin-signaling. Hum. Mutat., 28, 1005-13.

Fabre S, Reynaud C & Jalinot P (2000). The PDZ protein TIP-1 interacts with the Rho effector rhotekin and is involved in Rho signaling to the serum response element. J. Biol. Chem., 275, 33962-8.

Madaule P, Fujisawa K, Narumiya S, Furuyashiki T, Watanabe G, Reid T, ... Morii N (1996). Rhotekin, a new putative target for Rho bearing homology to a serine/threonine kinase, PKN, and rhophilin in the rho-binding domain. J. Biol. Chem., 271, 13556-60.

Madaule P, Kakizuka A, Mukai H, Fujisawa K, Narumiya S, Watanabe G, ... Ono Y (1996). Protein kinase N (PKN) and PKN-related protein rhophilin as targets of small GTPase Rho. Science, 271, 645-8.

Edit history

Date	Action	Author
2014-10-24	Authored	Orlic-Milacic M
2014-12-26	Authored	Rivero Crespo F
2015-01-22	Created	Orlic-Milacic M
2015-02-02	Edited	Orlic-Milacic M
2023-11-16	Modified	Wright A

2 submitted entities found in this pathway, mapping to 3 Reactome entities

Input	UniProt Id	Input	UniProt Id
LIN7B	Q9HAP6	RHOC	P08134, P61586

5. TGF-beta receptor signaling in EMT (epithelial to mesenchymal transition) (R-HSA-2173791)

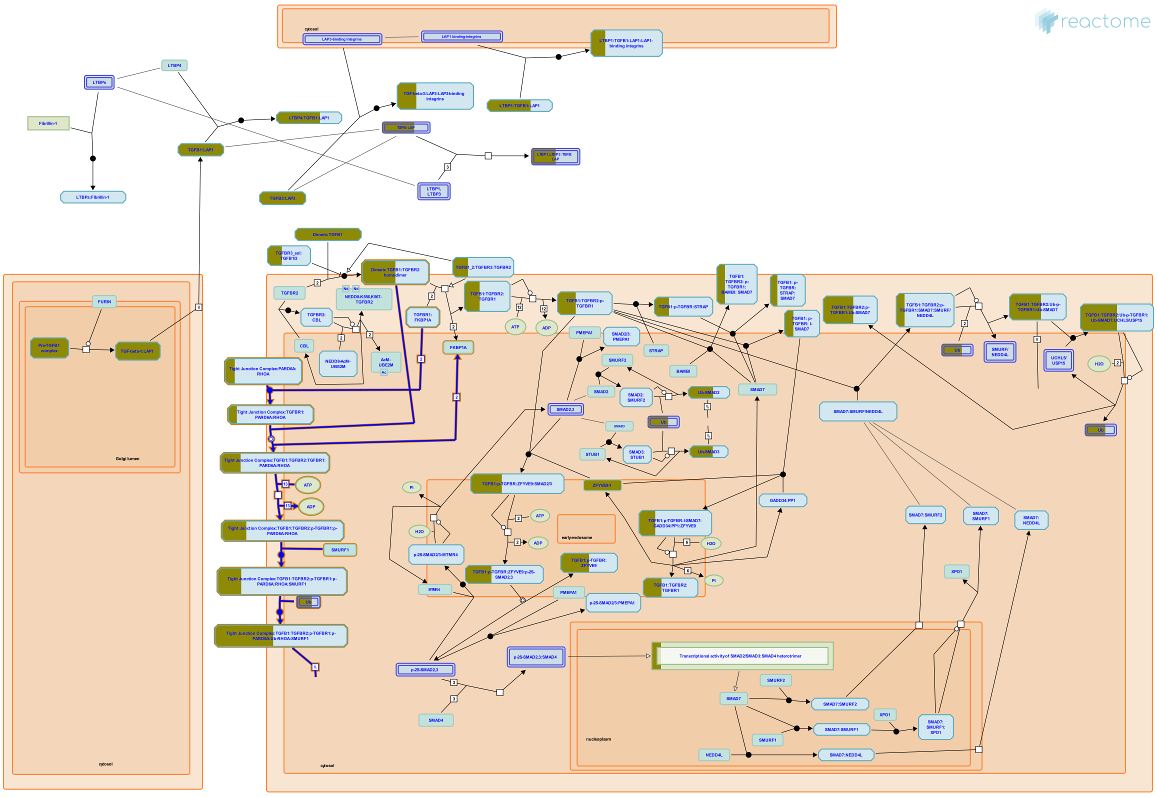

In normal cells and in the early stages of cancer development, signaling by TGF-beta plays a tumor suppressive role, as SMAD2/3:SMAD4-mediated transcription inhibits cell division by downregulating MYC oncogene transcription and stimulating transcription of CDKN2B tumor suppressor gene. In advanced cancers however, TGF-beta signaling promotes metastasis by stimulating epithelial to mesenchymal transition (EMT).

TGFBR1 is recruited to tight junctions by binding PARD6A, a component of tight junctions. After TGF-beta stimulation, activated TGFBR2 binds TGFBR1 at tight junctions, and phosphorylates both TGFBR1 and PARD6A. Phosphorylated PARD6A recruits SMURF1 to tight junctions. SMURF1 is able to ubiquitinate RHOA, a component of tight junctions needed for tight junction maintenance, leading to disassembly of tight junctions, an important step in EMT (Wang et al. 2003, Ozdamar et al. 2005).

References

Barrios-Rodiles M, Zhang Y, Wrana JL, Wang HR, Bose R & Ozdamar B (2005). Regulation of the polarity protein Par6 by TGFbeta receptors controls epithelial cell plasticity. Science, 307, 1603-9.

Zhang Y, Thomsen GH, Wrana JL, Wang HR, Ogunjimi AA, Ozdamar B & Alexandrova E (2003). Regulation of cell polarity and protrusion formation by targeting RhoA for degradation. Science, 302, 1775-9.

Edit history

Date	Action	Author
2012-04-02	Created	Orlic-Milacic M

Date	Action	Author
2012-04-05	Authored	Orlic-Milacic M
2012-04-10	Edited	Jassal B
2012-05-14	Reviewed	Huang T
2012-11-14	Reviewed	Chen YG
2023-11-28	Modified	Wright A

3 submitted entities found in this pathway, mapping to 3 Reactome entities

Input	UniProt Id	Input	UniProt Id	Input	UniProt Id
RHOC	P61586	TGFB1	P01137	UBC	P0CG48

6. Interleukin-4 and Interleukin-13 signaling (R-HSA-6785807)

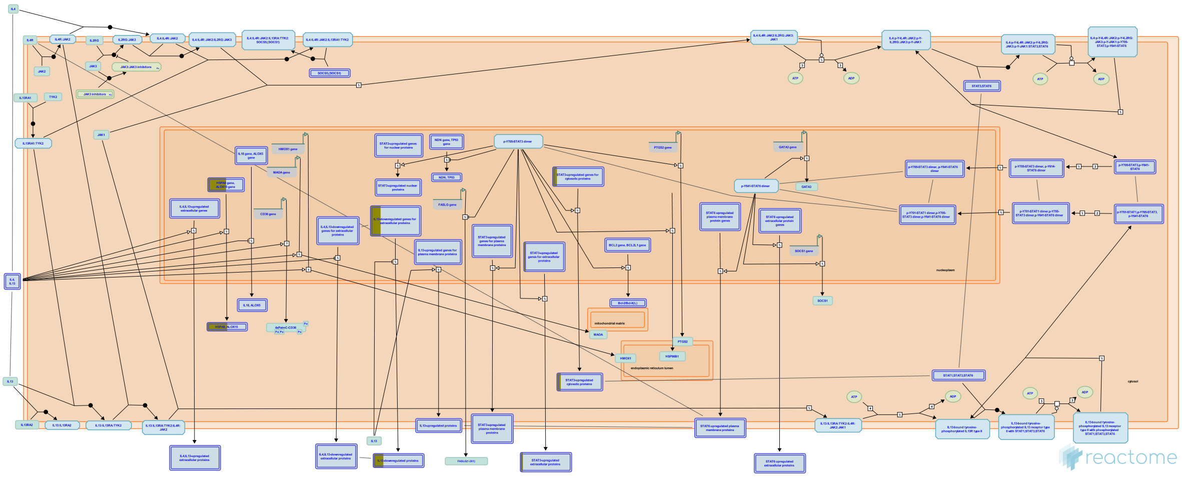

Interleukin-4 (IL4) is a principal regulatory cytokine during the immune response, crucially important in allergy and asthma (Nelms et al. 1999). When resting T cells are antigen-activated and expand in response to Interleukin-2 (IL2), they can differentiate as Type 1 (Th1) or Type 2 (Th2) T helper cells. The outcome is influenced by IL4. Th2 cells secrete IL4, which both stimulates Th2 in an autocrine fashion and acts as a potent B cell growth factor to promote humoral immunity (Nelms et al. 1999).

Interleukin-13 (IL13) is an immunoregulatory cytokine secreted predominantly by activated Th2 cells. It is a key mediator in the pathogenesis of allergic inflammation. IL13 shares many functional properties with IL4, stemming from the fact that they share a common receptor subunit. IL13 receptors are expressed on human B cells, basophils, eosinophils, mast cells, endothelial cells, fibroblasts, monocytes, macrophages, respiratory epithelial cells, and smooth muscle cells, but unlike IL4, not T cells. Thus IL13 does not appear to be important in the initial differentiation of CD4 T cells into Th2 cells, rather it is important in the effector phase of allergic inflammation (Hershey et al. 2003).

IL4 and IL13 induce “alternative activation” of macrophages, inducing an anti-inflammatory phenotype by signaling through IL4R alpha in a STAT6 dependent manner. This signaling plays an important role in the Th2 response, mediating anti-parasitic effects and aiding wound healing (Gordon & Martinez 2010, Loke et al. 2002)

There are two types of IL4 receptor complex (Andrews et al. 2006). Type I IL4R (IL4R1) is predominantly expressed on the surface of hematopoietic cells and consists of IL4R and IL2RG, the common gamma chain. Type II IL4R (IL4R2) is predominantly expressed on the surface of nonhematopoietic cells, it consists of IL4R and IL13RA1 and is also the type II receptor for IL13. (Obiri et al. 1995, Aman et al. 1996, Hilton et al. 1996, Miloux et al. 1997, Zhang et al. 1997). The second receptor for IL13 consists of IL4R and Interleukin-13 receptor alpha 2 (IL13RA2), sometimes called Interleukin-13 binding protein (IL13BP). It has a high affinity receptor for IL13 (Kd = 250 pmol/L) but is not sufficient to render cells responsive to IL13, even in the presence of IL4R (Donaldson et al. 1998). It is reported to exist in soluble form (Zhang et al. 1997) and when overexpressed reduces JAK-STAT signaling (Kawakami et al. 2001). It's function may be to prevent IL13 signalling via the functional IL4R:IL13RA1 receptor. IL13RA2 is overexpressed and enhances cell invasion in some human cancers (Joshi & Puri 2012).

The first step in the formation of IL4R1 (IL4:IL4R:IL2RB) is the binding of IL4 with IL4R (Hoffman et al. 1995, Shen et al. 1996, Hage et al. 1999). This is also the first step in formation of IL4R2 (IL4:IL4R:IL13RA1). After the initial binding of IL4 and IL4R, IL2RB binds (LaPorte et al. 2008), to form IL4R1. Alternatively, IL13RA1 binds, forming IL4R2. In contrast, the type II IL13 complex (IL13R2) forms with IL13 first binding to IL13RA1 followed by recruitment of IL4R (Wang et al. 2009).

Crystal structures of the IL4:IL4R:IL2RG, IL4:IL4R:IL13RA1 and IL13:IL4R:IL13RA1 complexes have been determined (LaPorte et al. 2008). Consistent with these structures, in monocytes IL4R is tyrosine phosphorylated in response to both IL4 and IL13 (Roy et al. 2002, Gordon & Martinez 2010) while IL13RA1 phosphorylation is induced only by IL13 (Roy et al. 2002, LaPorte et al. 2008) and IL2RG phosphorylation is induced only by IL4 (Roy et al. 2002).

Both IL4 receptor complexes signal through Jak/STAT cascades. IL4R is constitutively-associated with JAK2 (Roy et al. 2002) and associates with JAK1 following binding of IL4 (Yin et al. 1994) or IL13 (Roy et al. 2002). IL2RG constitutively associates with JAK3 (Boussiotis et al. 1994, Russell et al. 1994). IL13RA1 constitutively associates with TYK2 (Umeshita-Suyama et al. 2000, Roy et al. 2002, LaPorte et al. 2008, Bhattacharjee et al. 2013).

IL4 binding to IL4R1 leads to phosphorylation of JAK1 (but not JAK2) and STAT6 activation (Takeda et al. 1994, Ratthe et al. 2007, Bhattacharjee et al. 2013).

IL13 binding increases activating tyrosine-99 phosphorylation of IL13RA1 but not that of IL2RG. IL4 binding to IL2RG leads to its tyrosine phosphorylation (Roy et al. 2002). IL13 binding to IL4R2 leads to TYK2 and JAK2 (but not JAK1) phosphorylation (Roy & Cathcart 1998, Roy et al. 2002).

Phosphorylated TYK2 binds and phosphorylates STAT6 and possibly STAT1 (Bhattacharjee et al. 2013).

A second mechanism of signal transduction activated by IL4 and IL13 leads to the insulin receptor substrate (IRS) family (Kelly-Welch et al. 2003). IL4R1 associates with insulin receptor substrate 2 and activates the PI3K/Akt and Ras/MEK/Erk pathways involved in cell proliferation, survival and translational control. IL4R2 does not associate with insulin receptor substrate 2 and consequently the PI3K/Akt and Ras/MEK/Erk pathways are not activated (Busch-Dienstfertig & González-Rodríguez 2013).

References

Ryan JJ, Nelms K, Paul WE, Zamorano J & Keegan AD (1999). The IL-4 receptor: signaling mechanisms and biologic functions. Annu. Rev. Immunol., 17, 701-38.

Hershey GK (2003). IL-13 receptors and signaling pathways: an evolving web. J. Allergy Clin. Immunol., 111, 677-90; quiz 691.

Edit history

Date	Action	Author
2015-07-01	Authored	Jupe S
2015-07-01	Created	Jupe S
2016-09-02	Edited	Jupe S
2016-09-02	Reviewed	Leibovich SJ

Date	Action	Author
2023-11-28	Modified	Wright A

4 submitted entities found in this pathway, mapping to 8 Reactome entities

Input	UniProt Id
HSPA8	P11142
TGFB1	P01137

Input	UniProt Id
IL12A	P29459
VIM	P08670

Input	Ensembl Id
HSPA8	ENSG00000109971
TGFB1	ENSG00000105329

Input	Ensembl Id
IL12A	ENSG00000168811
VIM	ENSG00000026025

Interactors found in this pathway (1)

Input	UniProt Id	Interacts with
TCP1	P17987	P52333

Input	UniProt Id	Interacts with

7. Late endosomal microautophagy (R-HSA-9615710)

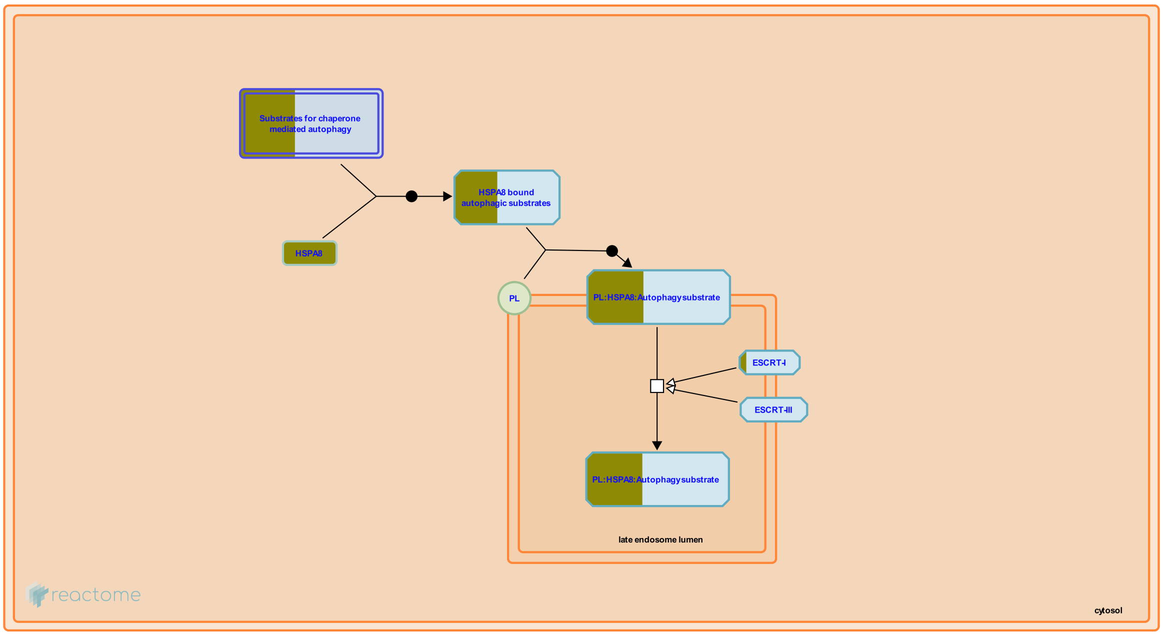

Cellular compartments: phagocytic vesicle, cytosol.

Microautophagy (MI) is a non-selective autophagic pathway that involves internalisation of cytosolic cargo through invaginations of the lysosomal membrane. MI can be induced by nitrogen starvation and complements other related self-eating processes such as Macroautophagy (MA) and Chaperone Mediated Autophagy (CMA). MI can degrade cell organelles and bulk cytosolic proteins directly via the lysosome and late endosome. MI can also target substrates with KFERQ motifs with the help of HSPA8 (Li W W et al. 2012).

References

Li WW, Bao JK & Li J (2012). Microautophagy: lesser-known self-eating. Cell. Mol. Life Sci., 69, 1125-36.

Edit history

Date	Action	Author
2018-08-08	Created	Varusai TM
2019-02-21	Authored	Varusai TM
2019-02-22	Reviewed	Metzakopian E
2019-10-31	Revised	Varusai TM
2019-11-08	Edited	Varusai TM
2023-11-28	Modified	Wright A

4 submitted entities found in this pathway, mapping to 4 Reactome entities

Input	UniProt Id	Input	UniProt Id
HSPA8	P11142	TSG101	Q99816
UBC	P0CG48	VIM	P08670

8. Translesion synthesis by POLI (R-HSA-5656121)

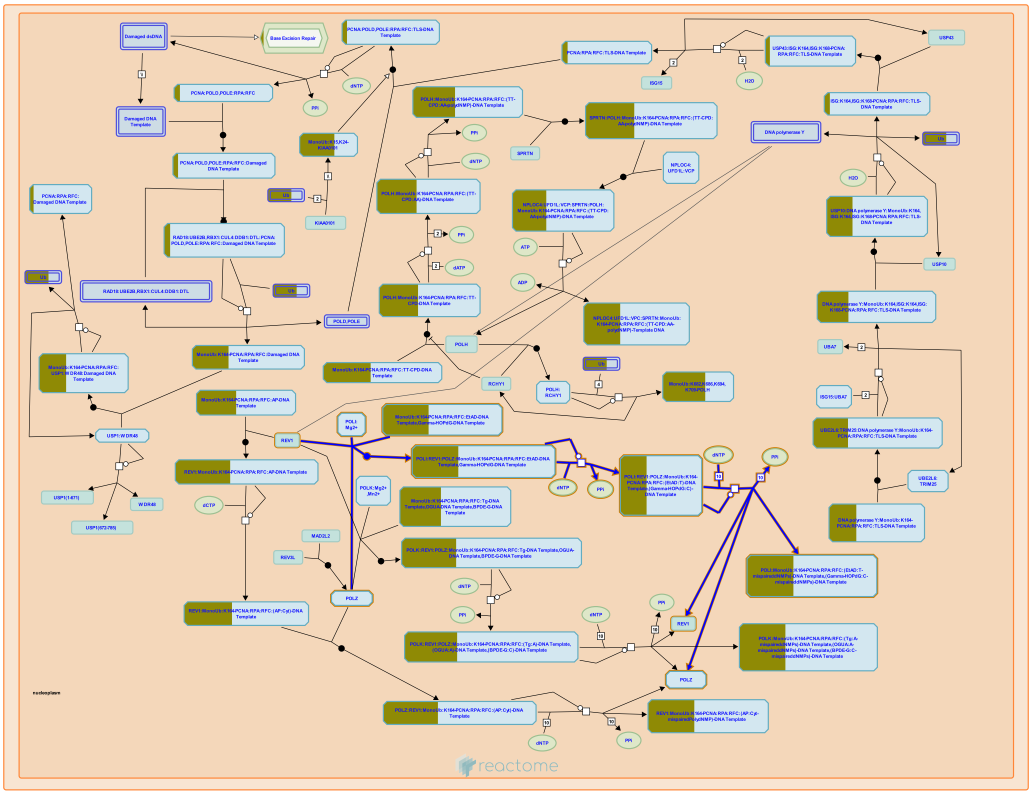

Cellular compartments: nucleoplasm.

DNA polymerase iota (POLI) is a Y family DNA polymerase with an active site that favours Hoogsteen base pairing instead of Watson-Crick base pairing. POLI-mediated Hoogsteen base pairing and rotation of template purines from anti to syn conformation serves as a mechanism to displace adducts on template G or template A that interfere with DNA replication, or to allow base pairing of damaged purines with a disrupted Watson-Crick edge but an intact Hoogsteen edge (Nair et al. 2004, Nair et al. 2006).

POLI is recruited to DNA damage sites through its interaction with PCNA and REV1. POLI contains a PIP box and two UBMs (ubiquitin binding motifs) that are responsible for POLI binding to monoubiquitinated PCNA (MonoUb:K164-PCNA) (Bienko et al. 2005, Haracska et al. 2005, Bomar et al. 2010). The interaction between POLI and the C-terminus of REV1 is evolutionarily conserved (Kosarek et al. 2003, Guo et al. 2003, Ohashi et al. 2004).

After it incorporates a dNMP opposite to damaged template base, POLI is unable to efficiently elongate the DNA strand further. The elongation step is performed by the polymerase zeta complex (POLZ), composed of REV3L and MAD2L2 subunits (Johnson et al. 2000). The involvement of REV1 and POLZ in POLI-mediated translesion DNA synthesis (TLS) suggests that POLI forms a quaternary complex with REV1 and POLZ, as shown for POLK and proposed for other Y family DNA polymerases (Xie et al. 2012).

References

Johnson RE, Haracska L, Washington MT, Prakash L & Prakash S (2000). Eukaryotic polymerases iota and zeta act sequentially to bypass DNA lesions. Nature, 406, 1015-9.

Murakumo Y, Akagi J, Kanjo N, Hanaoka F, Ohmori H, Ohashi E & Masutani C (2004). Interaction of hREV1 with three human Y-family DNA polymerases. Genes Cells, 9, 523-31.

Masuda Y, Friedberg EC, Zhou J, Fischhaber PL, Guo C, Kamiya K, ... Luk-Paszyc MJ (2003). Mouse Rev1 protein interacts with multiple DNA polymerases involved in translesion DNA synthesis. EMBO J., 22, 6621-30.

Xu M, Yang X, Xie W & Jiang T (2012). Structural insights into the assembly of human translesion polymerase complexes. Protein Cell, 3, 864-74.

Johnson RE, Haracska L, Hurwitz J, Acharya N, Prakash L, Unk I & Prakash S (2005). A single domain in human DNA polymerase iota mediates interaction with PCNA: implications for translesion DNA synthesis. Mol. Cell. Biol., 25, 1183-90.

Edit history

Date	Action	Author
2014-12-10	Created	Orlic-Milacic M
2014-12-11	Edited	Orlic-Milacic M
2014-12-11	Authored	Orlic-Milacic M
2015-01-07	Reviewed	Borowiec JA
2023-11-28	Modified	Wright A

2 submitted entities found in this pathway, mapping to 2 Reactome entities

Input	UniProt Id	Input	UniProt Id
RPA2	P15927	UBC	P0CG48

9. Translesion synthesis by POLK (R-HSA-5655862)

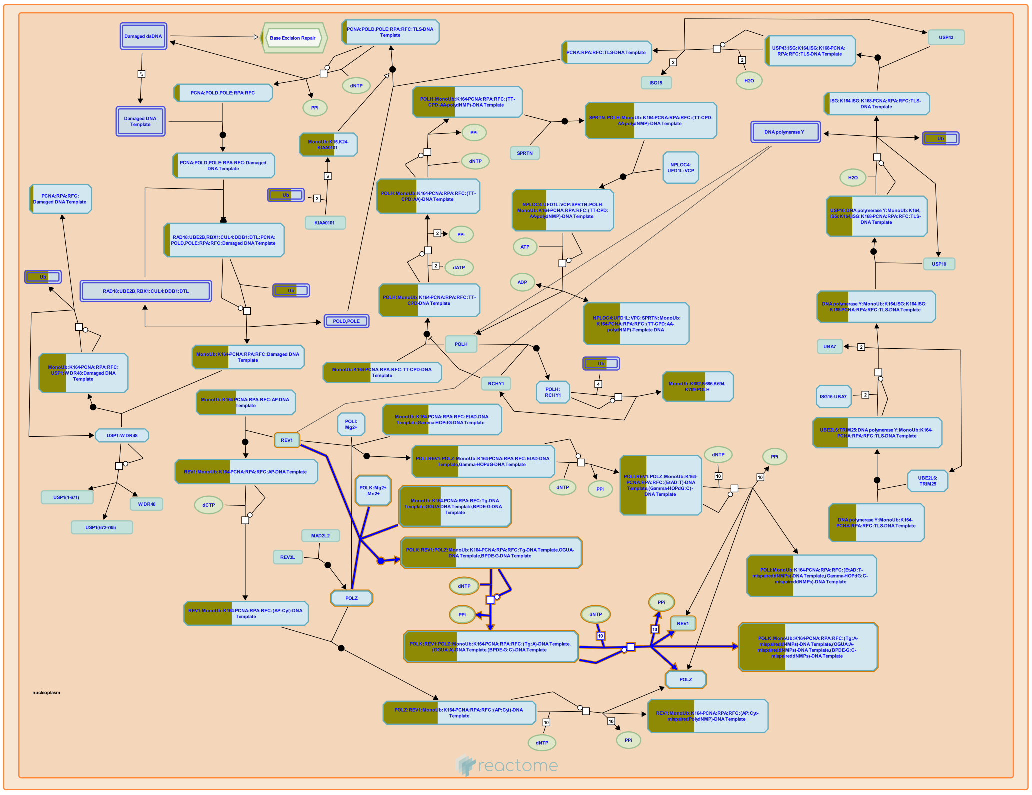

Cellular compartments: nucleoplasm.

DNA polymerase kappa (POLK) is a Y family DNA polymerase that is most efficient in translesion DNA synthesis (TLS) across oxidation derivatives of DNA bases, such as thymine glycol (Tg) and 8-oxoguanine (OGUA), as well as bulky DNA adducts, such as benzo(a)pyrene diol epoxide guanine adduct (BPDE-G) (Zhang et al. 2000, Fischhaber et al.2002, Avkin et al. 2004, Vasquez-Del Carpio et al. 2009, Yoon et al. 2010, Lior-Hoffmann et al. 2012, Christov et al. 2012, Yoon et al. 2014). POLK carries out TLS by forming a quaternary complex with REV1 and POLZ (REV3L:MAD2L2) at DNA damage sites, where POLK simultaneously binds REV1 and monoubiquitinated PCNA (Ohashi et al. 2009, Haracska, Unk et al. 2002, Bi et al. 2006). POLK and POLZ cooperate in the elongation of nucleotides inserted opposite to lesioned bases by POLK. Similarly to POLZ, POLK has low processivity and is error-prone (Ohashi et al. 2000, Haracska, Prakash et al. 2002, Yoon et al. 2010).

References

Prakash L, Roy Choudhury J, Yoon JH, Park J & Prakash S (2014). A role for DNA polymerase ? in promoting replication through oxidative DNA lesion, thymine glycol, in human cells. J. Biol. Chem., 289, 13177-85.

Geacintov NE, Zhang Y, Yuan F, Wang M, Taylor JS, Wang Z, ... Wu X (2000). Error-free and error-prone lesion bypass by human DNA polymerase kappa in vitro. Nucleic Acids Res., 28, 4138-46.

Gerlach VL, Feaver WJ, Friedberg EC, Kunkel TA, Matsuda T, Ohashi E, ... Ohmori H (2000). Fidelity and processivity of DNA synthesis by DNA polymerase kappa, the product of the human DINB1 gene. J. Biol. Chem., 275, 39678-84.

Hatahet Z, Friedberg EC, Gerlach VL, Fischhaber PL, Feaver WJ & Wallace SS (2002). Human DNA polymerase kappa bypasses and extends beyond thymine glycols during translesion synthesis in vitro, preferentially incorporating correct nucleotides. J. Biol. Chem., 277, 37604-11.

Rizzo CJ, Christov PP, Lloyd RS, Wood RD, Takata K, Choi JY, ... Yamanaka K (2012). Replication of the 2,6-diamino-4-hydroxy-N(5)-(methyl)-formamidopyrimidine (MeFapy-dGuo) adduct by eukaryotic DNA polymerases. Chem. Res. Toxicol., 25, 1652-61.

Edit history

Date	Action	Author
2014-12-09	Created	Orlic-Milacic M
2014-12-11	Edited	Orlic-Milacic M
2014-12-11	Authored	Orlic-Milacic M
2015-01-07	Reviewed	Borowiec JA
2023-11-28	Modified	Wright A

2 submitted entities found in this pathway, mapping to 2 Reactome entities

Input	UniProt Id	Input	UniProt Id
RPA2	P15927	UBC	P0CG48

10. Gap-filling DNA repair synthesis and ligation in TC-NER (R-HSA-6782210)

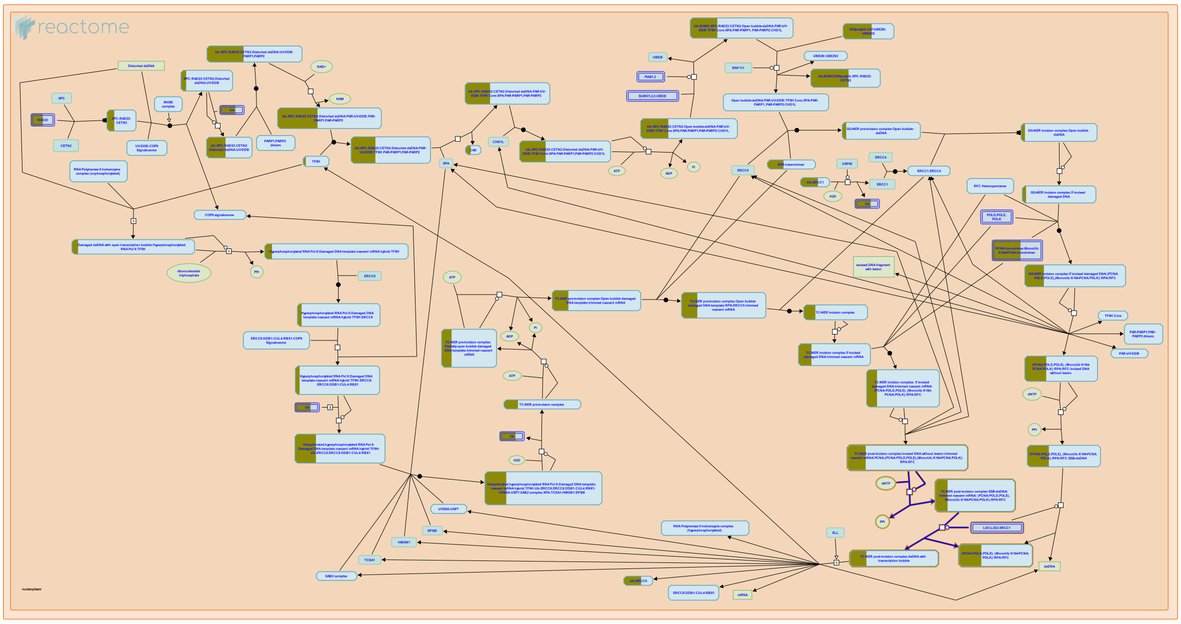

Cellular compartments: nucleoplasm.

In transcription-coupled nucleotide excision repair (TC-NER), similar to global genome nucleotide excision repair (GG-NER), DNA polymerases delta or epsilon, or the Y family DNA polymerase kappa, fill in the single stranded gap that remains after dual incision. DNA ligases LIG1 or LIG3, the latter in complex with XRCC1, subsequently seal the single stranded nick by ligating the 3' end of the newly synthesized patch with the 5' end of incised DNA (Moser et al. 2007, Staresincic et al. 2009, Ogi et al. 2010).

References

Limsirichaikul S, Takenaka K, Yamashita S, Cloney R, Lehmann AR, Miki Y, ... Nakazawa Y (2010). Three DNA polymerases, recruited by different mechanisms, carry out NER repair synthesis in human cells. Mol. Cell, 37, 714-27.

Wijgers N, Staresincic L, Schärer OD, Gourdin AM, Fagbemi AF, Enzlin JH, ... Vermeulen W (2009). Coordination of dual incision and repair synthesis in human nucleotide excision repair. EMBO J., 28, 1111-20.

Fousteri M, Mullenders LH, Giakzidis I, Moser J, Caldecott K & Kool H (2007). Sealing of chromosomal DNA nicks during nucleotide excision repair requires XRCC1 and DNA ligase III alpha in a cell-cycle-specific manner. Mol. Cell, 27, 311-23.

Edit history

Date	Action	Author
2004-01-29	Authored	Hoeijmakers JH
2015-06-05	Created	Orlic-Milacic M
2015-06-16	Revised	Orlic-Milacic M
2015-06-16	Edited	Orlic-Milacic M
2015-06-16	Authored	Orlic-Milacic M
2015-08-03	Reviewed	Fousteri M

Date	Action	Author
2023-11-28	Modified	Wright A

3 submitted entities found in this pathway, mapping to 3 Reactome entities

Input	UniProt Id	Input	UniProt Id	Input	UniProt Id
CCL1	P51946	RPA2	P15927	UBC	P0CG48

11. TGFBR2 MSI Frameshift Mutants in Cancer (R-HSA-3642279)

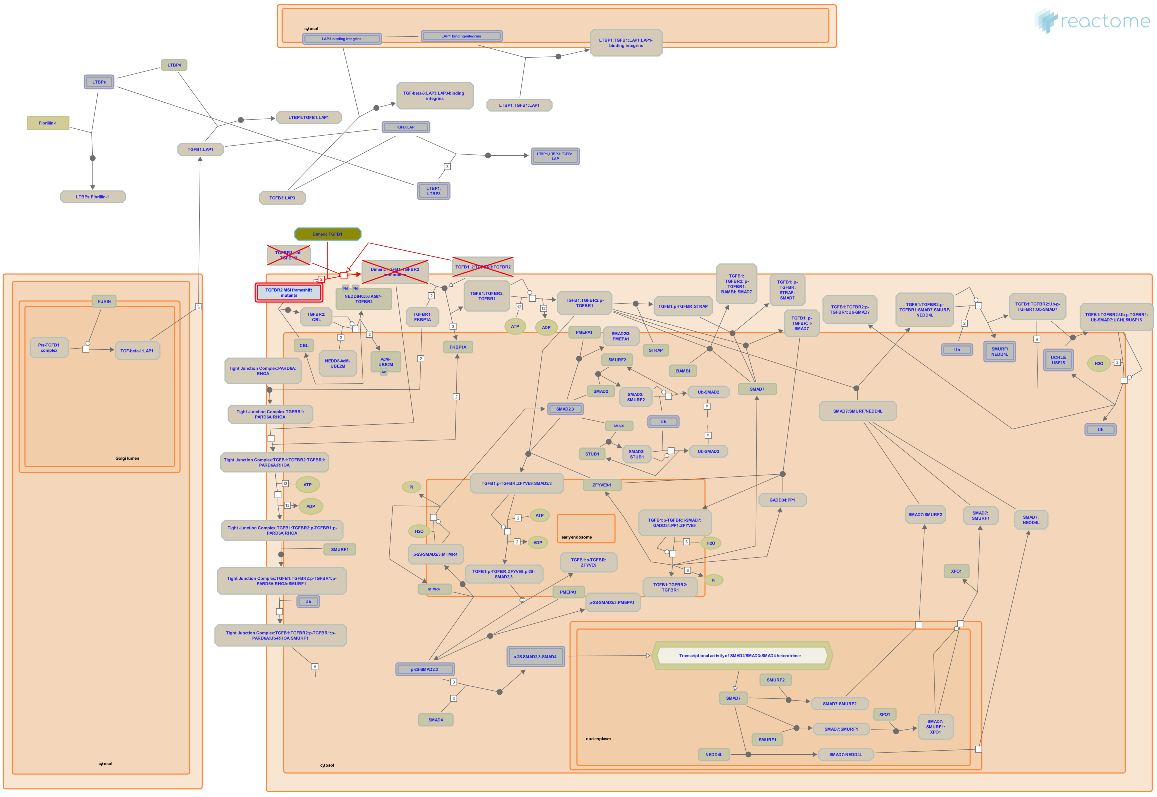

Diseases: cancer.

The short adenine repeat in the coding sequence of TGF-beta receptor II (TGFBR2) gene is frequently targeted by loss-of-function frameshift mutations in colon cancers with microsatellite instability (MSI). The 1- or 2-bp deletions in the adenine stretch of TGFBR2 cDNA introduce a premature stop codon that leads to degradation of the majority of mutant transcripts through nonsense-mediated decay or to production of a truncated TGFBR2 that cannot be presented on the cell surface. Cells that harbor TGFBR2 MSI frameshift mutations are resistant to TGF-beta (TGFB1)-mediated growth inhibition.

References

Fan RS, Sun L, Vogelstein B, Myeroff L, Wang J, Kinzler KW, ... Lutterbaugh J (1995). Inactivation of the type II TGF-beta receptor in colon cancer cells with microsatellite instability. Science, 268, 1336-8.

Sun L, Willson JK, Myeroff L, Gentry LE, Wang J, Yang J, ... Zborowska E (1995). Demonstration that mutation of the type II transforming growth factor beta receptor inactivates its tumor suppressor activity in replication error-positive colon carcinoma cells. J. Biol. Chem., 270, 22044-9.

Edit history

Date	Action	Author
2013-05-30	Created	Orlic-Milacic M
2013-08-08	Edited	Orlic-Milacic M
2013-08-08	Reviewed	Meyer S, Akhurst RJ
2013-08-08	Authored	Meyer S, Akhurst RJ, Orlic-Milacic M

Date	Action	Author
2023-11-28	Modified	Wright A

1 submitted entities found in this pathway, mapping to 1 Reactome entities

Input	UniProt Id
TGFB1	P01137

12. Sema4D induced cell migration and growth-cone collapse (R-HSA-416572)

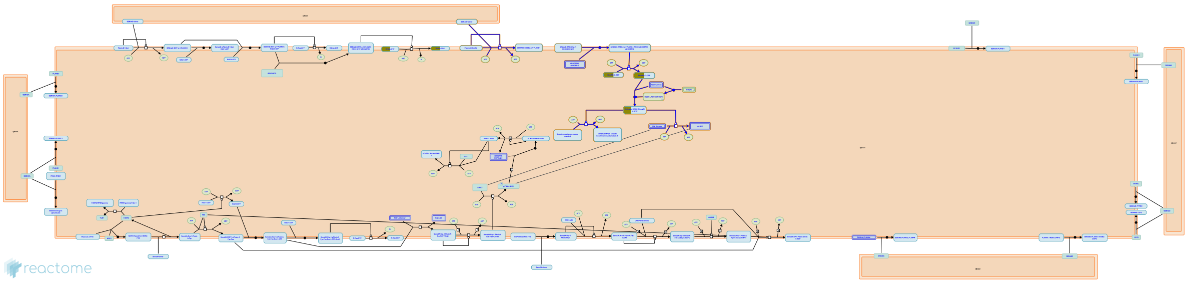

Cellular compartments: plasma membrane.

Sema4D-mediated attraction of endothelial cells requires Rho, but not R-Ras, signaling. Sema4D-mediated plexinB1 activation activates Rho and its downstream effector ROCK. ROCK then phosphorylates MLC to induce actomyosin stress fiber contraction and to direct the assembly of focal adhesion complexes and integrin-mediated adhesion.

References

Katoh H, Oinuma I & Negishi M (2005). Plexins: axon guidance and signal transduction. Cell Mol Life Sci, 62, 1363-71.

Offermanns S, Kuner R & Swiercz JM (2004). Plexin-B1/RhoGEF-mediated RhoA activation involves the receptor tyrosine kinase ErbB-2. J Cell Biol, 165, 869-80.

Zhou Y, Pasterkamp RJ & Gunput RA (2008). Semaphorin signaling: progress made and promises ahead. Trends Biochem Sci, 33, 161-70.

Furuyama T, Kogo M, Matsuya T, Inagaki S, Yamamoto T, Hirotani M, ... Nirasawa H (2002). Interaction of plexin-B1 with PDZ domain-containing Rho guanine nucleotide exchange factors. Biochem Biophys Res Commun, 297, 32-7.

Edit history

Date	Action	Author
2009-03-23	Edited	Garapati P V
2009-03-23	Authored	Garapati P V
2009-03-30	Created	Garapati P V
2009-09-02	Reviewed	Kumanogoh A, Kikutani H
2023-11-28	Modified	Wright A

1 submitted entities found in this pathway, mapping to 2 Reactome entities

Input	UniProt Id
RHOC	P08134, P61586

13. Gap-filling DNA repair synthesis and ligation in GG-NER (R-HSA-5696397)

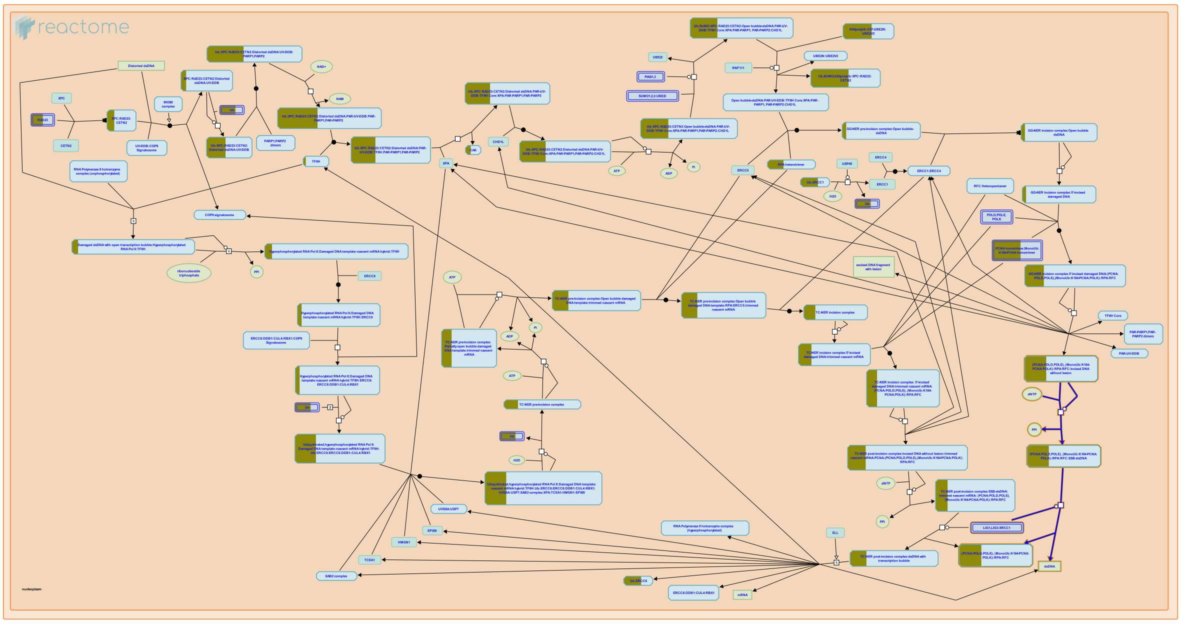

Cellular compartments: nucleoplasm.

Global genome nucleotide excision repair (GG-NER) is completed by DNA repair synthesis that fills the single stranded gap created after dual incision of the damaged DNA strand and excision of the ~27-30 bases long oligonucleotide that contains the lesion. DNA synthesis is performed by DNA polymerases epsilon or delta, or the Y family DNA polymerase kappa (POLK), which are loaded to the repair site after 5' incision (Staresincic et al. 2009, Ogi et al. 2010). DNA ligases LIG1 or LIG3 (as part of the LIG3:XRCC1 complex) ligate the newly synthesized stretch of oligonucleotides to the incised DNA strand (Moser et al. 2007).

References

Limsirichaikul S, Takenaka K, Yamashita S, Cloney R, Lehmann AR, Miki Y, ... Nakazawa Y (2010). Three DNA polymerases, recruited by different mechanisms, carry out NER repair synthesis in human cells. Mol. Cell, 37, 714-27.

Wijgers N, Staresincic L, Schärer OD, Gourdin AM, Fagbemi AF, Enzlin JH, ... Vermeulen W (2009). Coordination of dual incision and repair synthesis in human nucleotide excision repair. EMBO J., 28, 1111-20.

Fousteri M, Mullenders LH, Giakzidis I, Moser J, Caldecott K & Kool H (2007). Sealing of chromosomal DNA nicks during nucleotide excision repair requires XRCC1 and DNA ligase III alpha in a cell-cycle-specific manner. Mol. Cell, 27, 311-23.

Edit history

Date	Action	Author
2004-01-29	Authored	Hoeijmakers JH
2004-02-02	Authored	Gopinathrao G
2015-05-28	Created	Orlic-Milacic M
2015-06-16	Revised	Orlic-Milacic M
2015-06-16	Edited	Orlic-Milacic M

Date	Action	Author
2015-06-16	Authored	Orlic-Milacic M
2015-08-03	Reviewed	Fousteri M
2023-11-28	Modified	Wright A

2 submitted entities found in this pathway, mapping to 2 Reactome entities

Input	UniProt Id	Input	UniProt Id
RPA2	P15927	UBC	P0CG48

14. Sema4D in semaphorin signaling (R-HSA-400685)

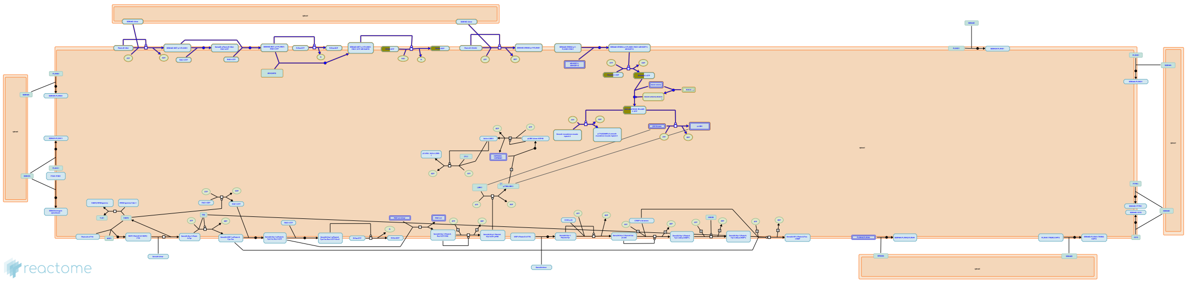

Semaphorin 4D (Sema 4D/CD100) is an axon guidance molecule with two disulfide-linked 150-kDa subunits. SEMA4D is structurally defined by a conserved 500-amino acid extracellular domain with 16 cysteines (sema domain) and also an Ig-like domain C-terminal to the sema domain. Sema4D is expressed on the cell surface as a homodimer; cysteine 679 within the sema domain is required for this dimerization.

The main receptors for Sema4D are plexin-B1 and CD72. The activation of plexins by semaphorins initiates a variety of signaling processes that involve several small GTPases of the Ras and Rho families. Sema4D-Plexin-B1 interaction appears to mediate different and sometimes opposite effects depending on the cellular context. Plexin-B1 activation inhibits integrin-mediated cell attachment and cell migration through the activation of the R-RasGAP activity inherent to plexin-B1 or through the inhibition of RhoA. However, activation of plexin-B1 by Sema4D stimulates the migration of endothelial cells by mediating the activation of RhoA.

References

Kumanogoh A & Kikutani H (2004). Biological functions and signaling of a transmembrane semaphorin, CD100/Sema4D. Cell Mol Life Sci, 61, 292-300.

Katoh H, Oinuma I & Negishi M (2005). Plexins: axon guidance and signal transduction. Cell Mol Life Sci, 62, 1363-71.

Guan KL, Aurandt J & Kruger RP (2005). Semaphorins command cells to move. Nat Rev Mol Cell Biol, 6, 789-800.

Edit history

Date	Action	Author
2009-03-23	Edited	Garapati P V
2009-03-23	Authored	Garapati P V
2009-03-24	Created	Garapati P V
2009-09-02	Reviewed	Kumanogoh A, Kikutani H
2023-11-16	Modified	Wright A

1 submitted entities found in this pathway, mapping to 2 Reactome entities

Input	UniProt Id
RHOC	P08134, P61586

15. Membrane binding and targetting of GAG proteins (R-HSA-174490)

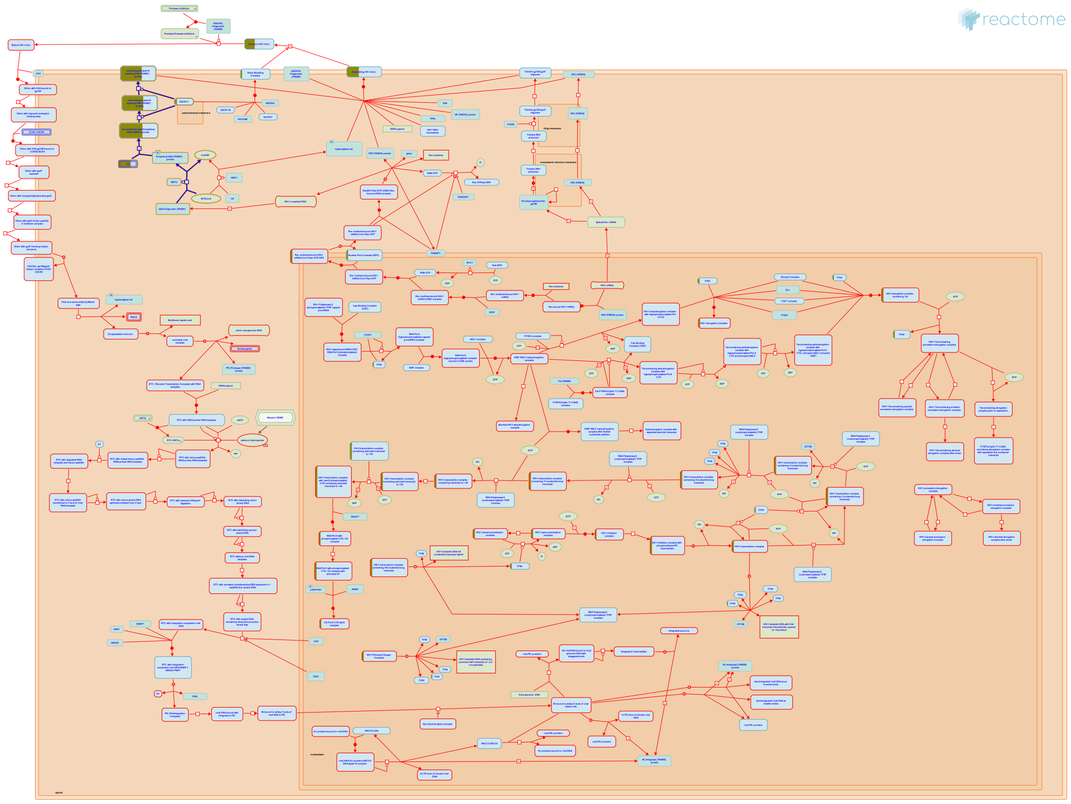

Diseases: Human immunodeficiency virus infectious disease.

One of the mysteries of Gag protein involvement in HIV virion assembly is how the proteins are targeted to the proper membrane for budding. Infectious retroviruses do not bud from all of the available membrane surfaces within an infected cell, but primarily from the plasma membrane, which constitutes a small proportion of the total membrane surface in most cells. In polarized cells, the sites of budding are further restricted to the basolateral membrane.

References

Resh MD & Perlman M (2006). Identification of an intracellular trafficking and assembly pathway for HIV-1 gag. Traffic, 7, 731-45.

Sundquist WI & Morita E (2004). Retrovirus budding. Annu Rev Cell Dev Biol, 20, 395-425.

Edit history

Date	Action	Author
2006-02-18	Created	Gopinathrao G
2013-03-07	Authored	Gillespie ME
2013-05-21	Reviewed	Dube M
2023-11-28	Modified	Wright A

2 submitted entities found in this pathway, mapping to 2 Reactome entities

Input	UniProt Id	Input	UniProt Id
TSG101	Q99816	UBC	P0CG48

16. Synthesis And Processing Of GAG, GAGPOL Polyproteins (R-HSA-174495)

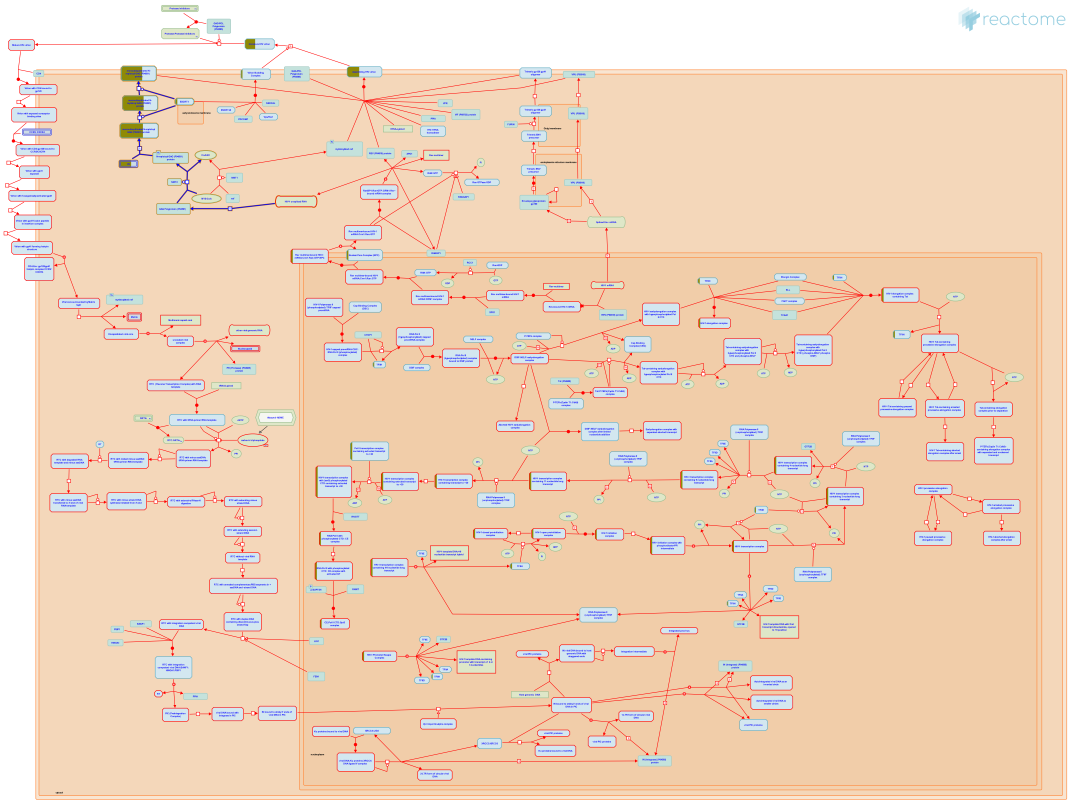

Diseases: Human immunodeficiency virus infectious disease.

Evidence suggests that the RNA molecules used for the synthesis of Gag and Gag-Pro-Pol are not the same molecules that are packaged into virions. Gag proteins do not appear to aggregate around and capture the RNA contained in the polyribosome from which they emerged, but rather bind to and ultimately encapsidate free transcripts elsewhere. During the replication of retroviruses, large numbers of Gag molecules must be generated to serve as precursors to the structural proteins of the virions. Retroviruses have developed a mechanism that permits expression of the Gag protein at high levels relative to the protein sequences encoded in the pro and pol genes, while retaining coregulated expression. This linkage results from the use of the same initiation codon in the same mRNA to express the gag, pro, and pol genes. Translation of this RNA leads occasionally to synthesis of a fusion protein that is usually called the Gag-Pol precursor but is now more appropriately called the Gag-Pro-Pol precursor

References

Varmus HE, Hughes SH & Coffin JM (1997). *Synthesis, Assembly, and Processing of Viral Proteins, Retroviruses* .

Edit history

Date	Action	Author
2006-02-18	Created	Gopinathrao G
2013-03-07	Authored	Gillespie ME
2013-05-21	Reviewed	Dube M
2023-11-28	Modified	Wright A

2 submitted entities found in this pathway, mapping to 2 Reactome entities

Input	UniProt Id	Input	UniProt Id
TSG101	Q99816	UBC	P0CG48

17. RHOC GTPase cycle (R-HSA-9013106)

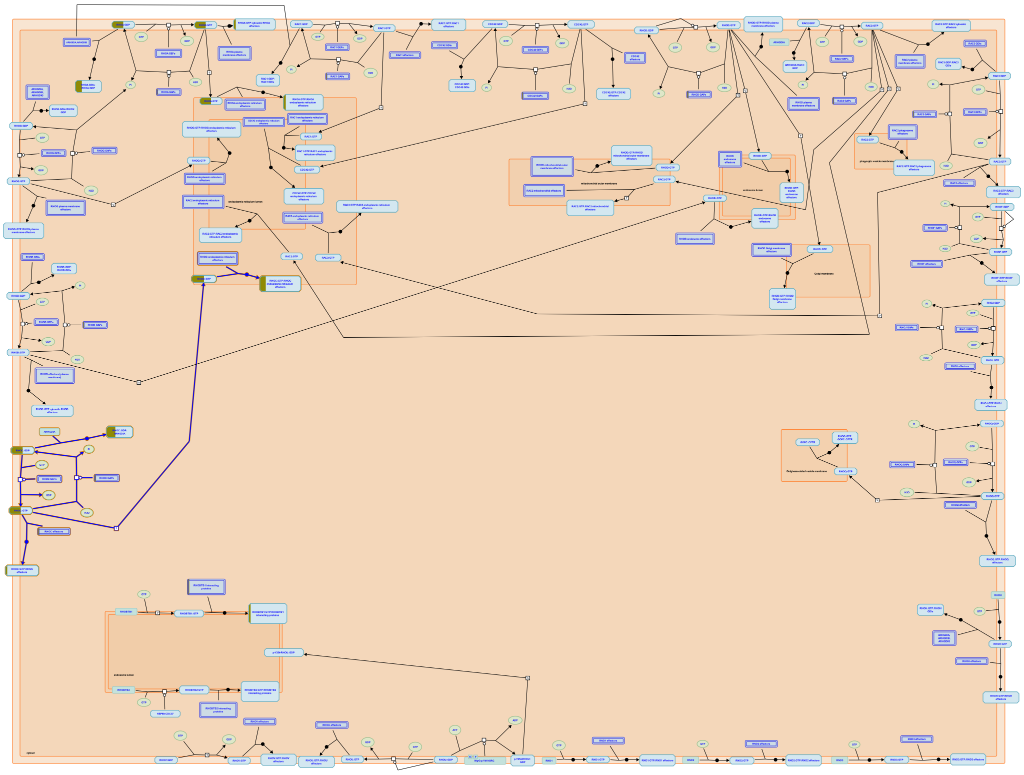

This pathway catalogues RHOC guanine nucleotide exchange factors (GEFs), GTPase activator proteins (GAPs), GDP dissociation inhibitors (GDIs) and RHOC effectors. RHOC belongs to the RHOA subfamily of RHO GTPases and shares 85% sequence identity with RHOA and RHOB (Wheeler and Ridley 2004). Like RHOA and RHOB, RHOC regulates the cytoskeleton and is involved in cell adhesion and migration (Guan et al. 2018). RHOC contributes to invasiveness and metastatic potential of cancer cells (Bravo-Cordero et al. 2014; Guan et al. 2018; Thomas et al. 2019).

References

Pranatharthi A, Thomas P, Ross C & Srivastava S (2019). RhoC: a fascinating journey from a cytoskeletal organizer to a Cancer stem cell therapeutic target. J. Exp. Clin. Cancer Res., 38, 328.

Chen S, Zhao Y & Guan X (2018). The role of RhoC in malignant tumor invasion, metastasis and targeted therapy. Histol. Histopathol., 33, 255-260.

Condeelis JS, Bravo-Cordero JJ & Hodgson L (2014). Spatial regulation of tumor cell protrusions by RhoC. Cell Adh Migr, 8, 263-7.

Ridley AJ & Wheeler AP (2004). Why three Rho proteins? RhoA, RhoB, RhoC, and cell motility. Exp. Cell Res., 301, 43-9.

Edit history

Date	Action	Author
2017-07-23	Created	Orlic-Milacic M
2020-07-14	Authored	Orlic-Milacic M
2021-02-05	Reviewed	Fort P

Date	Action	Author
2021-02-25	Edited	Orlic-Milacic M
2023-11-17	Modified	Wright A

2 submitted entities found in this pathway, mapping to 3 Reactome entities

Input	UniProt Id	Input	UniProt Id
RACGAP1	Q9H0H5	RHOC	P08134, P61586

18. Dual incision in TC-NER (R-HSA-6782135)

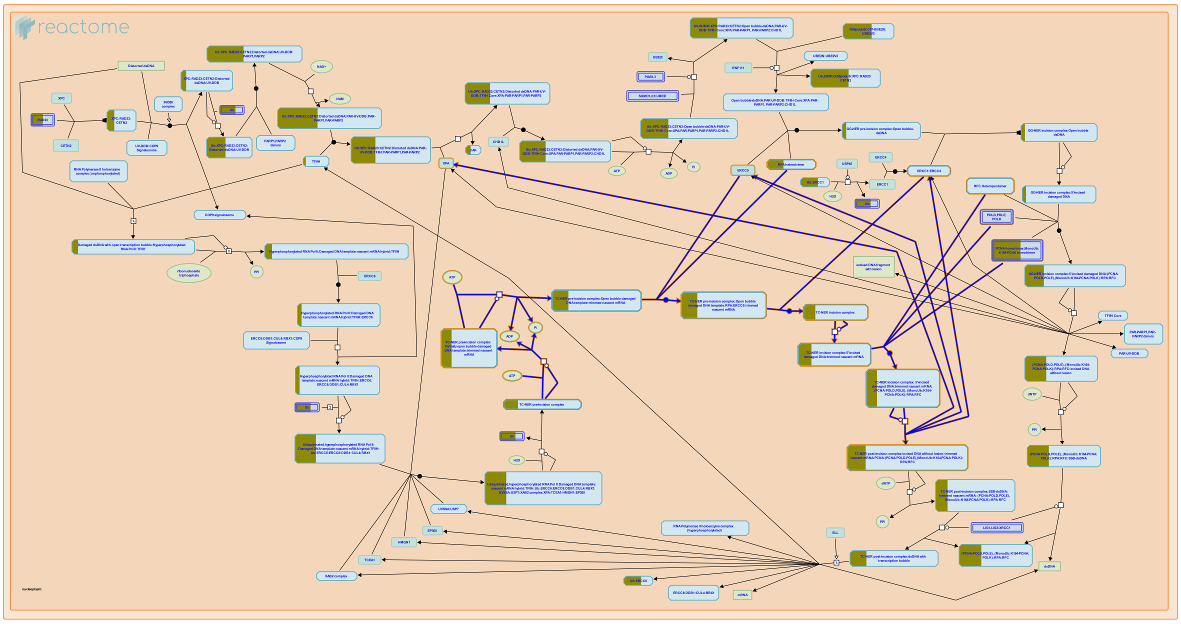

Cellular compartments: nucleoplasm.

In transcription-coupled nucleotide excision repair (TC-NER), similar to global genome nucleotide excision repair (GG-NER), the oligonucleotide that contains the lesion is excised from the open bubble structure via dual incision of the affected DNA strand. 5' incision by the ERCC1:ERCC4 (ERCC1:XPF) endonuclease precedes 3' incision by ERCC5 (XPG) endonuclease. In order for the TC-NER pre-incision complex to assemble and the endonucleases to incise the damaged DNA strand, the RNA polymerase II (RNA Pol II) complex has to backtrack - reverse translocate from the damage site. Although the mechanistic details of this process are largely unknown in mammals, it may involve ERCC6/ERCC8-mediated chromatin remodelling/ubiquitination events, the DNA helicase activity of the TFIIH complex and TCEA1 (TFIIS)-stimulated cleavage of the 3' protruding end of nascent mRNA by RNA Pol II (Donahue et al. 1994, Lee et al. 2002, Sarker et al. 2005, Vermeulen and Fousteri 2013, Hanawalt and Spivak 2008, Staresincic et al. 2009, Epshtein et al. 2014).

References

Fousteri M & Vermeulen W (2013). Mammalian transcription-coupled excision repair. Cold Spring Harb Perspect Biol, 5, a012625.

Lippard SJ, Sharp PA, Lee KB & Wang D (2002). Transcription-coupled and DNA damage-dependent ubiquitination of RNA polymerase II in vitro. Proc. Natl. Acad. Sci. U.S.A., 99, 4239-44.

Ueberheide B, Kamarthapu V, Nudler E, Proshkin S, McGary K, Mironov A, ... Svetlov V (2014). UvrD facilitates DNA repair by pulling RNA polymerase backwards. Nature, 505, 372-7.

Donahue BA, Reines D, Taylor JS, Yin S & Hanawalt PC (1994). Transcript cleavage by RNA polymerase II arrested by a cyclobutane pyrimidine dimer in the DNA template. Proc. Natl. Acad. Sci. U.S.A., 91, 8502-6.

Kostek S, Tainer JA, Sarker AH, Ng C, Nogales E, Cooper PK, ... Shin DS (2005). Recognition of RNA polymerase II and transcription bubbles by XPG, CSB, and TFIIH: insights for transcription-coupled repair and Cockayne Syndrome. Mol. Cell, 20, 187-98.

Edit history

Date	Action	Author
2004-01-29	Authored	Hoeijmakers JH
2004-02-11	Authored	Gopinathrao G
2015-05-28	Edited	Orlic-Milacic M
2015-06-04	Created	Orlic-Milacic M
2015-06-16	Revised	Orlic-Milacic M
2015-06-16	Authored	Orlic-Milacic M
2015-08-03	Reviewed	Fousteri M
2023-11-28	Modified	Wright A

3 submitted entities found in this pathway, mapping to 3 Reactome entities

Input	UniProt Id	Input	UniProt Id	Input	UniProt Id
CCL1	P51946	RPA2	P15927	UBC	P0CG48

Interactors found in this pathway (1)

Input	UniProt Id	Interacts with	Input	UniProt Id	Interacts with
RPA2	P15927	P23025			

19. Loss of Function of TGFBR2 in Cancer (R-HSA-3642278)

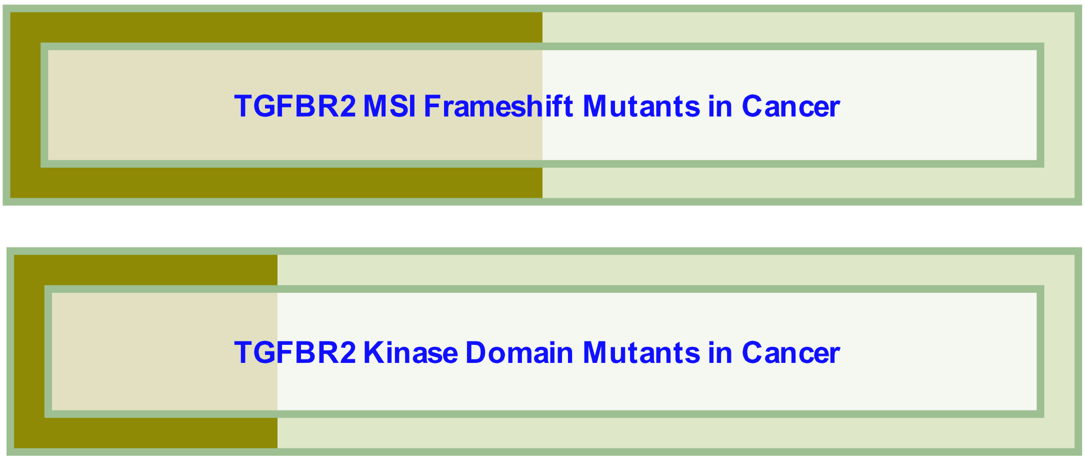

Diseases: cancer.

Loss-of-function of transforming growth factor-beta receptor II (TGFBR2) is most prevalent in colorectal cancer. Over 60% of colorectal cancers with microsatellite instability (MSI) harbor inactivating mutations in both alleles of TGFBR2, mostly 1 or 2 bp deletions in the 10 bp adenine repeat that codes for three lysine residues in the extracellular domain of TGFBR2. These small deletions result in a frameshift and a premature stop codon (Markowitz et al. 1995). TGFBR2 kinase domain (KD) mutations are found in ~20% of microsatellite stable (MSS) colorectal cancers and these are mostly missense mutations that results in substitution of conserved amino acids in the kinase domain (Grady et al. 1999), likely impairing the catalytic activity of TGFBR2 KD mutants. The silencing of TGFBR2 gene via promoter methylation has been reported in B-cell lymphoma (Chen et al. 2007). Knockout of murine Tgfbr2 in colonic epithelium promotes azoxymethane-induced colon cancer formation (Biswas et al. 2004) and increases the number of adenomas and adenocarcinomas in Apc+/- mice (Munoz et al. 2006).

References

Muñoz NM, Madison BB, Washington MK, Moses HL, Pozzi A, Chytil A, ... Grady WM (2006). Transforming growth factor beta receptor type II inactivation induces the malignant transformation of intestinal neoplasms initiated by Apc mutation. Cancer Res., 66, 9837-44.

Fan RS, Sun L, Vogelstein B, Myeroff L, Wang J, Kinzler KW, ... Lutterbaugh J (1995). Inactivation of the type II TGF-beta receptor in colon cancer cells with microsatellite instability. Science, 268, 1336-8.

Romero-Gallo J, Wirth PS, Moses HL, Washington K, Chytil A, Biswas S, ... Grady WM (2004). Transforming growth factor beta receptor type II inactivation promotes the establishment and progression of colon cancer. Cancer Res., 64, 4687-92.

Ghosh P, Rezanka L, O'Farrell TJ, Chen G, Sasaki CY, Osawa H, ... Longo DL (2007). Resistance to TGF-beta 1 correlates with aberrant expression of TGF-beta receptor II in human B-cell lymphoma cell lines. Blood, 109, 5301-7.

Kinzler KW, Willson JK, Grady WM, Chang J, Vogelstein B, Swinler SE, ... Thiagalingam S (1999). Mutational inactivation of transforming growth factor beta receptor type II in microsatellite stable colon cancers. Cancer Res., 59, 320-4.

Edit history

Date	Action	Author
2013-05-30	Created	Orlic-Milacic M
2013-08-08	Edited	Orlic-Milacic M
2013-08-08	Reviewed	Meyer S, Akhurst RJ
2013-08-08	Authored	Meyer S, Akhurst RJ, Orlic-Milacic M
2023-10-12	Modified	Weiser JD

1 submitted entities found in this pathway, mapping to 1 Reactome entities

Input	UniProt Id
TGFB1	P01137

20. TGFBR1 LBD Mutants in Cancer (R-HSA-3656535)

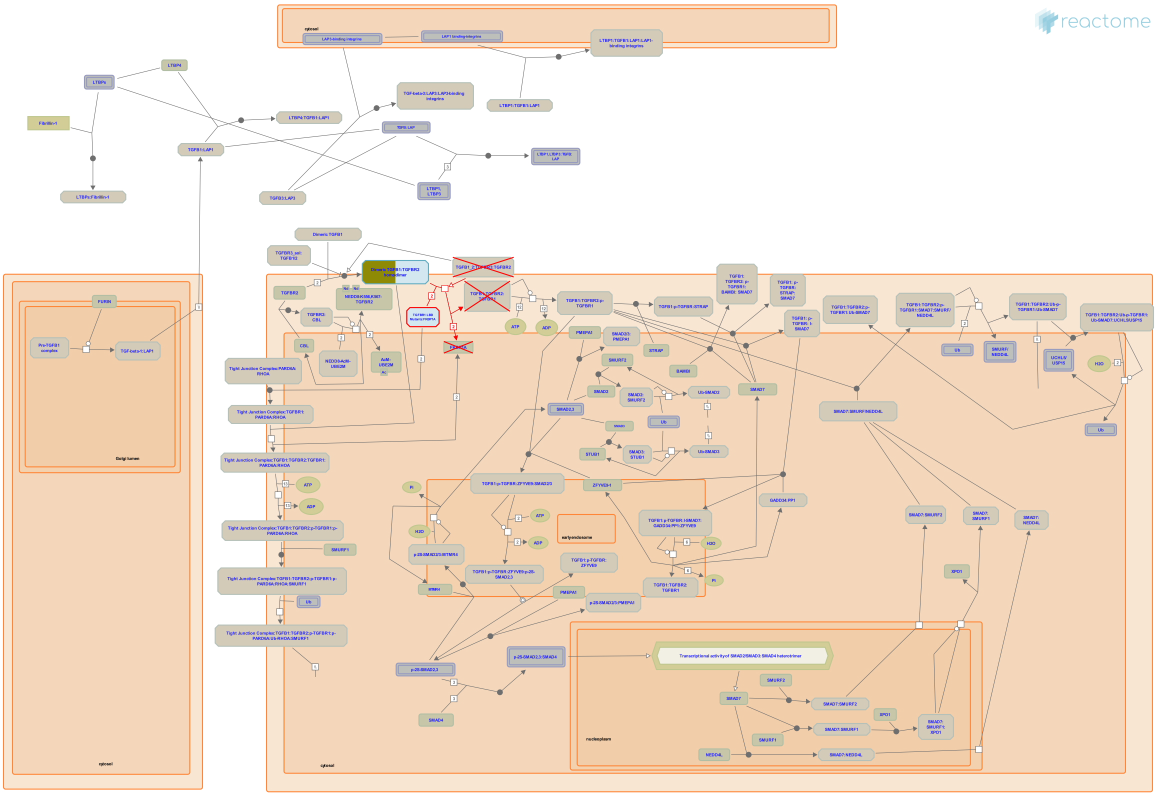

Diseases: cancer.

Mutations in the ligand-binding domain (LBD) of TGF-beta receptor 1 (TGFBR1) have been reported as germline mutations in Ferguson-Smith tumor (multiple self-healing squamous epithelioma - MSSE), an autosomal-dominant skin cancer condition (Ferguson-Smith et al. 1934, Ferguson-Smith et al. 1971), with tumors frequently showing loss of heterozygosity of the wild-type TGFBR1 allele (Goudie et al. 2011). Somatic mutations in the LBD of TGFBR1 have been reported in esophageal carcinoma (Dulak et al. 2013).

References

Gerdes AM, Reversade B, Lee H, Ferguson-Smith MA, Whittaker S, Christie L, ... Verma C (2011). Multiple self-healing squamous epithelioma is caused by a disease-specific spectrum of mutations in TGFBR1. Nat. Genet., 43, 365-9.

Wallace DC, James ZH, Renwick JH & Ferguson-Smith MA (1971). Multiple self-healing squamous epithelioma. Birth Defects Orig. Artic. Ser., 7, 157-63.

Luketich JD, Ogino S, Pennathur A, Imamura Y, Stewart C, Voet D, ... Fox C (2013). Exome and whole-genome sequencing of esophageal adenocarcinoma identifies recurrent driver events and mutational complexity. Nat. Genet., 45, 478-86.

Ferguson-Smith J (1934). A case of multiple primary squamous-celled carcinomata in a young man, with spontaneous healing. Br. J. Dermatol., 46, 267-72.

Edit history

Date	Action	Author
2013-06-05	Created	Orlic-Milacic M

Date	Action	Author
2013-08-08	Edited	Orlic-Milacic M
2013-08-08	Reviewed	Meyer S, Akhurst RJ
2013-08-08	Authored	Meyer S, Akhurst RJ, Orlic-Milacic M
2023-11-28	Modified	Wright A

1 submitted entities found in this pathway, mapping to 1 Reactome entities

Input	UniProt Id
TGFB1	P01137

21. TGFBR2 Kinase Domain Mutants in Cancer (R-HSA-3645790)

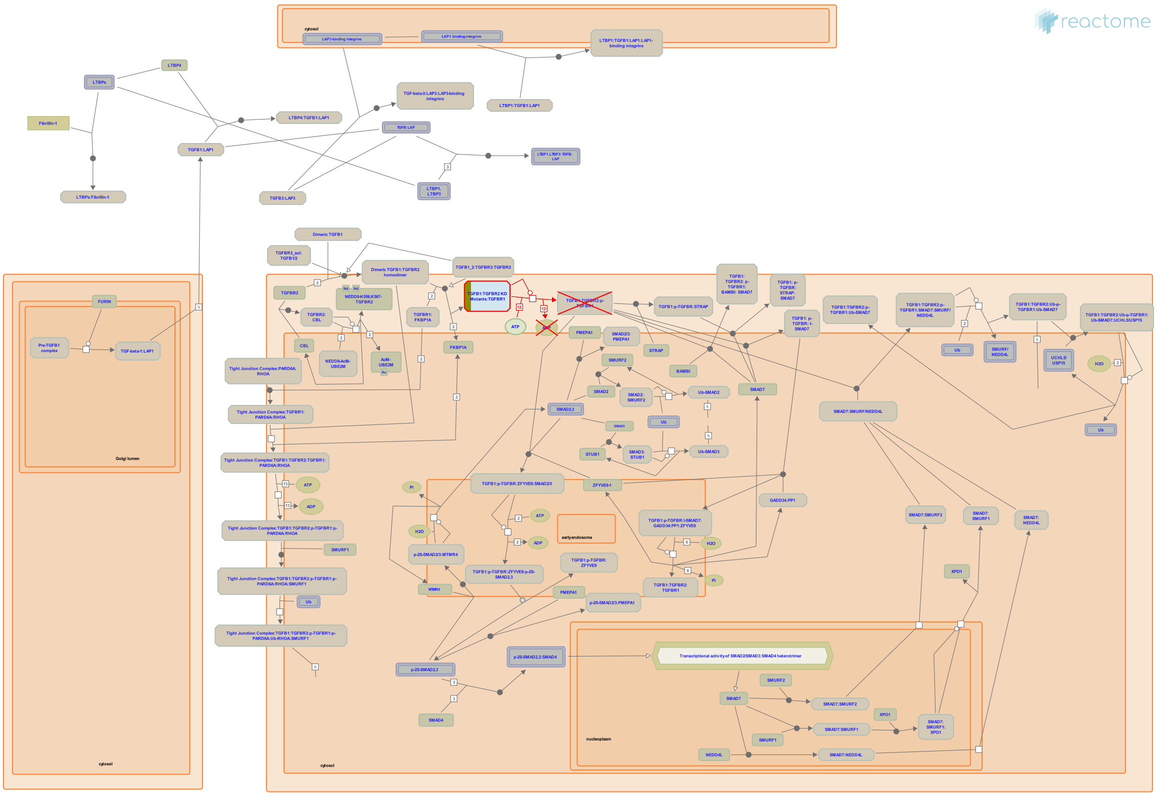

Diseases: cancer.

Missense mutations in the kinase domain (KD) of TGF-beta receptor II (TGFBR2) are found in ~20% of microsatellite stable (MSS) colon cancers and make affected tumors resistant to TGF-beta (TGFB1)-mediated growth inhibition (Grady et al. 1999). While both alleles of TGFBR2 are affected by inactivating mutations in MSS colorectal cancer (Grady et al. 1999), a study of MSS esophageal carcinoma indicates that TGFBR2 KD mutations may function in a dominant-negative way (Tanaka et al. 2000). KD mutations in TGFBR2 are rarely reported in microsatellite instable (MSI) colorectal cancer (Parsons et al. 1995, Takenoshita et al. 1997).

References

Tanaka S, Ohno S, Sugimachi K, Mori M & Mafune K (2000). A dominant negative mutation of transforming growth factor-beta receptor type II gene in microsatellite stable oesophageal carcinoma . Br. J. Cancer, 82, 1557-60.

Willson JK, Vogelstein B, Myeroff L, Kinzler KW, Markowitz SD, Parsons R & Liu B (1995). Microsatellite instability and mutations of the transforming growth factor beta type II receptor gene in colorectal cancer. Cancer Res., 55, 5548-50.

Kinzler KW, Willson JK, Grady WM, Chang J, Vogelstein B, Swinler SE, ... Thiagalingam S (1999). Mutational inactivation of transforming growth factor beta receptor type II in microsatellite stable colon cancers. Cancer Res., 59, 320-4.

Harris CC, Takenoshita S, Bennett WP, Hagiwara K, Yokota J, Tani M & Nagashima M (1997). Mutation analysis of coding sequences of the entire transforming growth factor beta type II receptor gene in sporadic human colon cancer using genomic DNA and intron primers. Oncogene, 14, 1255-8.

Edit history

Date	Action	Author
2013-05-30	Created	Orlic-Milacic M
2013-08-08	Edited	Orlic-Milacic M
2013-08-08	Reviewed	Meyer S, Akhurst RJ
2013-08-08	Authored	Meyer S, Akhurst RJ, Orlic-Milacic M
2023-11-28	Modified	Wright A

1 submitted entities found in this pathway, mapping to 1 Reactome entities

Input	UniProt Id
TGFB1	P01137

22. Molecules associated with elastic fibres (R-HSA-2129379)

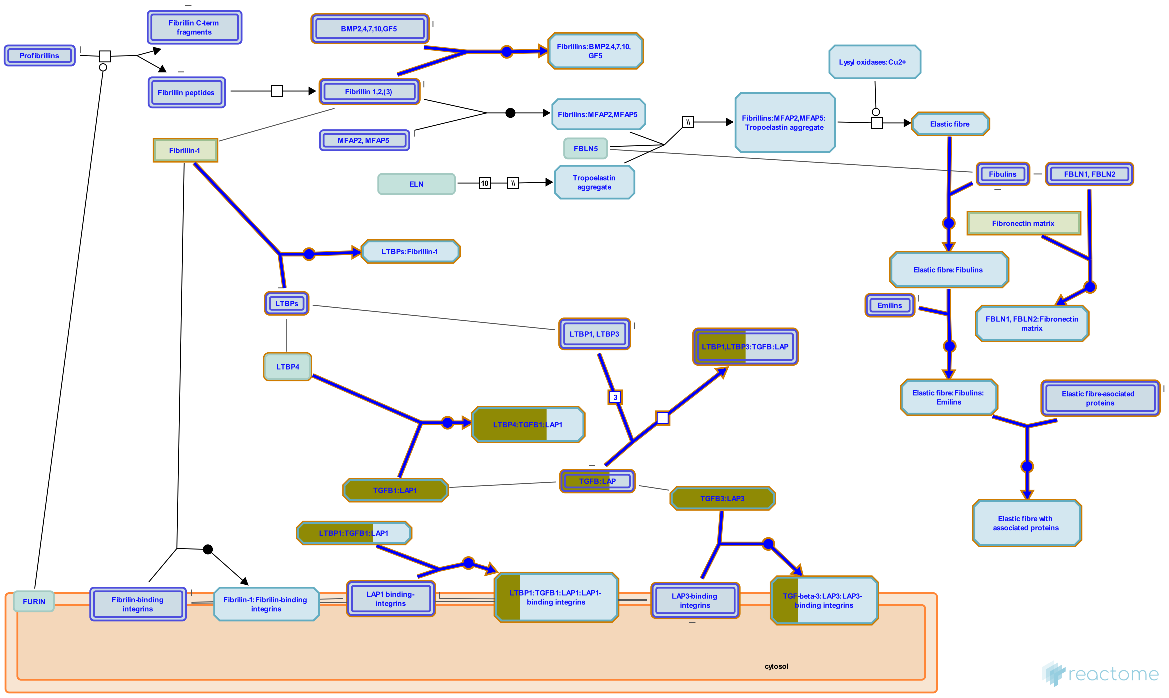

Proteins found associated with microfibrils include vitronectin (Dahlback et al. 1990), latent transforming growth factor beta-binding proteins (Kielty et al. 2002, Munger & Sheppard 2011), emilin (Bressan et al. 1993, Mongiat et al. 2000), members of the microfibrillar-associated proteins (MFAPs, Gibson et al.1996), and fibulins (Roark et al. 1995, Yanagisawa et al. 2002). The significance of these interactions is not well understood but may help mediate elastin-fibrillin interactions during elastic fibre assembly.

Proteoglycans such as versican (Isogai et al. 2002), biglycan, and decorin (Reinboth et al. 2002) can interact with the microfibrils. They confer specific properties including hydration, impact absorption, molecular sieving, regulation of cellular activities, mediation of growth factor association, and release and transport within the extracellular matrix (Buczek-Thomas et al. 2002). In addition, glycosaminoglycans have been shown to interact with tropoelastin through its lysine side chains (Wu et al. 1999) regulating tropoelastin assembly (Tu and Weiss, 2008).

References

Kielty CM, Shuttleworth CA & Sherratt MJ (2002). Elastic fibres. J Cell Sci, 115, 2817-28.

Wagenseil JE & Mecham RP (2007). New insights into elastic fiber assembly. Birth Defects Res. C Embryo Today, 81, 229-40.

Edit history

Date	Action	Author
2012-02-21	Created	Jupe S
2012-04-30	Authored	Jupe S
2012-11-02	Reviewed	Muiznieks LD
2012-11-12	Edited	Jupe S
2023-11-16	Modified	Wright A

1 submitted entities found in this pathway, mapping to 2 Reactome entities

Input	UniProt Id
TGFB1	P01137, P10600

23. Recognition of DNA damage by PCNA-containing replication complex (R-HSA-110314)

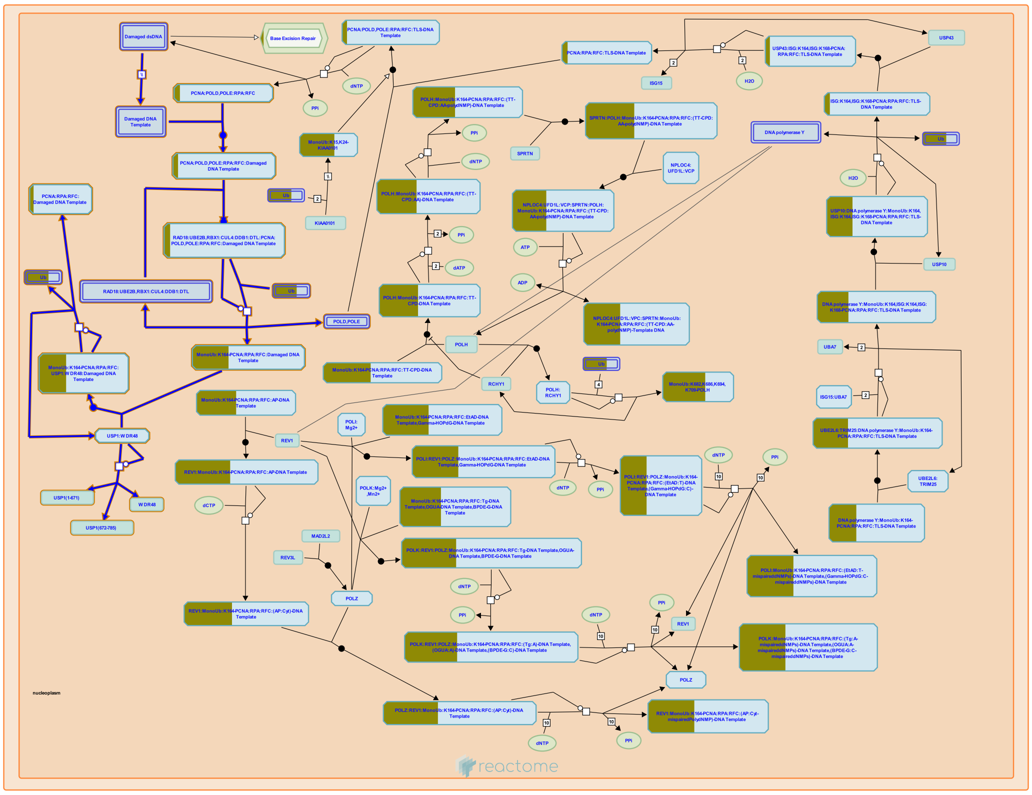

Cellular compartments: nucleoplasm.

Damaged double strand DNA (dsDNA) cannot be successfully used as a template by replicative DNA polymerase delta (POLD) and epsilon (POLE) complexes (Hoege et al. 2002). When the replication complex composed of PCNA, RPA, RFC and POLD or POLE stalls at a DNA damage site, PCNA becomes monoubiquitinated by RAD18 bound to UBE2B (RAD6). POLD or POLE dissociate from monoubiquitinated PCNA, while Y family DNA polymerases - REV1, POLH (DNA polymerase eta), POLK (DNA polymerase kappa) and POLI (DNA polymerase iota) - bind monoubiquitinated PCNA through their ubiquitin binding and PCNA binding motifs, resulting in a polymerase switch and initiation of translesion synthesis (TLS) (Hoege et al. 2002, Friedberg et al. 2005).

References

Friedberg EC, Lehmann AR & Fuchs RP (2005). Trading places: how do DNA polymerases switch during translesion DNA synthesis?. Mol. Cell, 18, 499-505.

Moldovan GL, Hoege C, Pfander B, Pyrowolakis G & Jentsch S (2002). RAD6-dependent DNA repair is linked to modification of PCNA by ubiquitin and SUMO. Nature, 419, 135-41.

Edit history

Date	Action	Author
2004-01-29	Created	Gopinathrao G
2014-12-11	Edited	Orlic-Milacic M
2014-12-11	Authored	Orlic-Milacic M

Date	Action	Author
2015-01-07	Reviewed	Borowiec JA
2023-11-17	Modified	Wright A

2 submitted entities found in this pathway, mapping to 2 Reactome entities

Input	UniProt Id	Input	UniProt Id
RPA2	P15927	UBC	P0CG48

24. Defective Inhibition of DNA Recombination at Telomere Due to DAXX Mutations (R-HSA-9670613)

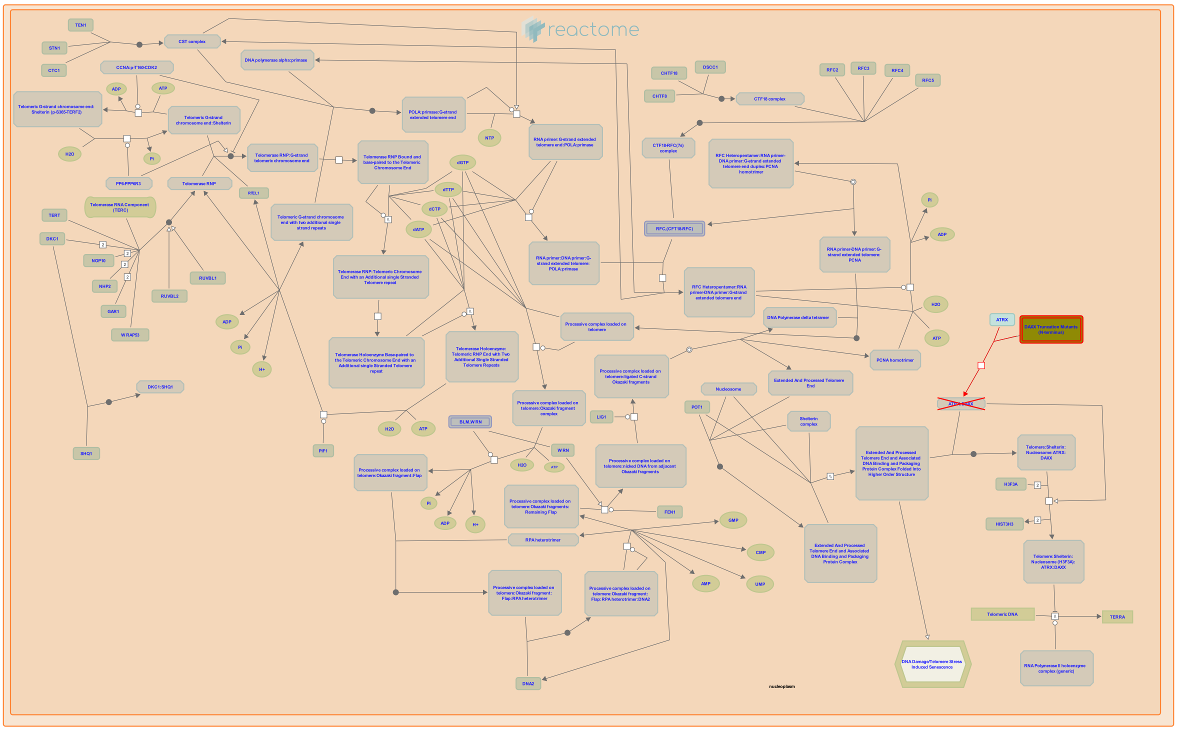

Cellular compartments: nucleoplasm.

Diseases: cancer.

A small portion of tumors that are positive for alternative lengthening of telomeres (ALT) markers and negative for mutations in the ATRX gene harbor loss-of-function mutations in the DAXX gene, which encodes the ATRX binding partner DAXX. For review, please refer to Gocha et al. 2013, and Pickett and Reddel 2015.

References

Groden J, Gocha AR & Harris J (2013). Alternative mechanisms of telomere lengthening: permissive mutations, DNA repair proteins and tumorigenic progression. Mutat. Res., 743, 142-50.

Reddel RR & Pickett HA (2015). Molecular mechanisms of activity and derepression of alternative lengthening of telomeres. Nat. Struct. Mol. Biol., 22, 875-80.

Edit history

Date	Action	Author
2019-12-11	Created	Orlic-Milacic M
2020-04-30	Authored	Orlic-Milacic M
2020-11-05	Reviewed	Meeker AK
2020-11-09	Edited	Orlic-Milacic M
2020-11-13	Reviewed	Reddel RR
2020-11-16	Edited	Orlic-Milacic M
2023-03-08	Modified	Matthews L

1 submitted entities found in this pathway, mapping to 1 Reactome entities

Input	UniProt Id
DAXX	Q9UER7

Interactors found in this pathway (1)

Input	UniProt Id	Interacts with	Input	UniProt Id	Interacts with
DAXX	Q9UER7	P46100			

25. Loss of Function of SMAD2/3 in Cancer (R-HSA-3304349)

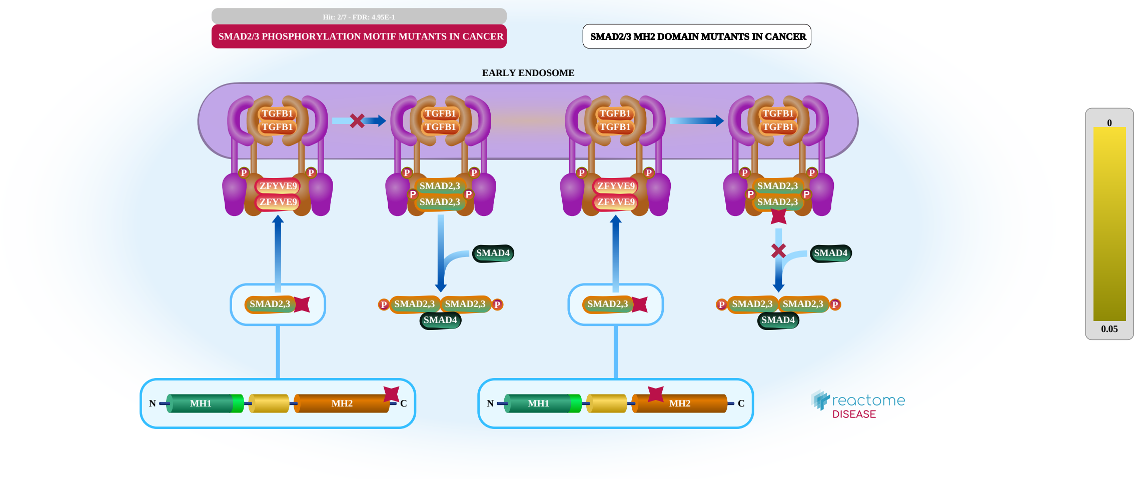

Diseases: cancer.

Loss-of-function of SMAD2 and SMAD3 in cancer occurs less frequently than the loss of SMAD4 function and was studied in most detail in colorectal cancer (Fleming et al. 2013).

Similarly to SMAD4, coding sequence mutations in SMAD2 and SMAD3 in cancer cluster in the MH2 domain, involved in the formation of transcriptionally active heterotrimers with SMAD4. Another region of SMAD2 and SMAD3 that is frequently mutated in cancer is the phosphorylation motif Ser-Ser-X-Ser at the very C-terminus (Fleming et al. 2013). The phosphorylation of this conserved motif by the activated TGF-beta receptor complex is an essential step in SMAD2 and SMAD3 activation and a prerequisite for the formation of heterotrimers with SMAD4 (Chacko et al. 2001, Chacko et al. 2004).

Smad2 knockout mice die at embryonic day 8.5, with impaired visceral endoderm function and deficiency in mesoderm formation. Smad2+/- heterozygotes appear normal and are fertile (Hamamoto et al. 2002). While polyps of compound Smad2+/-;Apc+/- mice show no difference in the number, size or histopathology from the polyps of Apc+/- mice (Takaku et al. 2002, Hamamoto et al. 2002), Smad2+/-;Apc+/- mice develop extremely large intestinal tumors and multiple invasive cancers not observed in Apc+/- mice. Therefore, loss of Smad2 does not contribute to initiation of intestinal tumorigenesis, but accelerates malignant progression (Hamamoto et al. 2002). Smad3 knockout mice are viable and fertile but die between 4 and 6 months of age from colorectal adenocarcinoma (Zhu et al. 1998), indicating that the loss of Smad3 initiates intestinal tumorigenesis.

References

Okada H, Miyazono K, Hamamoto T, Kitamura T, Kato M, Kawabata M & Beppu H (2002). Compound disruption of smad2 accelerates malignant progression of intestinal tumors in apc knockout mice. Cancer Res., 62, 5955-61.

Shi G, De Caestecker M, Lin K, Chacko BM, Hayward LJ, Tiwari A, ... Lam S (2004). Structural basis of heteromeric smad protein assembly in TGF-beta signaling. Mol Cell, 15, 813-23.

Parada LF, Zhu Y, Graff JM & Richardson JA (1998). Smad3 mutant mice develop metastatic colorectal cancer. Cell, 94, 703-14.

Correia JJ, Lam SS, de Caestecker MP, Qin B, Chacko BM & Lin K (2001). The L3 loop and C-terminal phosphorylation jointly define Smad protein trimerization. Nat. Struct. Biol., 8, 248-53.

Mouradov D, Jorissen RN, Jones IT, Tsui C, Palmieri M, Sieber OM, ... Zhao Q (2013). SMAD2, SMAD3 and SMAD4 mutations in colorectal cancer. Cancer Res., 73, 725-35.

Edit history

Date	Action	Author
2013-04-23	Created	Orlic-Milacic M
2013-05-03	Edited	Jassal B
2013-08-08	Edited	Orlic-Milacic M
2013-08-08	Reviewed	Meyer S, Akhurst RJ
2013-08-08	Authored	Meyer S, Akhurst RJ, Orlic-Milacic M
2023-10-12	Modified	Weiser JD

2 submitted entities found in this pathway, mapping to 2 Reactome entities

Input	UniProt Id	Input	UniProt Id
TGFB1	P01137	ZFYVE9	O95405-1

6. Identifiers found

Below is a list of the input identifiers that have been found or mapped to an equivalent element in Reactome, classified by resource.

71 of the submitted entities were found, mapping to 99 Reactome entities

Input	UniProt Id	Input	UniProt Id	Input	UniProt Id
ACTA2	P62736	ANKRD9	Q96BM1	ATG4D	Q86TL0
ATP1A1	P05023	BTK	Q06187	CCL1	P51946
CCL20	P78556	CEBPE	Q15744	COX7A2L	O14548
CPNE1	Q99829	CTSK	P43235	DAD1	P61803
DAXX	Q9UER7	DNM2	P50570	DSG4	Q86SJ6
DYNLL2	Q96FJ2	EPHB4	P54760	F3	P13726
FGF4	P08620	FOXL2	P58012	HSPA8	P11142
IL12A	P29459	KPNA2	P52292	KRT83	P78385, Q14533
LIN7B	Q9HAP6	LRRC41	Q15345	LUM	P51884
MAP3K5	Q99683	MASTL	Q96GX5	MMP10	P09238
MRPL9	Q9BYD2	MYF5	P13349	NAGK	Q9UJ70
NTF3	P20783	NUP133	Q8WUM0	ODC1	P11926
OSR1	Q8TAX0	PCBP1	Q15365	PKMYT1	Q99640
PNPLA6	Q8IY17	PTH	P01270	PTPN4	P29074
RAB30	Q15771	RACGAP1	Q9H0H5	RAD23A	P54725
RB1CC1	Q8TDY2	RBBP8	Q99708	RHOC	P08134, P61586
RNF6	Q9Y252	RPA2	P15927	S100A10	P31151
SENP3	Q9H4L4	SORL1	Q92673	SPHK2	Q9NRA0
STK11	Q15831	STK11IP	Q8N1F8	TCP1	P17987
TGFB1	P01137	TGM1	P22735	THBS3	P49746
TOPORS	Q9NS56	TRIM3	P53365	TRIP11	Q15643
TSG101	Q99816	UBC	P0CG48	VIM	P08670
WNT10A	Q9GZT5	ZFPM2	Q8WW38	ZFYVE9	O95405-1
ZNF217	O75362	ZNF791	Q3KP31, Q8IZC7		

Input	Ensembl Id	Input	Ensembl Id	Input	Ensembl Id
ACTA2	ENSG00000107796	CCL20	ENSG00000115009	CEBPE	ENSG00000092067
F3	ENSG00000117525	FOXL2	ENSG00000183770	HSPA8	ENSG00000109971
IL12A	ENSG00000168811	KPNA2	ENST00000330459.7	MMP10	ENSG00000166670
OSR1	ENSG00000143867	PTPN4	ENSG00000088179	RBBP8	ENSG00000101773
STK11	ENSG00000118046	TCP1	ENSG00000120438	TGFB1	ENSG00000105329
TRIM3	ENSG00000110171	VIM	ENSG00000026025		

Interactors (69)

Input	UniProt Id	Interacts with	Input	UniProt Id	Interacts with
ALX1	Q15699	Q92993	ANKHD1	Q8IWZ3-3	Q92993
APCDD1	Q8J025	O75197	ATP1A1	P05023	P0DTC4
BSCL2	Q96G97-4	P54252	BTG3	Q14201	Q01094
BTK	Q06187	Q06187	CA11	Q9NS71	Q53GS7
CCL20	P78556	P13501, P48061	CD69	Q07108	P21453
CEBPE	Q15744	P35638	CHAF1A	Q13111	P83916

Input	UniProt Id	Interacts with	Input	UniProt Id	Interacts with
CPNE1	Q99829	P13569	DAXX	Q9UER7	P46100
DNM2	P50570	Q99962	DYNLL2	Q96FJ2	Q96LC9
DYRK1B	Q9Y463	Q09472	ELK3	P41970	O00712, Q12857
EPHB4	P54760	P01588	F3	P13726	P08709
HSPA8	P11142	P03485	IL12A	P29459	P29460
JAKMIP1	Q96N16	P51451	KPNA2	P52292	O96017
KRT83	P78385	Q12837	LIN7B	Q9HAP6	Q8N3R9
LUM	P51884	P50281	MAP3K5	Q99683	P25445
MRPL9	Q9BYD2	P21673	MYF5	P13349	Q13526
NAGK	Q9UJ70	Q99081	NUP133	Q8WUM0	Q8WYP5
ODC1	P11926	Q92993	PCBP1	Q15365	Q13153
PHF14	O94880	P16104	PKMYT1	Q99640	P06493
PTH	Q86Y79	Q9NRD5	PTPN4	P29074	Q9BY67
RACGAP1	Q9H0H5	P35221	RAD23A	P54725	Q01831
RASD1	Q9Y272	P61978	RB1CC1	Q8TDY2	O75385
RBBP8	I6L8A6, Q99708-2, Q99708	Q08999, P06400	RHOC	P08134	P61586
RNF6	A0A0S2Z4G9	Q9Y5V3	RPA2	P15927	P23025
RSRC2	Q7L4I2-2	Q08379	S100A10	P60903	P46092
SCAMP2	O15127	P13569	SENP3	Q9H4L4	Q8IZL8
SFMBT1	Q9UHJ3	P62805	SORL1	Q92673	Q15669
SPHK2	Q9NRA0	Q96CV9	STK11	Q15831	P26927
TCP1	P17987	P52333	TGFB1	P07200, P01137	P37173
TGFB1I1	O43294	Q14289	TGM1	P22735	P13284
THBS3	P49746	P27797	TMEM165	Q9HC07	P13569
TMEM60	Q9H2L4	Q9UBD6	TRIM3	O75382	P49674
TSG101	Q99816	Q8WUM4	UBC	P0CG48	Q9Y253
VIM	P08670	O60437	ZFPM2	Q8WW38	P14136
ZFYVE9	O95405	P84022, Q15796	ZNF217	O75362	Q13547
ZNF791	Q3KP31	O76024			

7. Identifiers not found

These 17 identifiers were not found neither mapped to any entity in Reactome.

CLUL1	COX8C	From	ITFG1	MATN2	MEX3D	MPHOSPH9	NEUROG1
NRARP	PLAC1	PSRC1	SLC35F3	SMOC2	SRPX	UXS1	ZDHHC1
ZNF367							
