# Supplementary material for: Differential circulating miRNA profiles identified miR-423-5p, miR-93-5p, and miR-4532 as potential biomarkers for cholangiocarcinoma diagnosis
Source: PeerJ. 2024 Dec 10;12:e18367. doi: 10.7717/peerj.18367 (PMC11639864; doi:10.7717/peerj.18367)
Supplement: Supplemental Information 4 — The up-regulated miRNA profile of CCA against OV was predicted by reactome. [file peerj-12-18367-s004.pdf]

reactome

Pathway Analysis Report

This report contains the pathway analysis results for the submitted sample ''. Analysis was performed against Reactome version 87 on 15/03/2024. The web link to these results is:

https://reactome.org/PathwayBrowser/#/ANALYSIS=MjAyNDAzMTQwNjM0MTJfMTU2OTY%3D

Please keep in mind that analysis results are temporarily stored on our server. The storage period depends on usage of the service but is at least 7 days. As a result, please note that this URL is only valid for a limited time period and it might have expired.

Table of Contents

1. Introduction
2. Properties
3. Genome-wide overview
4. Most significant pathways
5. Pathways details
6. Identifiers found
7. Identifiers not found

1. Introduction

Reactome is a curated database of pathways and reactions in human biology. Reactions can be considered as pathway 'steps'. Reactome defines a 'reaction' as any event in biology that changes the state of a biological molecule. Binding, activation, translocation, degradation and classical biochemical events involving a catalyst are all reactions. Information in the database is authored by expert biologists, entered and maintained by Reactome's team of curators and editorial staff. Reactome content frequently cross-references other resources e.g. NCBI, Ensembl, UniProt, KEGG (Gene and Compound), ChEBI, PubMed and GO. Orthologous reactions inferred from annotation for Homo sapiens are available for 14 non-human species including mouse, rat, chicken, puffer fish, worm, fly and yeast. Pathways are represented by simple diagrams following an SBGN-like format.

Reactome's annotated data describe reactions possible if all annotated proteins and small molecules were present and active simultaneously in a cell. By overlaying an experimental dataset on these annotations, a user can perform a pathway over-representation analysis. By overlaying quantitative expression data or time series, a user can visualize the extent of change in affected pathways and its progression. A binomial test is used to calculate the probability shown for each result, and the p-values are corrected for the multiple testing (Benjamini–Hochberg procedure) that arises from evaluating the submitted list of identifiers against every pathway.

To learn more about our Pathway Analysis, please have a look at our relevant publications:

Fabregat A, Sidiropoulos K, Garapati P, Gillespie M, Hausmann K, Haw R, … D'Eustachio P (2016). The reactome pathway knowledgebase. Nucleic Acids Research, 44(D1), D481–D487. https://doi.org/10.1093/nar/gkv1351.

Fabregat A, Sidiropoulos K, Viteri G, Forner O, Marin-Garcia P, Arnau V, … Hermjakob H (2017). Reactome pathway analysis: a high-performance in-memory approach. BMC Bioinformatics, 18.

2. Properties

- This is an **overrepresentation** analysis: A statistical (hypergeometric distribution) test that determines whether certain Reactome pathways are over-represented (enriched) in the submitted data. It answers the question 'Does my list contain more proteins for pathway X than would be expected by chance?' This test produces a probability score, which is corrected for false discovery rate using the Benjamani-Hochberg method.
- 65 out of 83 identifiers in the sample were found in Reactome, where 1091 pathways were hit by at least one of them.
- All non-human identifiers have been converted to their human equivalent.
- IntAct interactors were included to increase the analysis background. This greatly increases the size of Reactome pathways, which maximises the chances of matching your submitted identifiers to the expanded pathway, but will include interactors that have not undergone manual curation by Reactome and may include interactors that have no biological significance, or unexplained relevance.
- This report is filtered to show only results for species 'Homo sapiens' and resource 'all resources'.
- The unique ID for this analysis (token) is MjAyNDAzMTQwNjM0MTJfMTU2OTY%3D. This ID is valid for at least 7 days in Reactome's server. Use it to access Reactome services with your data.

3. Genome-wide overview

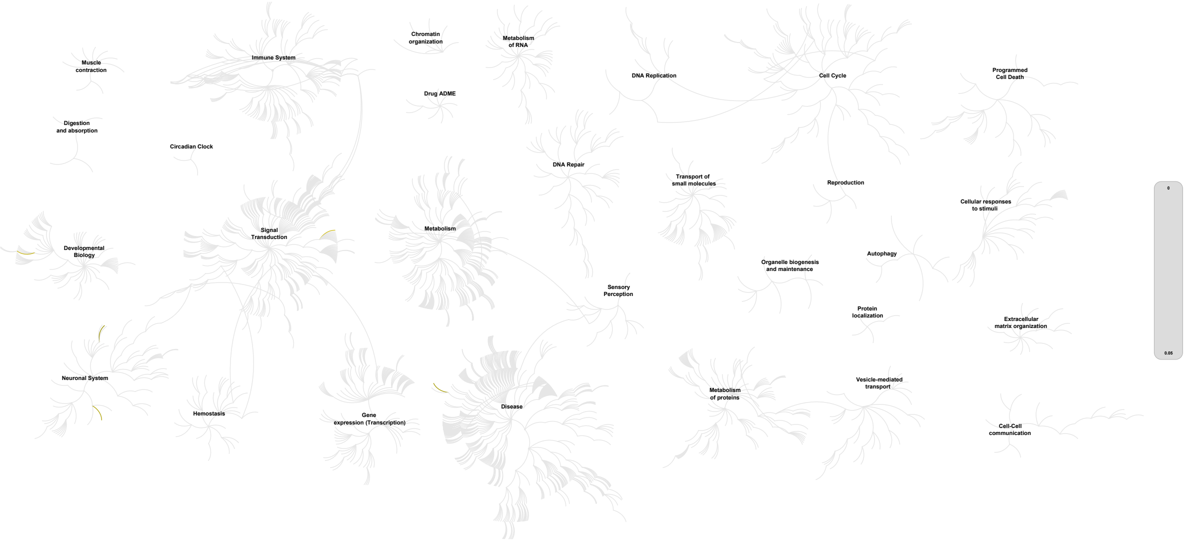

reactome

This figure shows a genome-wide overview of the results of your pathway analysis. Reactome pathways are arranged in a hierarchy. The center of each of the circular "bursts" is the root of one top-level pathway, for example "DNA Repair". Each step away from the center represents the next level lower in the pathway hierarchy. The color code denotes over-representation of that pathway in your input dataset. Light grey signifies pathways which are not significantly over-represented.

4. Most significant pathways

The following table shows the 25 most relevant pathways sorted by p-value.

Pathway name	Entities				Reactions	
	found	ratio	p-value	FDR*	found	ratio
RHO GTPases Activate Formins	5 / 203	0.009	0.019	0.754	10 / 27	0.002
Sema4D in semaphorin signaling	2 / 31	0.001	0.023	0.754	5 / 13	8.83e-04
Receptor-type tyrosine-protein phosphatases	2 / 37	0.002	0.032	0.754	2 / 6	4.08e-04
Defective Inhibition of DNA Recombination at Telomere Due to DAXX Mutations	1 / 5	2.18e-04	0.037	0.754	1 / 1	6.79e-05
GABA synthesis	1 / 6	2.62e-04	0.044	0.754	2 / 2	1.36e-04
G1/S-Specific Transcription	4 / 184	0.008	0.051	0.754	3 / 28	0.002
Signaling by membrane-tethered fusions of PDGFRA or PDGFRB	1 / 7	3.06e-04	0.051	0.754	2 / 2	1.36e-04
MECP2 regulates transcription of genes involved in GABA signaling	2 / 51	0.002	0.057	0.754	2 / 4	2.72e-04
PP2A-mediated dephosphorylation of key metabolic factors	1 / 9	3.93e-04	0.065	0.754	4 / 4	2.72e-04
Defective CHST3 causes SEDCJD	1 / 9	3.93e-04	0.065	0.754	1 / 1	6.79e-05
Defective CHST14 causes EDS, musculocontractural type	1 / 9	3.93e-04	0.065	0.754	1 / 1	6.79e-05
Defective CHSY1 causes TPBS	1 / 10	4.37e-04	0.072	0.754	2 / 2	1.36e-04
Synthesis of IPs in the ER lumen	1 / 10	4.37e-04	0.072	0.754	3 / 3	2.04e-04
N-glycan antennae elongation in the medial/trans-Golgi	2 / 59	0.003	0.073	0.754	2 / 14	9.51e-04
Opsins	1 / 11	4.80e-04	0.079	0.754	1 / 2	1.36e-04
RHO GTPases activate KTN1	1 / 12	5.24e-04	0.086	0.754	2 / 2	1.36e-04
NTRK2 activates RAC1	1 / 12	5.24e-04	0.086	0.754	1 / 2	1.36e-04
Regulation of signaling by NODAL	1 / 12	5.24e-04	0.086	0.754	1 / 3	2.04e-04
Inactivation of CDC42 and RAC1	1 / 12	5.24e-04	0.086	0.754	1 / 4	2.72e-04
Dermatan sulfate biosynthesis	1 / 13	5.68e-04	0.093	0.754	4 / 4	2.72e-04
Vitamin B1 (thiamin) metabolism	1 / 14	6.11e-04	0.1	0.754	1 / 5	3.40e-04
Signaling by GSK3beta mutants	1 / 15	6.55e-04	0.107	0.754	1 / 1	6.79e-05
ERKs are inactivated	1 / 15	6.55e-04	0.107	0.754	1 / 2	1.36e-04
Abasic sugar-phosphate removal via the single-nucleotide replacement pathway	1 / 15	6.55e-04	0.107	0.754	1 / 2	1.36e-04
MASTL Facilitates Mitotic Progression	1 / 15	6.55e-04	0.107	0.754	1 / 4	2.72e-04

* False Discovery Rate

5. Pathways details

For every pathway of the most significant pathways, we present its diagram, as well as a short summary, its bibliography and the list of inputs found in it.

1. RHO GTPases Activate Formins (R-HSA-5663220)

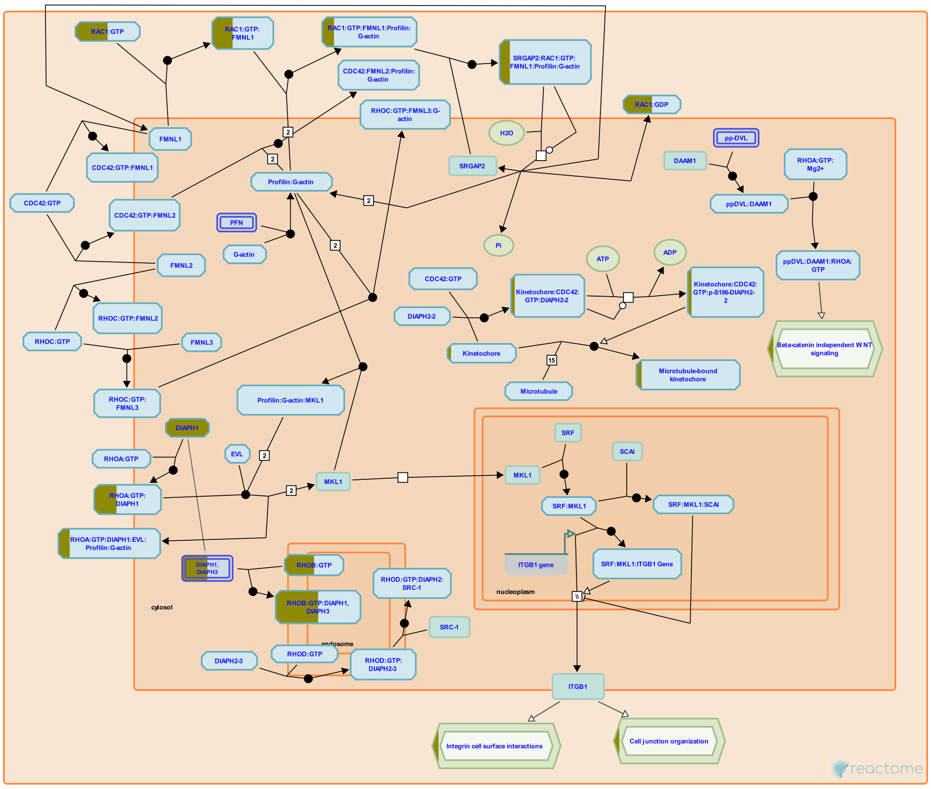

Cellular compartments: cytosol, nucleoplasm, plasma membrane, endosome membrane.

Formins are a family of proteins with 15 members in mammals, organized into 8 subfamilies. Formins are involved in the regulation of actin cytoskeleton. Many but not all formin family members are activated by RHO GTPases. Formins that serve as effectors of RHO GTPases belong to different formin subfamilies but they all share a structural similarity to Drosophila protein diaphanous and are hence named diaphanous-related formins (DRFs).

DRFs activated by RHO GTPases contain a GTPase binding domain (GBD) at their N-terminus, followed by formin homology domains 3, 1, and 2 (FH3, FH1, FH2) and a diaphanous autoregulatory domain (DAD) at the C-terminus. Most DRFs contain a dimerization domain (DD) and a coiled-coil region (CC) in between FH3 and FH1 domains (reviewed by Kuhn and Geyer 2014). RHO GTPase-activated DRFs are autoinhibited through the interaction between FH3 and DAD which is disrupted upon binding to an active RHO GTPase (Li and Higgs 2003, Lammers et al. 2005, Nezami et al. 2006). Since formins dimerize, it is not clear whether the FH3-DAD interaction is intra- or intermolecular. FH2 domain is responsible for binding to the F-actin and contributes to the formation of head-to-tail formin dimers (Xu et al. 2004). The proline-rich FH1 domain interacts with the actin-binding proteins profilins, thereby facilitating actin recruitment to formins and accelerating actin polymerization (Romero et al. 2004, Kovar et al. 2006).

Different formins are activated by different RHO GTPases in different cell contexts. FMNL1 (formin-like protein 1) is activated by binding to the RAC1:GTP and is involved in the formation of lamellipodia in macrophages (Yayoshi-Yamamoto et al. 2000) and is involved in the regulation of the Golgi complex structure (Colon-Franco et al. 2011). Activation of FMNL1 by CDC42:GTP contributes to the formation of the phagocytic cup (Seth et al. 2006). Activation of FMNL2 (formin-like protein 2) and FMNL3 (formin-like protein 3) by RHOC:GTP is involved in cancer cell motility and invasiveness (Kitzing et al. 2010, Vega et al. 2011). DIAPH1, activated by RHOA:GTP, promotes elongation of actin filaments and activation of SRF-mediated transcription which is inhibited by unpolymerized actin (Miralles et al. 2003). RHOF-mediated activation of DIAPH1 is implicated in formation of stress fibers (Fan et al. 2010). Activation of DIAPH1 and DIAPH3 by RHOB:GTP leads to actin coat formation around endosomes and regulates endosome motility and trafficking (Fernandez-Borja et al. 2005, Wallar et al. 2007). Endosome trafficking is also regulated by DIAPH2 transcription isoform 3 (DIAPH2-3) which, upon activation by RHOD:GTP, recruits SRC kinase to endosomes (Tominaga et al. 2000, Gasman et al. 2003). DIAPH2 transcription isoform 2 (DIAPH2-2) is involved in mitosis where, upon being activated by CDC42:GTP, it facilitates the capture of astral microtubules by kinetochores (Yasuda et al. 2004, Cheng et al. 2011). DIAPH2 is implicated in ovarian maintenance and premature ovarian failure (Bione et al. 1998). DAAM1, activated by RHOA:GTP, is involved in linking WNT signaling to cytoskeleton reorganization (Habas et al. 2001).

References

Carlier MF, Egile C, Didry D, Romero S, Pantaloni D & Le Clainche C (2004). Formin is a processive motor that requires profilin to accelerate actin assembly and associated ATP hydrolysis. Cell, 119, 419-29.

Banfi S, Philippe C, Arrigo G, Borsani G, Ballabio A, Manzini C, ... Zuccotti M (1998). A human homologue of the Drosophila melanogaster diaphanous gene is disrupted in a patient with premature ovarian failure: evidence for conserved function in oogenesis and implications for human sterility. Am. J. Hum. Genet., 62, 533-41.

Gomez TS, Billadeau DD & Colón-Franco JM (2011). Dynamic remodeling of the actin cytoskeleton by FMNL1? is required for structural maintenance of the Golgi complex. J. Cell. Sci., 124, 3118-26.

Nezami AG, Poy F & Eck MJ (2006). Structure of the autoinhibitory switch in formin mDia1. Structure, 14, 257-63.

Wallar BJ, Alberts AS, Deward AD & Resau JH (2007). RhoB and the mammalian Diaphanous-related formin mDia2 in endosome trafficking. Exp. Cell Res., 313, 560-71.

Edit history

Date	Action	Author
2014-10-24	Authored	Orlic-Milacic M
2014-12-26	Authored	Rivero Crespo F
2015-01-17	Created	Orlic-Milacic M
2015-02-02	Edited	Orlic-Milacic M
2023-11-16	Modified	Wright A

5 submitted entities found in this pathway, mapping to 5 Reactome entities

Input	UniProt Id	Input	UniProt Id	Input	UniProt Id
DIAPH1	O60610	MIS12	Q9H081	PPP2CB	P62714
RAC1	P63000	RHOB	P62745		

2. Sema4D in semaphorin signaling (R-HSA-400685)

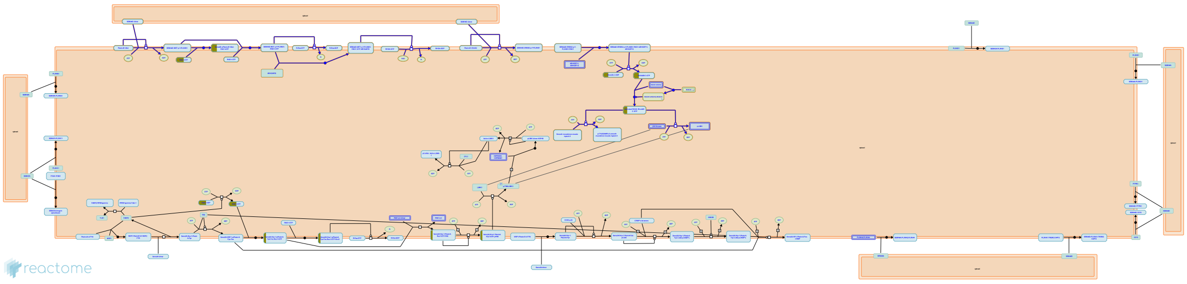

Semaphorin 4D (Sema 4D/CD100) is an axon guidance molecule with two disulfide-linked 150-kDa subunits. SEMA4D is structurally defined by a conserved 500-amino acid extracellular domain with 16 cysteines (sema domain) and also an Ig-like domain C-terminal to the sema domain. Sema4D is expressed on the cell surface as a homodimer; cysteine 679 within the sema domain is required for this dimerization.

The main receptors for Sema4D are plexin-B1 and CD72. The activation of plexins by semaphorins initiates a variety of signaling processes that involve several small GTPases of the Ras and Rho families. Sema4D-Plexin-B1 interaction appears to mediate different and sometimes opposite effects depending on the cellular context. Plexin-B1 activation inhibits integrin-mediated cell attachment and cell migration through the activation of the R-RasGAP activity inherent to plexin-B1 or through the inhibition of RhoA. However, activation of plexin-B1 by Sema4D stimulates the migration of endothelial cells by mediating the activation of RhoA.

References

Kumanogoh A & Kikutani H (2004). Biological functions and signaling of a transmembrane semaphorin, CD100/Sema4D. Cell Mol Life Sci, 61, 292-300.

Katoh H, Oinuma I & Negishi M (2005). Plexins: axon guidance and signal transduction. Cell Mol Life Sci, 62, 1363-71.

Guan KL, Aurandt J & Kruger RP (2005). Semaphorins command cells to move. Nat Rev Mol Cell Biol, 6, 789-800.

Edit history

Date	Action	Author
2009-03-23	Edited	Garapati P V
2009-03-23	Authored	Garapati P V
2009-03-24	Created	Garapati P V
2009-09-02	Reviewed	Kumanogoh A, Kikutani H
2023-11-16	Modified	Wright A

2 submitted entities found in this pathway, mapping to 2 Reactome entities

Input	UniProt Id	Input	UniProt Id
RAC1	P63000	RHOB	P62745

3. Receptor-type tyrosine-protein phosphatases (R-HSA-388844)

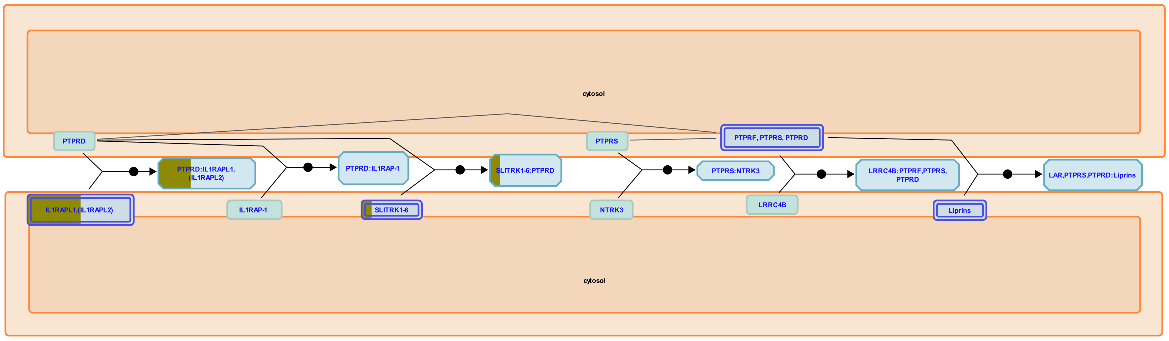

Cellular compartments: plasma membrane.

Like neurexins, Receptor-like protein tyrosine phosphatases (RPTPs) make trans-synaptic adhesion complexes with multiple postsynaptic binding partners to regulate synapse organization. The type IIa RPTPs include three members, Receptor-type tyrosine-protein phosphatase F (PTPRF) sometimes referred to as leukocyte common antigen-related (LAR), Receptor-type tyrosine-protein phosphatase sigma (PTPRS) and Receptor-type tyrosine-protein phosphatase delta (PTPRD). These proteins contain typical cell adhesion immunoglobulin-like (Ig) and fibronectin III (FNIII) domains, suggesting the involvement of RPTPs in cell-cell and cell-matrix interactions. To date, six different types of postsynaptic organizers for type-IIa RPTPs have been reported: interleukin-1 receptor accessory protein (IL1RAP, IL-1RAcP) (Yoshida et al. 2012), IL-1RAcP-like-1 (IL1RAPL1) (Yoshida et al. 2011), Neurotrophin receptor tyrosine kinase 3 (NTRK3, TrkC) (Takahashi et al. 2011), Leucine-rich repeat-containing protein 4B (LRRC4B, Netrin-G ligand-3, NGL-3) (Woo et al. 2009, Kwon et al. 2010), the Slit- and Trk-like (Slitrk) family proteins (Takahashi et al. 2012, Yim et al. 2013, Yamagata et al. 2015) and the liprins (Serra-Pagès et al. 1998, Dunah et al. 2005).

References

Craig AM & Takahashi H (2013). Protein tyrosine phosphatases PTPÎ´, PTPÏƒ, and LAR: presynaptic hubs for synapse organization. Trends Neurosci., 36, 522-34.

Edit history

Date	Action	Author
2008-12-16	Authored	Garapati P V
2008-12-16	Created	Garapati P V
2017-02-02	Edited	Jupe S
2017-02-03	Reviewed	Ko J
2023-11-16	Modified	Wright A

2 submitted entities found in this pathway, mapping to 2 Reactome entities

Input	UniProt Id	Input	UniProt Id
IL1RAPL1	Q9NZN1	SLITRK3	O94933

4. Defective Inhibition of DNA Recombination at Telomere Due to DAXX Mutations (R-HSA-9670613)

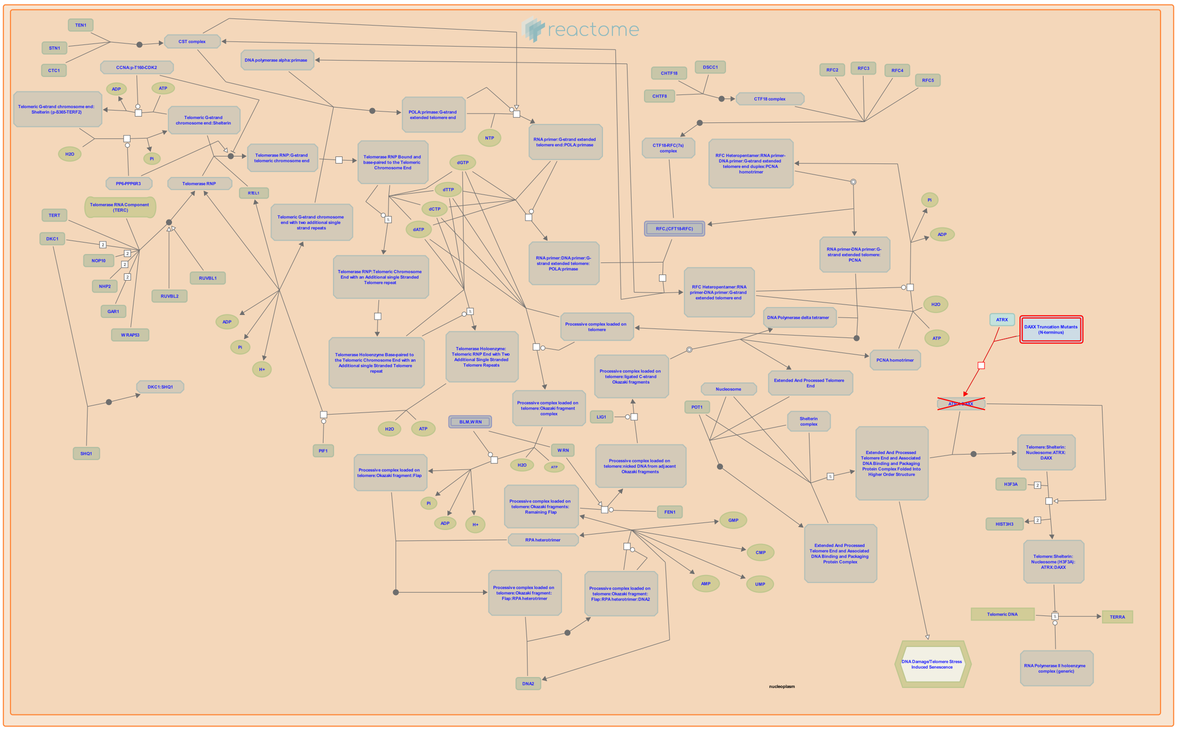

Cellular compartments: nucleoplasm.

Diseases: cancer.

A small portion of tumors that are positive for alternative lengthening of telomeres (ALT) markers and negative for mutations in the ATRX gene harbor loss-of-function mutations in the DAXX gene, which encodes the ATRX binding partner DAXX. For review, please refer to Gocha et al. 2013, and Pickett and Reddel 2015.

References

Groden J, Gocha AR & Harris J (2013). Alternative mechanisms of telomere lengthening: permissive mutations, DNA repair proteins and tumorigenic progression. Mutat. Res., 743, 142-50.

Reddel RR & Pickett HA (2015). Molecular mechanisms of activity and derepression of alternative lengthening of telomeres. Nat. Struct. Mol. Biol., 22, 875-80.

Edit history

Date	Action	Author
2019-12-11	Created	Orlic-Milacic M
2020-04-30	Authored	Orlic-Milacic M
2020-11-05	Reviewed	Meeker AK
2020-11-09	Edited	Orlic-Milacic M
2020-11-13	Reviewed	Reddel RR
2020-11-16	Edited	Orlic-Milacic M
2023-03-08	Modified	Matthews L

Interactors found in this pathway (1)

Input	UniProt Id	Interacts with	Input	UniProt Id	Interacts with
EZH2	Q15910	P46100			

5. GABA synthesis (R-HSA-888568)

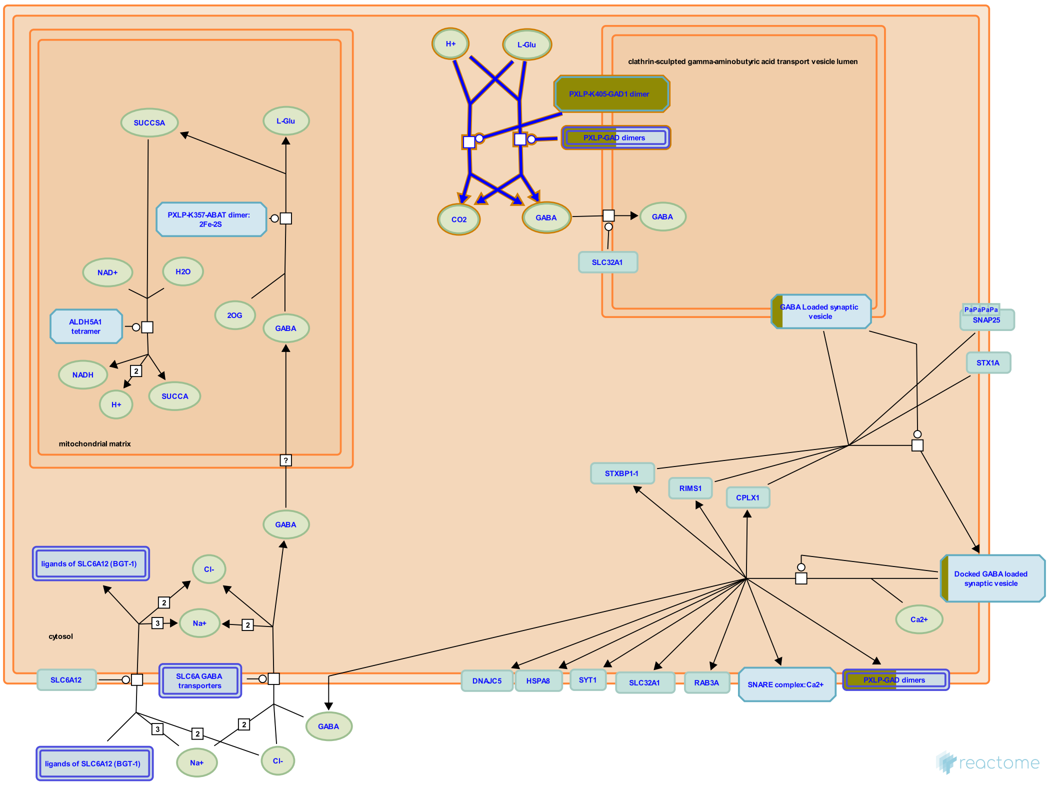

Cellular compartments: cytosol, clathrin-sculpted gamma-aminobutyric acid transport vesicle membrane.

GABA synthesized uniquely by two forms of glutamate decarboxylases, GAD65 and GAD67, that are functionally distinct and have different co-factor requirements. GAD65 is functionally linked to VGAT, the GABA transporter and selectively GABA synthesized by GAD65 is preferably loaded into the synaptic vesicles. GABA synthesized by GAD67 may be used for functions other than nuero-transmission.

References

Petroff OA (2002). GABA and glutamate in the human brain. Neuroscientist, 8, 562-73.

Barke KE & Martin DL (1998). Are GAD65 and GAD67 associated with specific pools of GABA in brain?. Perspect Dev Neurobiol, 5, 119-29.

Soghomonian JJ & Martin DL (1998). Two isoforms of glutamate decarboxylase: why?. Trends Pharmacol Sci, 19, 500-5.

Edit history

Date	Action	Author
2008-11-27	Reviewed	Restituito S
2010-06-30	Edited	Mahajan SS
2010-06-30	Authored	Mahajan SS
2010-06-30	Created	Mahajan SS
2023-11-16	Modified	Wright A

1 submitted entities found in this pathway, mapping to 1 Reactome entities

Input	UniProt Id
GAD1	Q99259

6. G1/S-Specific Transcription (R-HSA-69205)

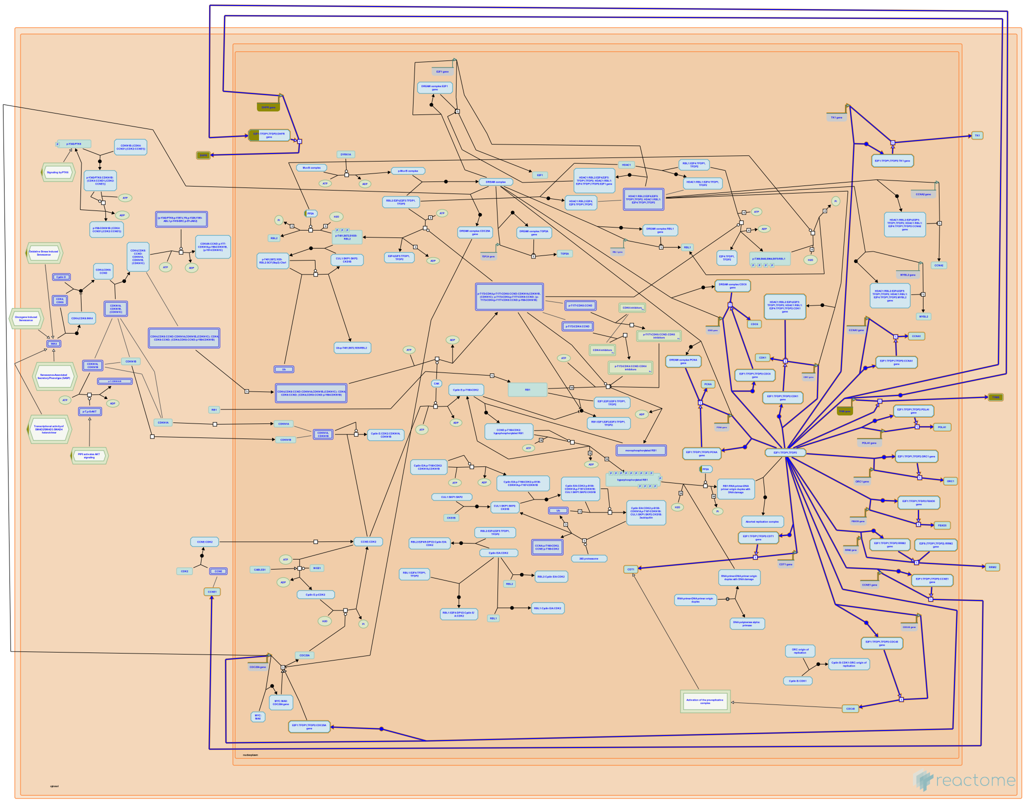

Cellular compartments: nucleoplasm.

The E2F family of transcription factors regulate the transition from the G1 to the S phase in the cell cycle. E2F activity is regulated by members of the retinoblastoma protein (pRb) family, resulting in the tight control of the expression of E2F-responsive genes. Phosphorylation of pRb by cyclin D:CDK complexes releases pRb from E2F, inducing E2F-targeted genes such as cyclin E.

E2F1 binds to E2F binding sites on the genome activating the synthesis of the target proteins. For annotation purposes, the reactions regulated by E2F1 are grouped under this pathway and information about the target genes alone are displayed for annotation purposes.

Cellular targets for activation by E2F1 include thymidylate synthase (TYMS) (DeGregori et al. 1995), Rir2 (RRM2) (DeGregori et al. 1995, Giangrande et al. 2004), Dihydrofolate reductase (DHFR) (DeGregori et al. 1995, Wells et al. 1997, Darbinian et al. 1999), Cdc2 (CDK1) (Furukawa et al. 1994, DeGregori et al. 1995, Zhu et al. 2004), Cyclin A1 (CCNA1) (DeGregori et al. 1995, Liu et al. 1998), CDC6 (DeGregori et al. 1995, Yan et al. 1998; Ohtani et al. 1998), CDT1 (Yoshida and Inoue 2004), CDC45 (Arata et al. 2000), Cyclin E (CCNE1) (Ohtani et al. 1995), Emi1 (FBXO5) (Hsu et al. 2002), and ORC1 (Ohtani et al. 1996, Ohtani et al. 1998). The activation of TK1 (Dnk1) (Dou et al. 1994, DeGregori et al. 1995, Giangrande et al. 2004) and CDC25A (DeGregori et al. 1995, Vigo et al. 1999) by E2F1 is conserved in Drosophila (Duronio and O'Farrell 1994, Reis and Edgar 2004).

RRM2 protein is involved in dNTP level regulation and activation of this enzyme results in higher levels of dNTPs in anticipation of S phase. E2F activation of RRM2 has been shown also in Drosophila by Duronio and O'Farrell (1994). E2F1 activation of CDC45 is shown in mouse cells by using human E2F1 construct (Arata et al. 2000). Cyclin E is also transcriptionally regulated by E2F1. Cyclin E protein plays important role in the transition of G1 in S phase by associating with CDK2 (Ohtani et al. 1996). E2F1-mediated activation of PCNA has been demonstrated in Drosophila (Duronio and O'Farrell 1994) and in some human cells by using recombinant adenovirus constructs (DeGregori et al. 1995). E2F1-mediated activation of the DNA polymerase alpha subunit p180 (POLA1) has been demonstrated in some human cells. It has also been demonstrated in Drosophila by Ohtani and Nevins (1994). It has been observed in Drosophila that E2F1 induced expression of Orc1 stimulates ORC1 6 complex formation and binding to the origin of replication (Asano and Wharton 1999). ORC1 6 recruit CDC6 and CDT1 that are required to recruit the MCM2 7 replication helicases. E2F1 regulation incorporates a feedback mechanism wherein Geminin (GMNN) can inhibit MCM2 7 recruitment of ORC1 6 complex by interacting with CDC6/CDT1. The activation of CDC25A and TK1 (Dnk1) by E2F1 has been inferred from similar events in Drosophila (Duronio RJ and O'Farrell 1994; Reis and Edgar 2004). E2F1 activates string (CDC25) that in turn activates the complex of Cyclin B and CDK1. A similar phenomenon has been observed in mouse NIH 3T3 cells and in Rat1 cells.

References

Ohtani K, Ikeda M, Nakamura M & Tsujimoto A (1998). Regulation of cell growth-dependent expression of mammalian CDC6 gene by the cell cycle transcription factor E2F. Oncogene, 17, 1777-85.

Wang J, Dou QP, Pardee AB, Zhao S, Helin K & Levin AH (1994). G1/S-regulated E2F-containing protein complexes bind to the mouse thymidine kinase gene promoter. J. Biol. Chem., 269, 1306-13.

Hateboer G, Vigo E, Helin K, Prosperini E, Cartwright P, Moroni MC & Muller H (1999). CDC25A phosphatase is a target of E2F and is required for efficient E2F-induced S phase. Mol Cell Biol, 19, 6379-95.

Tretiakova A, Kundu M, Gallia GL, Khalili K, Giordano A, Shcherbik N & Darbinian N (1999). Association of Pur alpha and E2F-1 suppresses transcriptional activity of E2F-1. Oncogene, 18, 6398-402 .

Kijima S, Ohtani K, Fujita M, Arata Y & Kato JY (2000). Cdk2-dependent and -independent pathways in E2F-mediated S phase induction. J Biol Chem, 275, 6337-45.

Edit history

Date	Action	Author
2003-06-05	Created	Walworth N, O'Donnell M
2023-11-06	Modified	Matthews L

2 submitted entities found in this pathway, mapping to 4 Reactome entities

Input	UniProt Id	Input	UniProt Id
DHFR	P00374	TYMS	P04818

Input	Ensembl Id	Input	Ensembl Id
DHFR	ENSG00000228716	TYMS	ENSG00000176890

7. Signaling by membrane-tethered fusions of PDGFRA or PDGFRB (R-HSA-9673768)

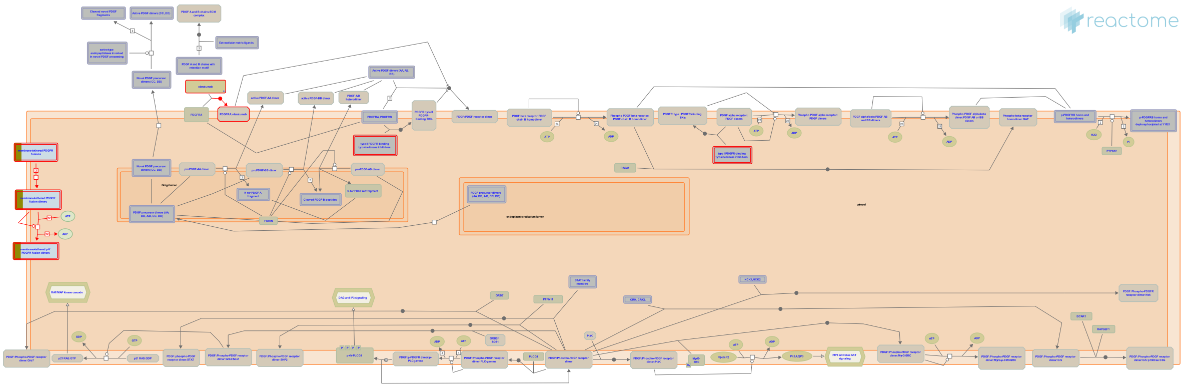

Diseases: cancer.

In addition to activating missensse and in-frame deletion mutations, PDGFRA and PDGFRB are also subject to low frequency gene fusion events arising from chromosomal rearrangements. To date there are about 35 identified PDGFRA or B fusion partners, with PDGFRB being the more common partner (reviewed in Appiah-Kubi et al, 2017). Although some of the PDGF fusions proteins are cytosolic by virtue of removal of the PDGFR transmembrane region (TMD), a number of fusions retain the TMD and are linked to the plasma membrane (Hidalgo-Curtis et al, 2010; Ozawa et al, 2010; Curtis et al, 2007; Medves et al, 2010; reviewed in Appiah-Kubi et al, 2017). The most common transmembrane fusion partner of PDGFRA and PDGFRB is ETV6 (also known as TEL1), a transcriptional repressor with known ability to homodimerize (Curtis et al, 2007; Golub et al, 1994; Andrae et al, 2008; reviewed in de Braekeleer et al, 2012; Wang et al, 2016; Appiah-Kubi et al, 2017).

References

Yao X, Chen Y, Wu M, Qian H, Wang Y, Wu Y & Appiah-Kubi K (2016). The platelet-derived growth factors (PDGFs) and their receptors (PDGFRs) are major players in oncogenesis, drug resistance, and attractive oncologic targets in cancer. Growth Factors, 34, 64-71.

De Braekeleer E, De Braekeleer M, Douet-Guilbert N, Basinko A, Le Bris MJ & Morel F (2012). ETV6 fusion genes in hematological malignancies: a review. Leuk. Res., 36, 945-61.

Betsholtz C, Andrae J & Gallini R (2008). Role of platelet-derived growth factors in physiology and medicine. Genes Dev, 22, 1276-312.

Grand FH, Clark A, Curtis CE, Reiter A, Minervini MM, Cross NC, ... Stewart J (2007). Two novel imatinib-responsive PDGFRA fusion genes in chronic eosinophilic leukaemia. Br. J. Haematol., 138, 77-81.

Golub TR, Gilliland DG, Barker GF & Lovett M (1994). Fusion of PDGF receptor beta to a novel ets-like gene, tel, in chronic myelomonocytic leukemia with t(5;12) chromosomal translocation. Cell, 77, 307-16.

Edit history

Date	Action	Author
2020-01-08	Created	Rothfels K
2020-02-06	Reviewed	Ip CKM

Date	Action	Author
2020-02-25	Edited	Rothfels K
2020-02-25	Authored	Rothfels K
2023-03-08	Modified	Matthews L

1 submitted entities found in this pathway, mapping to 1 Reactome entities

Input	UniProt Id
BIN2	Q9UBW5

8. MECP2 regulates transcription of genes involved in GABA signaling (R-HSA-9022927)

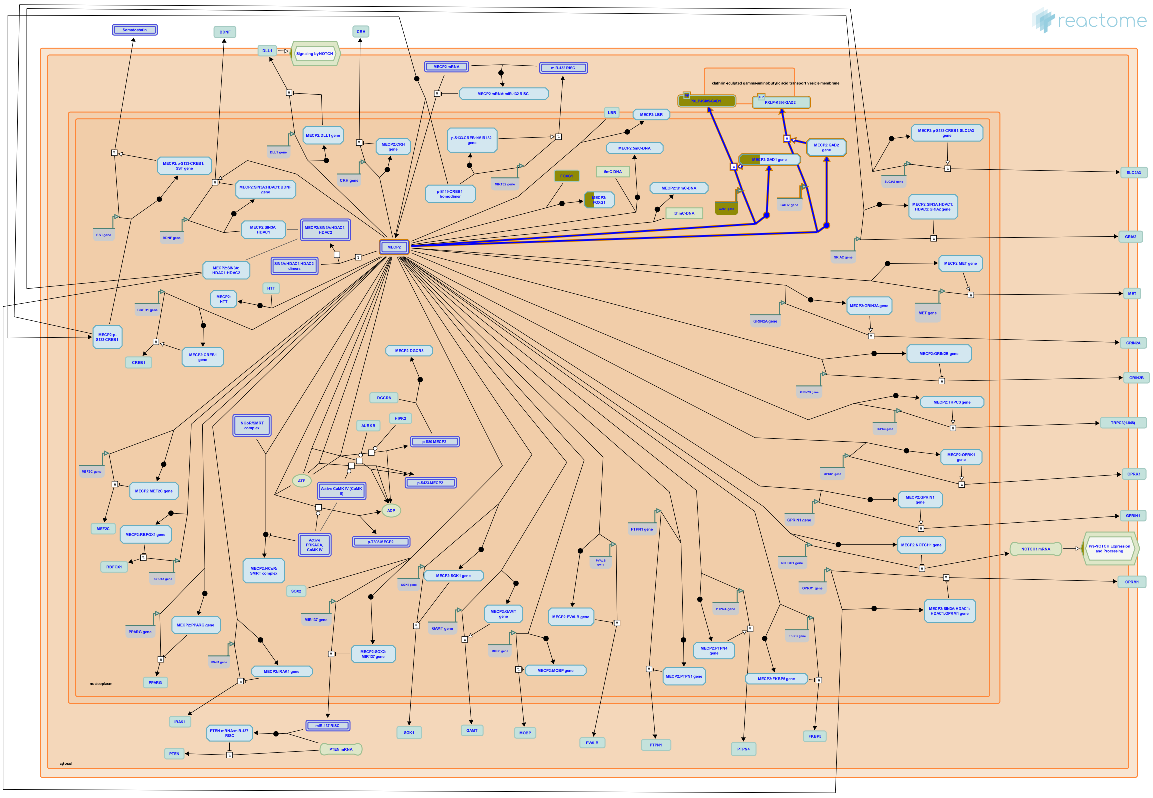

MECP2 regulates expression of several genes involved in GABA (gamma-aminobutyric acid) signaling. Transcription of GAD1 (GAD67) and GAD2 (GAD65) genes is directly positively regulated by MECP2. GAD1 and GAD2 are components of the glutamic acid decarboxylase complex involved in production of the neurotransmitter GABA. Mice lacking Mecp2 from GABA-releasing neurons have decreased GABA levels and exhibit multiple Rett syndrome features (Chao et al. 2010).

Mecp2 deletion in mouse GABAergic parvalbumin-expressing (PV) cells, cortical interneurons playing a key role in visual experience-induced ocular dominance plasticity, does not result in Rett-like phenotype, other than defects in motor coordination and motor learning. While functions of the visual cortex are preserved in mice lacking Mecp2 in GABAergic PV cells, the visual input-induced spiking responses are decreased. Mecp2 loss impairs maturation of membrane functions of cortical GABAergic PV cells. Mecp2 may be needed for PV cell-mediated cortical GABA inhibition. Mecp2-deficient cortical PV cells show reduced mRNA levels of several genes involved in GABA signaling, such as Parvalbumin, Gad2, Calretinin, Gabra1 and Gabra2, as well as reduced levels of Glu3, a glutamate receptor subunit, and Kv3.1, a potassium channel (He et al. 2014).

References

Chen XJ, Li YD, Zhang XH, Shu YS, He LJ, Liu N, ... Cheng TL (2014). Conditional deletion of Mecp2 in parvalbumin-expressing GABAergic cells results in the absence of critical period plasticity. Nat Commun, 5, 5036.

Neul JL, Chahrour M, Xue M, Samaco RC, Noebels JL, Zoghbi HY, ... Chao HT (2010). Dysfunction in GABA signalling mediates autism-like stereotypies and Rett syndrome phenotypes. Nature, 468, 263-9.

Edit history

Date	Action	Author
2017-09-26	Created	Orlic-Milacic M
2017-10-02	Authored	Orlic-Milacic M
2018-08-07	Reviewed	Christodoulou J, Krishnaraj R
2018-08-08	Edited	Orlic-Milacic M
2023-03-08	Modified	Matthews L

1 submitted entities found in this pathway, mapping to 2 Reactome entities

Input	UniProt Id
GAD1	Q99259

Input	Ensembl Id
GAD1	ENSG00000128683

9. PP2A-mediated dephosphorylation of key metabolic factors (R-HSA-163767)

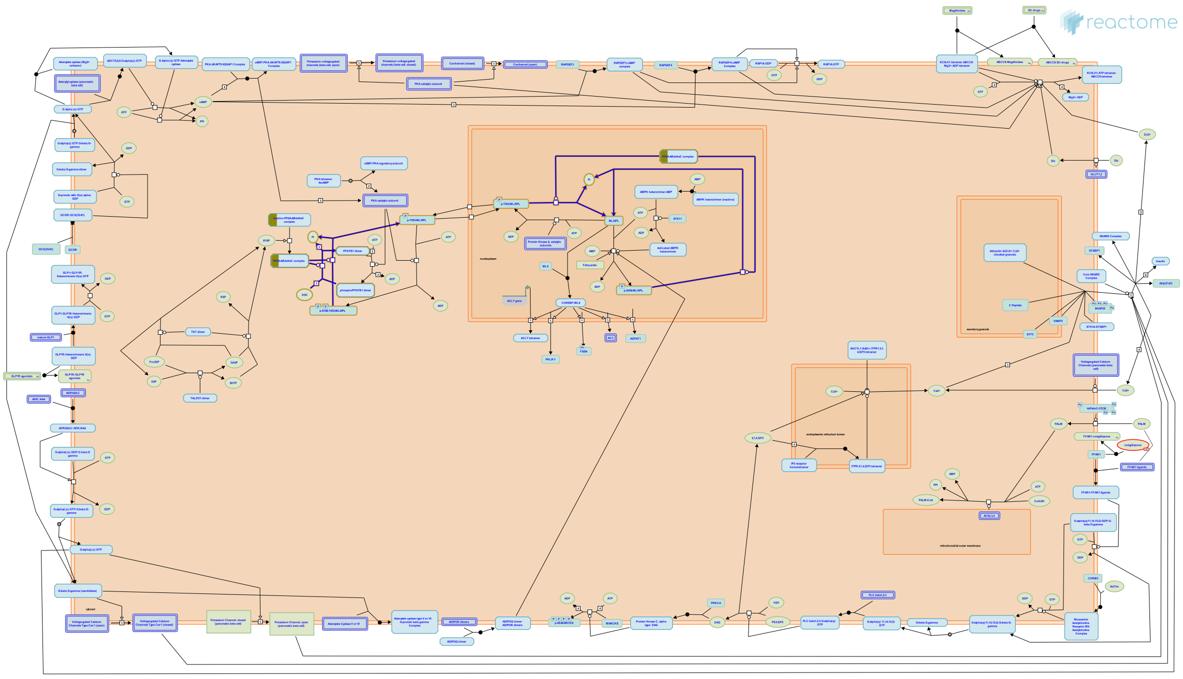

Cellular compartments: nucleoplasm, cytosol.

A member of the PP2A family of phosphatases dephosphorylates both cytosolic and nuclear forms of ChREBP (Carbohydrate Response Elemant Binding Protein). In the nucleus, dephosphorylated ChREBP complexes with MLX protein and binds to ChRE sequence elements in chromosomal DNA, activating transcription of genes involved in glycolysis and lipogenesis. The phosphatase is activated by Xylulose-5-phosphate, an intermediate of the pentose phosphate pathway (Kabashima et al. 2003). The rat enzyme has been purified to homogeneity and shown by partial amino acid sequence analysis to differ from previously described PP2A phosphatases (Nishimura and Uyeda 1995) - the human enzyme has not been characterized.

References

Veech RL (2003). A humble hexose monophosphate pathway metabolite regulates short- and long-term control of lipogenesis. Proc Natl Acad Sci U S A, 100, 5578-80.

Edit history

Date	Action	Author
2005-05-06	Created	Gopinathrao G
2005-05-13	Authored	Gopinathrao G
2023-10-12	Modified	Weiser JD

1 submitted entities found in this pathway, mapping to 1 Reactome entities

Input	UniProt Id
PPP2CB	P62714

10. Defective CHST3 causes SEDCJD (R-HSA-3595172)

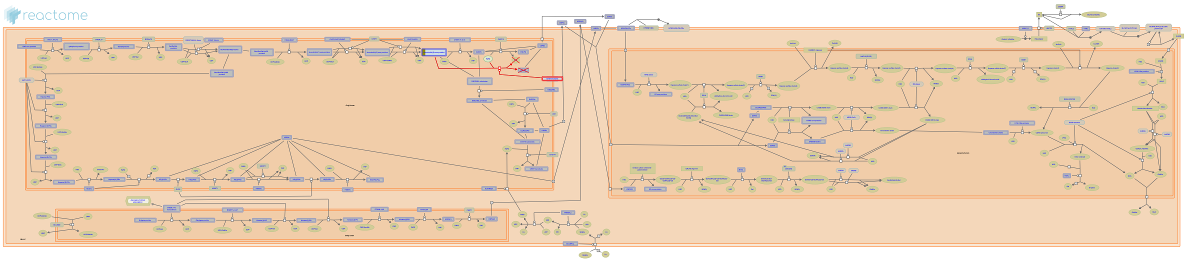

Diseases: spondyloepimetaphyseal dysplasia.

Carbohydrate sulfotransferase 3 (CHST3) transfers sulfate (SO4(2-)) to position 6 of N-acetylgalactosamine (GalNAc) residues of chondroitin-containg proteins resulting in chondroitin sulfate (CS), the predominant glycosaminoglycan present in cartilage. Defects in CHST3 result in spondyloepiphyseal dysplasia with congenital joint dislocations (SEDCJD; MIM:143095), a bone dysplasia clinically characterized by severe progressive kyphoscoliosis (abnormal curvature of the spine), arthritic changes with joint dislocations and short stature in adulthood (Unger et al. 2010).

References

Chandler K, Nampoothiri S, Bonafe L, Wakeling E, Aytes A, Unger S, ... Velten T (2010). Phenotypic features of carbohydrate sulfotransferase 3 (CHST3) deficiency in 24 patients: congenital dislocations and vertebral changes as principal diagnostic features. Am. J. Med. Genet. A, 152, 2543-9 .

Edit history

Date	Action	Author
2013-05-21	Edited	Jassal B
2013-05-21	Authored	Jassal B
2013-05-21	Created	Jassal B
2014-07-09	Reviewed	Spillmann D
2023-10-12	Modified	Weiser JD

1 submitted entities found in this pathway, mapping to 1 Reactome entities

Input	UniProt Id
VCAN	P13611

11. Defective CHST14 causes EDS, musculocontractural type (R-HSA-3595174)

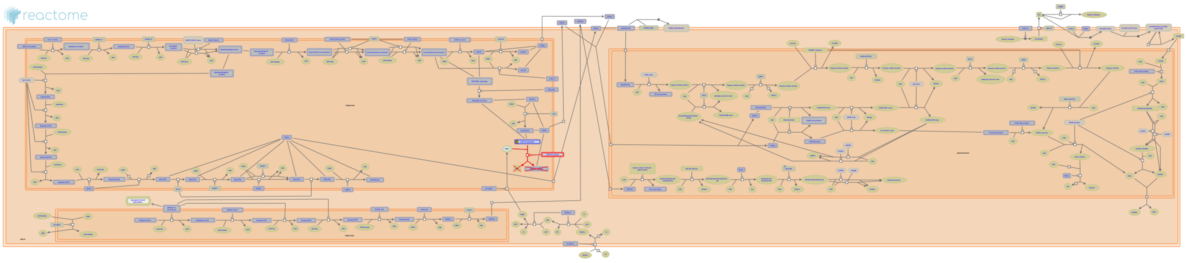

Diseases: Ehlers-Danlos syndrome.

Carbohydrate sulfotransferase 14 (CHST14 also known as D4ST-1) mediates the transfer of sulfate to position 4 of further N-acetylgalactosamine (GalNAc) residues of dermatan sulfate (DS). Defects in CHST14 cause Ehlers-Danlos syndrome, musculocontractural type (MIM:601776). The Ehlers-Danlos syndromes (EDS) are a group of connective tissue disorders that share common features such as skin hyperextensibility, articular hypermobility and tissue fragility (Beighton et al. 1998). The musculocontractural form of EDS (MIM:601776) include distinctive characteristics such as craniofacial dysmorphism, congenital contractures of fingers and thumbs, clubfeet, severe kyphoscoliosis and muscular hypotonia (Malfait et al. 2010).

References

Syx D, De Paepe A, Van Laer L, Nampoothiri S, Symoens S, Malfait F, ... Hermanns-Lê T (2010). Musculocontractural Ehlers-Danlos Syndrome (former EDS type VIB) and adducted thumb club-foot syndrome (ATCS) represent a single clinical entity caused by mutations in the dermatan-4-sulfotransferase 1 encoding CHST14 gene. Hum. Mutat., 31, 1233-9.

Tsipouras P, Beighton P, Wenstrup RJ, De Paepe A & Steinmann B (1998). Ehlers-Danlos syndromes: revised nosology, Villefranche, 1997. Ehlers-Danlos National Foundation (USA) and Ehlers-Danlos Support Group (UK). Am. J. Med. Genet., 77, 31-7.

Edit history

Date	Action	Author
2013-05-21	Edited	Jassal B
2013-05-21	Authored	Jassal B
2013-05-21	Created	Jassal B
2014-07-09	Reviewed	Spillmann D
2023-10-12	Modified	Weiser JD

1 submitted entities found in this pathway, mapping to 1 Reactome entities

Input	UniProt Id
VCAN	P13611

12. Defective CHSY1 causes TPBS (R-HSA-3595177)

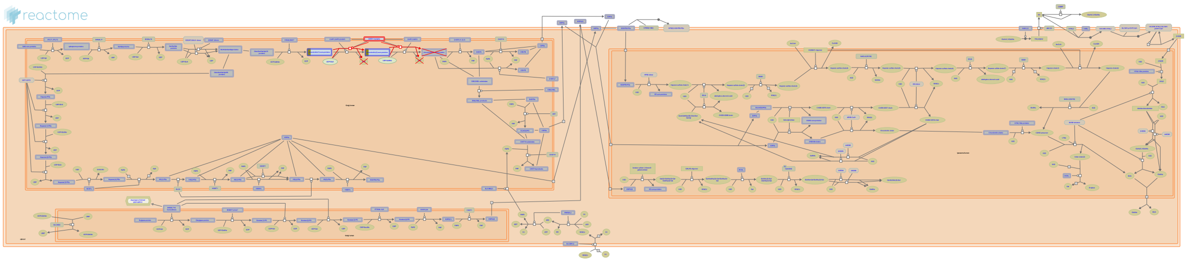

Diseases: brachydactyly.

Chondroitin sulfate synthases (CHSY) are involved in the synthesis of chondroitin sulfate, adding alternatingly glucuronate (GlcA) and N-acetylgalactosamine (GalNAc) to the growing chondroitin polymer (Mizumoto et al. 2013). Defects in CHSY1 cause temtamy preaxial brachydactyly syndrome (TPBS; MIM:605282), a syndrome characterized by multiple congenital anomalies, mental retardation, sensorineural deafness, growth retardation and bilateral symmetric digital anomalies mainly in the form of preaxial brachydactyly (literally, shortness of fingers and toes) and hyperphalangism (Temtamy et al. 1998, Race et al. 2010, Tian et al. 2010).

References

Sugahara K, Ikegawa S & Mizumoto S (2013). Human genetic disorders caused by mutations in genes encoding biosynthetic enzymes for sulfated glycosaminoglycans. J. Biol. Chem., 288, 10953-61.

Ling L, Lee H, Al-Hadidy A, Ababneh OH, Hamamy H, O'Connor B, ... Cool S (2010). Loss of CHSY1, a secreted FRINGE enzyme, causes syndromic brachydactyly in humans via increased NOTCH signaling. Am J Hum Genet, 87, 768-78.

Hall CM, Race H, Wakeling EL, Harrison MG & Quarrell OW (2010). A distinct autosomal recessive disorder of limb development with preaxial brachydactyly, phalangeal duplication, symphalangism and hyperphalangism. Clin. Dysmorphol., 19, 23-7.

Temtamy SA, Ramzy MI, Ismail SI & Meguid NA (1998). A new multiple congenital anomaly, mental retardation syndrome with preaxial brachydactyly, hyperphalangism, deafness and orodental anomalies. Clin. Dysmorphol., 7, 249-55.

Edit history

Date	Action	Author
2013-05-21	Edited	Jassal B
2013-05-21	Authored	Jassal B
2013-05-21	Created	Jassal B
2014-07-09	Reviewed	Spillmann D
2023-10-12	Modified	Weiser JD

1 submitted entities found in this pathway, mapping to 1 Reactome entities

Input	UniProt Id
VCAN	P13611

13. Synthesis of IPs in the ER lumen (R-HSA-1855231)

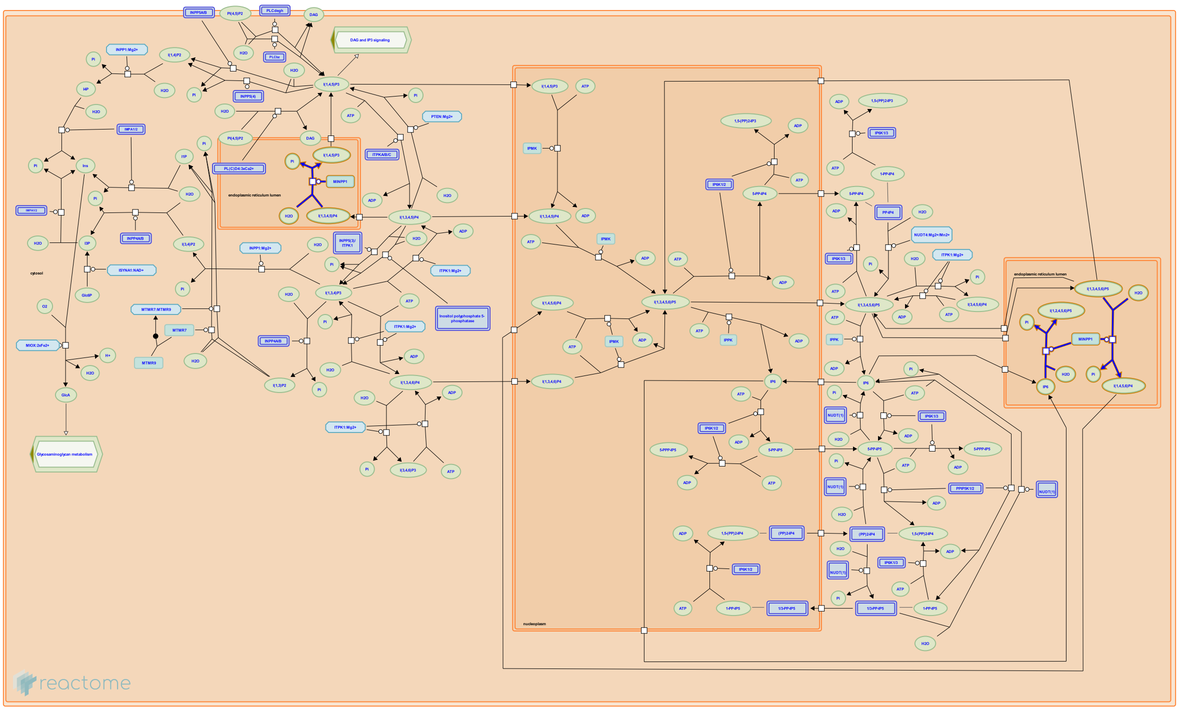

In the endoplasmic reticulum (ER) lumen, inositol phosphates IP4, IP5, and IP6 are dephosphorylated by multiple inositol polyphosphate phosphatase 1 (MINPP1) (Caffrey et al. 1999, Chi et al. 1999, Deleu et al. 2006, Nogimori et al. 1991).

References

Chi H, Reynolds PR, O'keefe RJ, Romano PR, Rosier RN, Wang J, ... Puzas JE (1999). Multiple inositol polyphosphate phosphatase: evolution as a distinct group within the histidine phosphatase family and chromosomal localization of the human and mouse genes to chromosomes 10q23 and 19. Genomics, 56, 324-36.

Hughes PJ, Hodgson ME, Putney JW Jr, Glennon MC, Shears SB & Nogimori K (1991). Purification of an inositol (1,3,4,5)-tetrakisphosphate 3-phosphatase activity from rat liver and the evaluation of its substrate specificity. J Biol Chem, 266, 16499-506.

Hidaka K, Matsuda M, Caffrey JJ, Hirata M & Shears SB (1999). The human and rat forms of multiple inositol polyphosphate phosphatase: functional homology with a histidine acid phosphatase up-regulated during endochondral ossification. FEBS Lett, 442, 99-104.

Cho J, Choi K, Sulis ML, Pesesse X, Parsons R, Deleu S & Shears SB (2006). Physiological levels of PTEN control the size of the cellular Ins(1,3,4,5,6)P(5) pool. Cell Signal, 18, 488-98.

Edit history

Date	Action	Author
2011-10-28	Edited	Williams MG
2011-10-28	Authored	Williams MG
2011-10-28	Created	Williams MG
2012-11-07	Reviewed	Wundenberg T
2023-11-16	Modified	Wright A

Interactors found in this pathway (1)

Input	UniProt Id	Interacts with	Input	UniProt Id	Interacts with
UBQLN2	Q9UHD9	Q9UNW1			

14. N-glycan antennae elongation in the medial/trans-Golgi (R-HSA-975576)

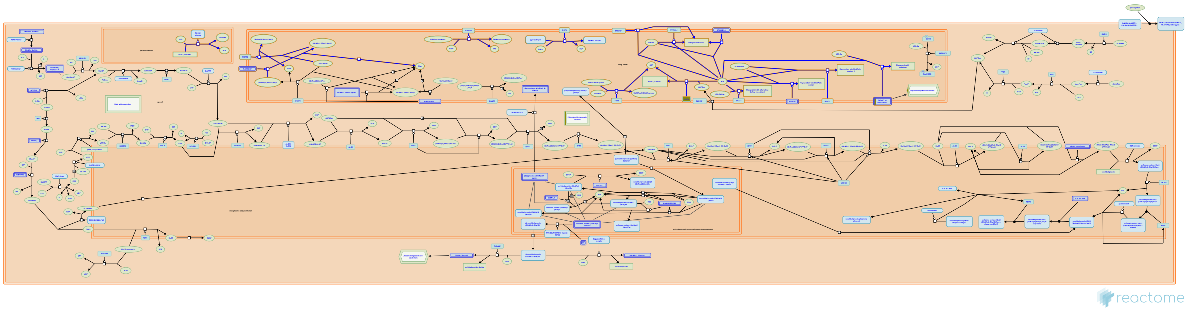

In the latter compartments of the distal Golgi the N-Glycan is further modified, leading to the wide range of N-Glycans observed in multicellular organisms. The first step of N-Glycan elongation in the Golgi is the addition of a GlcNAc residue on the alpha 1,3 branch by the enzyme MGAT1 (GlcNAc-TI), which commits the elongation pathway to Complex or Hybrid N-Glycans from Oligomannose N-Glycans. At this point, the pathway bifurcates again to generate Complex or Hybrid N-Glycans. The addition of a GlcNAc in the middle of the two arms of the N-Glycan, catalyzed by MGAT3 (GNT-III), inhibits the removal of the mannoses on the alpha1,3 branches by MAN2 and the addition of a GlcNAc by MGAT2 (GlcNAc-TII), and commits the pathway toward the synthesis of hybrid N-Glycans. Alternatively, the removal of these mannoses and the action of MGAT2 leads to the synthesis of complex N-Glycans (Kornfeld and Kornfeld 1985).

The exact structure of the network of reactions leading to Complex or Hybrid N-Glycans is still not completely described and validated experimentally. Here we will annotate only one generic reaction for each of the enzymes known to participate in this process. For a better annotation on the reactions and genes involved in the synthesis of Complex and Hybrid N-Glycans we recommend the GlycoGene Database (Ito H. et al, 2010) (http://riodb.ibase.aist.go.jp/rcmg/ggdb/textsearch.jsp) for annotations on genes, and the Consortium for Functional Genomics (http://riodb.ibase.aist.go.jp/rcmg/ggdb/textsearch.jsp) for annotation of Glycan structures and reactions. Moreover, a computationally inferred prediction on the structure of this network is available through the software GlycoVis (Hossler P. et. al. 2006).

References

Kornfeld S & Kornfeld R (1985). Assembly of asparagine-linked oligosaccharides. Annu Rev Biochem, 54, 631-64.

Hu WS, Lee MM, Hossler P & Goh LT (2006). GlycoVis: visualizing glycan distribution in the protein N-glycosylation pathway in mammalian cells. Biotechnol Bioeng, 95, 946-60.

Schachter H (2000). The joys of HexNAc. The synthesis and function of N- and O-glycan branches. Glycoconj J, 17, 465-83.

Ito H, Narimatsu H, Sato T, Kameyama A & Chiba Y (2010). In vitro and in vivo enzymatic syntheses and mass spectrometric database for N-glycans and o-glycans. Methods Enzymol, 478, 127-49.

Edit history

Date	Action	Author
2009-11-10	Authored	Dall'Olio GM
2010-09-30	Edited	Jassal B
2010-09-30	Created	Jassal B

Date	Action	Author
2010-11-18	Reviewed	Gagneux P
2023-11-16	Modified	Wright A

2 submitted entities found in this pathway, mapping to 2 Reactome entities

Input	UniProt Id	Input	UniProt Id
B4GALT2	O60909	FUT8	Q9BYC5

15. Opsins (R-HSA-419771)

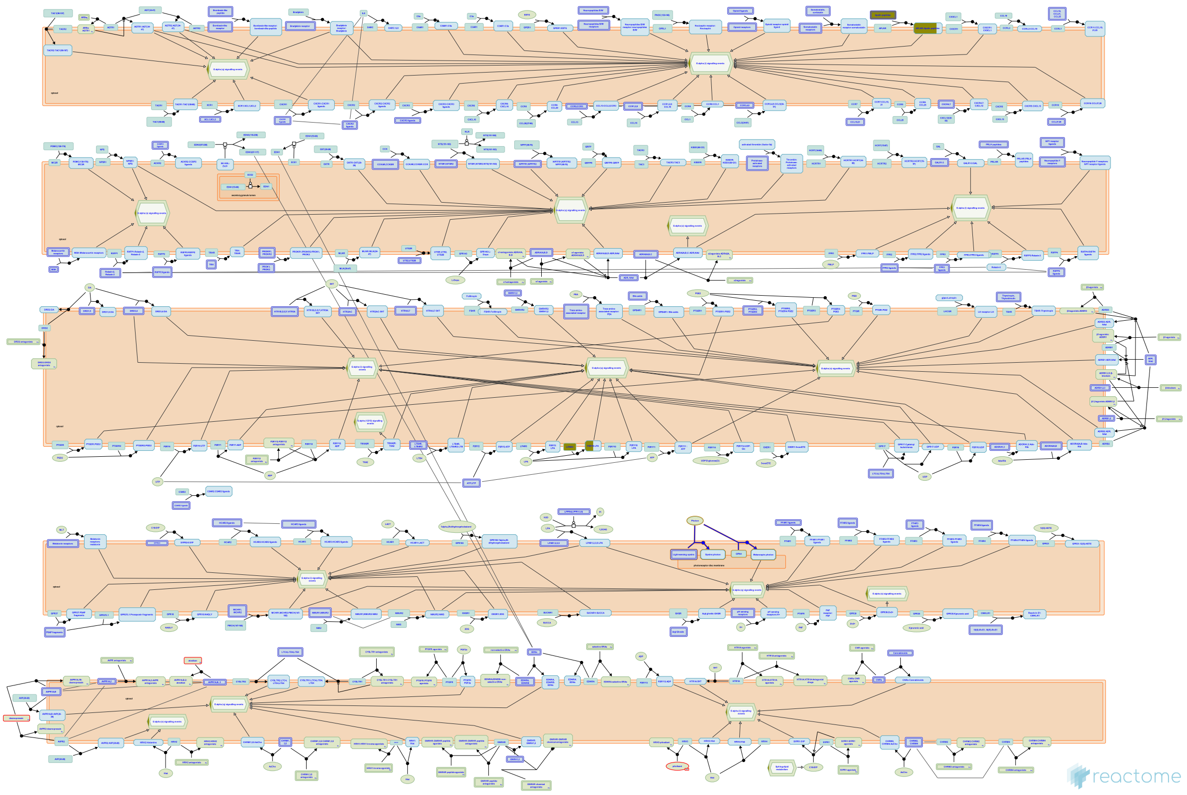

Opsins are light-sensitive, 35-55 kDa membrane-bound G protein-coupled receptors of the retinylidene protein family found in photoreceptor cells of the retina. Five classical groups of opsins are involved in vision, mediating the conversion of a photon of light into an electrochemical signal, the first step in the visual transduction cascade (Terakita A, 2005; Nickle B and Robinson PR, 2007). Another opsin found in the mammalian retina, melanopsin, is involved in circadian rhythms and pupillary reflex but not in image-forming (Hankins MW et al, 2008; Kumbalasiri T and Provencio I, 2005). Guanine nucleotide-binding proteins (G proteins) are involved as modulators or transducers in various transmembrane signaling systems. The G protein transducin, encoded by GNAT genes, is one of the transducers of a visual impulse that performs the coupling between rhodopsin and cGMP-phosphodiesterase. Defects in GNAT1 are the cause of congenital stationary night blindness autosomal dominant type 3, also known as congenital stationary night blindness Nougaret type. Congenital stationary night blindness is a non-progressive retinal disorder characterized by impaired night vision (Dryja TP et al, 1996). Defects in GNAT2 are the cause of achromatopsia type 4 (ACHM4). Achromatopsia is an autosomal recessively inherited visual disorder that is present from birth and that features the absence of color discrimination (Kohl S et al, 2002).

References

Provencio I & Kumbalasiri T (2005). Melanopsin and other novel mammalian opsins. Exp Eye Res, 81, 368-75.

Hahn LB, Reboul T, Arnaud B & Dryja TP (1996). Missense mutation in the gene encoding the alpha subunit of rod transducin in the Nougaret form of congenital stationary night blindness. Nat Genet, 13, 358-60.

Terakita A (2005). The opsins. Genome Biol, 6, 213.

Kohl S, Vadalà M, Lorenz B, Rosenberg T, Wissinger B, Jacobson SG, ... Baumann B (2002). Mutations in the cone photoreceptor G-protein alpha-subunit gene GNAT2 in patients with achromatopsia. Am J Hum Genet, 71, 422-5.

Peirson SN, Hankins MW & Foster RG (2008). Melanopsin: an exciting photopigment. Trends Neurosci, 31, 27-36.

Edit history

Date	Action	Author
2009-05-07	Edited	Jassal B
2009-05-07	Authored	Jassal B
2009-05-07	Created	Jassal B
2009-05-29	Reviewed	D'Eustachio P
2023-11-16	Modified	Wright A

Interactors found in this pathway (1)

Input	UniProt Id	Interacts with	Input	UniProt Id	Interacts with
UBQLN2	Q9UHD9	Q9UHM6			

16. RHO GTPases activate KTN1 (R-HSA-5625970)

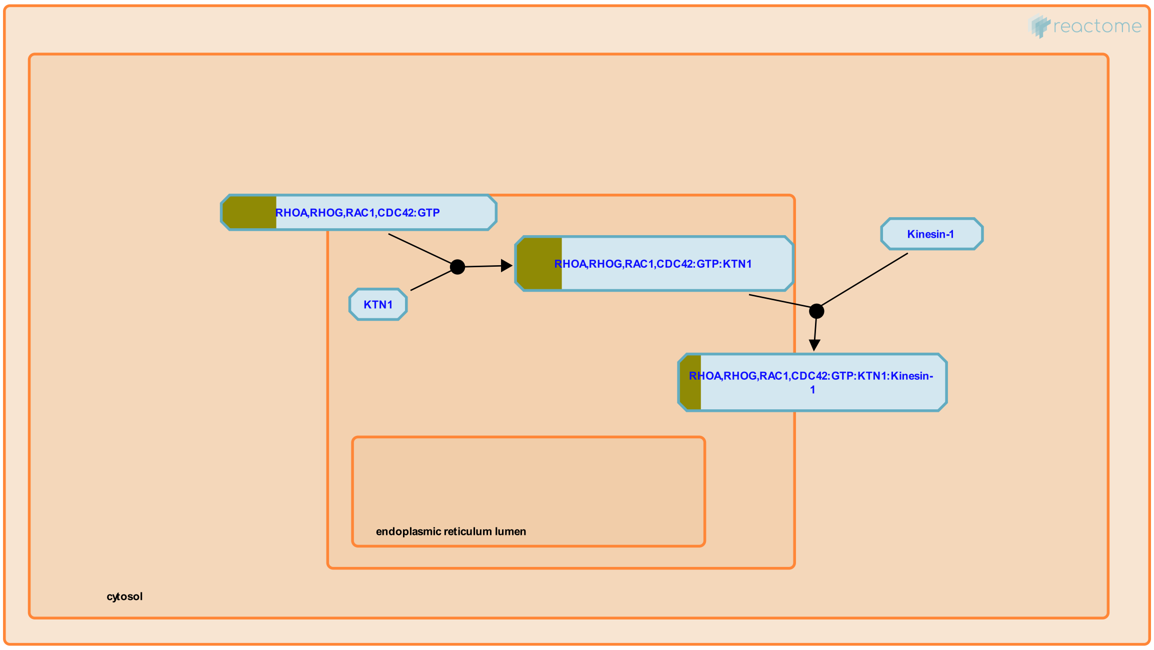

Cellular compartments: endoplasmic reticulum membrane, cytosol.

GTP-bound active forms of RHO GTPases RHOA, RHOG, RAC1 and CDC42 bind kinectin (KTN1), a protein inserted in endoplasmic reticulum membranes that interacts with the cargo-binding site of kinesin and activates its microtubule-stimulated ATPase activity required for vesicle motility (Vignal et al. 2001, Hotta et al. 1996). The effect of RHOG activity on cellular morphology, exhibited in the formation of microtubule-dependent cellular protrusions, depends both on RHOG interaction with KTN1, as well as on the kinesin activity (Vignal et al. 2001). RHOG and KTN1 also cooperate in microtubule-dependent lysosomal transport (Vignal et al. 2001). The precise mechanism of kinectin-mediated Rho GTPase signaling cascade needs further elucidation, and only the first two steps, KTN1-activated RHO GTPase binding, and KTN1-kinesin-1 binding are annotated here.

References

Blangy A, Gauthier-Rouvière C, Vignal E, Martin M & Fort P (2001). Kinectin is a key effector of RhoG microtubule-dependent cellular activity. Mol. Cell. Biol., 21, 8022-34.

Mino A, Kohno H, Hotta K, Takai Y & Tanaka K (1996). Interaction of the Rho family small G proteins with kinectin, an anchoring protein of kinesin motor. Biochem. Biophys. Res. Commun., 225, 69-74.

Edit history

Date	Action	Author
2014-10-09	Created	Orlic-Milacic M
2014-10-24	Authored	Orlic-Milacic M
2014-12-26	Authored	Rivero Crespo F
2015-02-02	Edited	Orlic-Milacic M
2023-11-16	Modified	Wright A

1 submitted entities found in this pathway, mapping to 1 Reactome entities

Input	UniProt Id
RAC1	P63000

17. NTRK2 activates RAC1 (R-HSA-9032759)

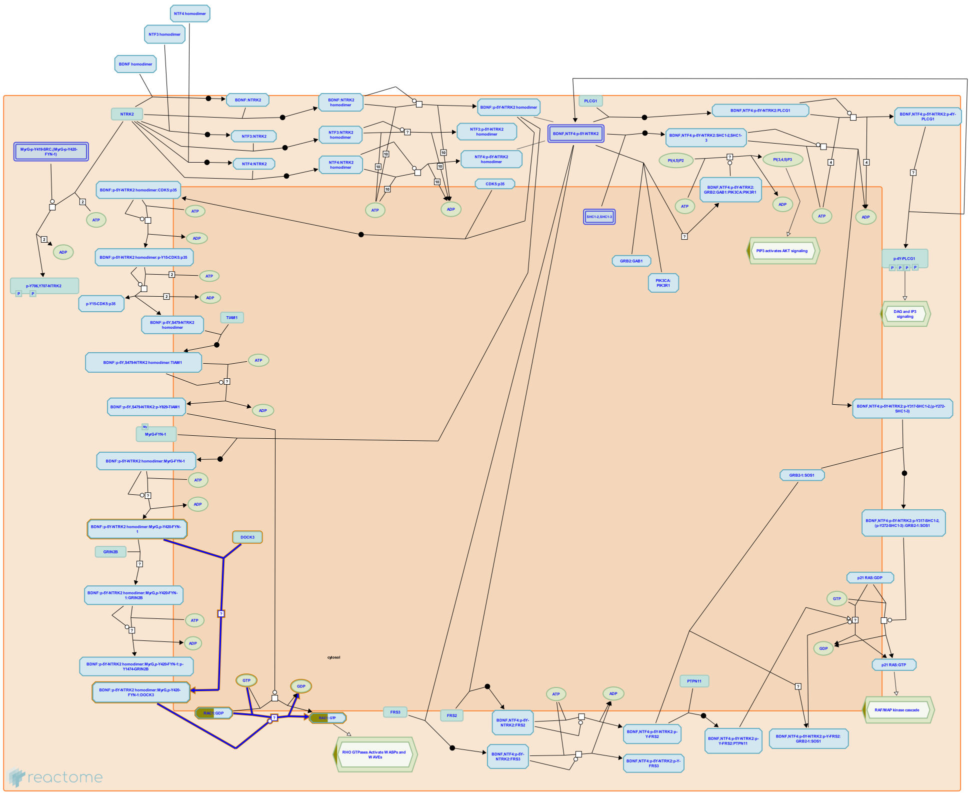

DOCK3-mediated activation of RAC1 downstream of BDNF-induced signaling by NTRK2 (TRKB) plays a role in axonal growth and regeneration. DOCK3 can be recruited to the plasma membrane to activate RAC1 by binding to NTRK-associated FYN (Namekata et al. 2010). Alternatively, DOCK3 can, upon poorly elucidated RHOG activation by the BDNF:NTRK2 complex, bind to the RHOG:GTP complex and activate RAC1 in an ELMO1-dependent manner (Namekata et al. 2012).

References

Harada T, Namekata K, Guo X, Harada C, Parada LF, Kimura H & Taya C (2010). Dock3 induces axonal outgrowth by stimulating membrane recruitment of the WAVE complex. Proc. Natl. Acad. Sci. U.S.A., 107, 7586-91.

Harada T, Watanabe H, Namekata K, Kimura A, Guo X, Harada C, ... Kittaka D (2012). Dock3 regulates BDNF-TrkB signaling for neurite outgrowth by forming a ternary complex with Elmo and RhoG. Genes Cells, 17, 688-97.

Edit history

Date	Action	Author
2017-12-18	Created	Orlic-Milacic M
2018-01-05	Authored	Orlic-Milacic M
2018-02-13	Reviewed	Antila H, Castrén E
2018-02-20	Edited	Orlic-Milacic M
2023-11-16	Modified	Wright A

1 submitted entities found in this pathway, mapping to 1 Reactome entities

Input	UniProt Id
RAC1	P63000

18. Regulation of signaling by NODAL (R-HSA-1433617)

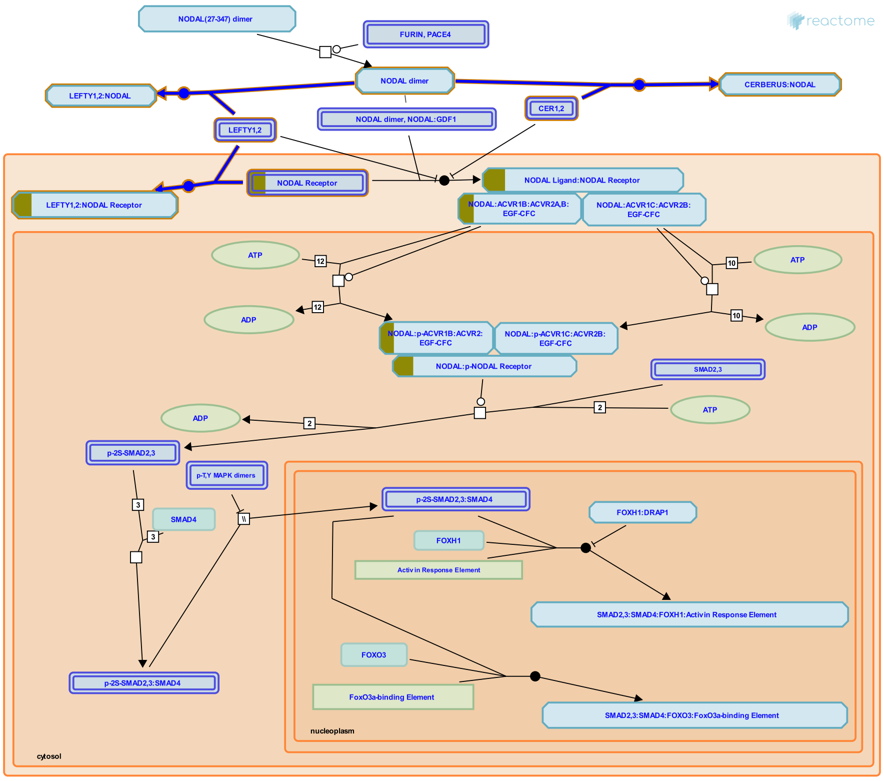

Cellular compartments: plasma membrane, extracellular region.

Mature NODAL can form heterodimers with LEFTY1, LEFTY2, or CERBERUS. The heterodimers do not activate the NODAL receptor. LEFTY1 and LEFTY2 also bind CRIPTO and CRYPTIC coreceptors and prevent them from interacting with other components of the NODAL receptor. By these mechanisms LEFTY1, LEFTY2, and CERBERUS negatively regulate NODAL signaling (reviewed in Shen 2007, Schier 2009).

References

Shen MM (2007). Nodal signaling: developmental roles and regulation. Development, 134, 1023-34.

Schier AF (2009). Nodal morphogens. Cold Spring Harb Perspect Biol, 1, a003459.

Edit history

Date	Action	Author
2011-07-11	Edited	May B
2011-07-11	Authored	May B
2011-07-12	Created	May B
2011-08-18	Reviewed	May B
2023-10-12	Modified	Weiser JD

1 submitted entities found in this pathway, mapping to 1 Reactome entities

Input	UniProt Id
ACVR2A	P27037

19. Inactivation of CDC42 and RAC1 (R-HSA-428543)

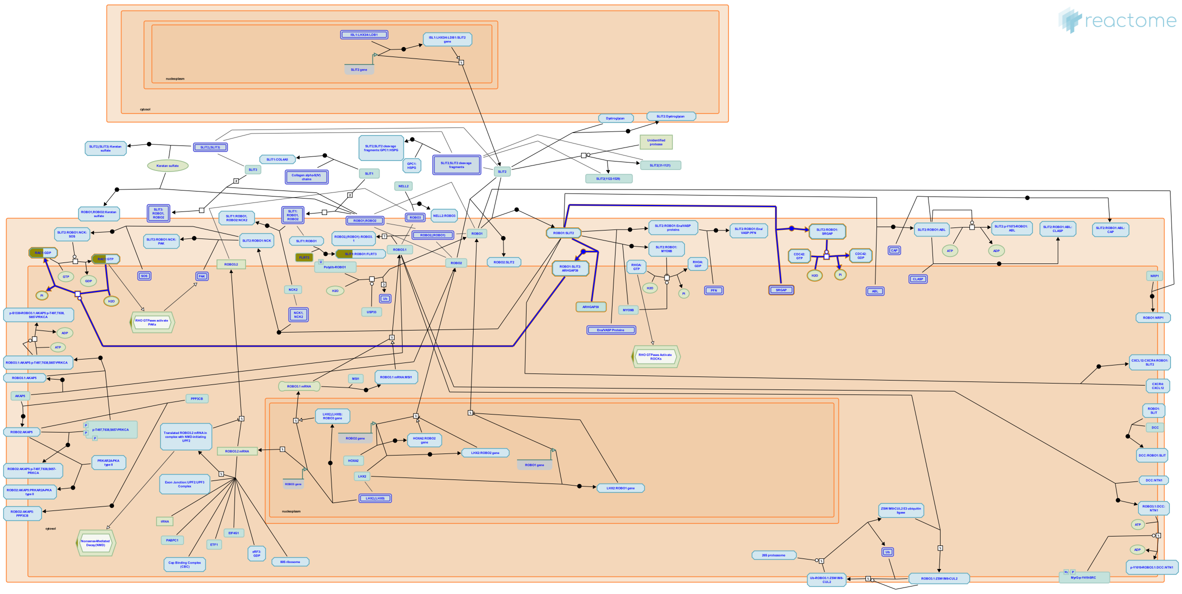

Cellular compartments: plasma membrane.

Rho family GTPases, including RAC1, RHOA, and CDC42, are ideal candidates to regulate aspects of cytoskeletal dynamics downstream of axon guidance receptors. Biochemical and genetic studies have revealed an important role for CDC42 and RAC1 in ROBO repulsion. ROBO controls the activity of Rho GTPases by interacting with a family of SLIT/ROBO-specific GAPs (SrGAPs) and Vilse/CrossGAP. SrGAPs inactivate CDC42 and Vilse/CrossGAP specifically inactivates RAC1.

It was recently implicated that SRGAP3 may inactivate RAC1 downstream of SLIT1-activated ROBO2, which promotes neurite outgrowth in mammalian dorsal root ganglion (DRG) neurons (Zhang et al. 2014).

References

Edit history

Date	Action	Author
2008-09-05	Edited	Garapati P V
2008-09-05	Authored	Garapati P V
2009-07-06	Created	Garapati P V
2009-08-18	Reviewed	Kidd T
2017-06-26	Edited	Orlic-Milacic M
2017-07-31	Reviewed	Jaworski A
2023-10-12	Modified	Weiser JD

1 submitted entities found in this pathway, mapping to 1 Reactome entities

Input	UniProt Id
RAC1	P63000

20. Dermatan sulfate biosynthesis (R-HSA-2022923)

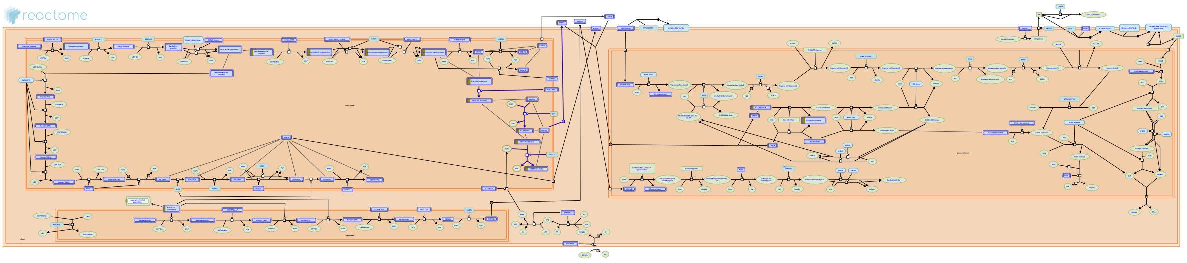

Dermatan sulfate (DS) consists of N-acetylgalactosamine (GalNAc) residues alternating in glycosidic linkages with glucuronic acid (GlcA) or iduronic acid (IdoA) residues. As with CS, GalNAc residues can be sulfated in CS chains but also the uronic acid

residues may be substituted with sulfate at the 2- and 4- positions. The steps below outline the synthesis of a simple DS chain (Silbert & Sugumaran 2002).

References

Sugumaran G & Silbert JE (2002). Biosynthesis of chondroitin/dermatan sulfate. IUBMB Life, 54, 177-86.

Edit history

Date	Action	Author
2011-12-01	Edited	Jassal B
2011-12-01	Authored	Jassal B
2011-12-01	Created	Jassal B
2012-03-28	Reviewed	D'Eustachio P
2023-11-16	Modified	Wright A

1 submitted entities found in this pathway, mapping to 1 Reactome entities

Input	UniProt Id
VCAN	P13611

21. Vitamin B1 (thiamin) metabolism (R-HSA-196819)

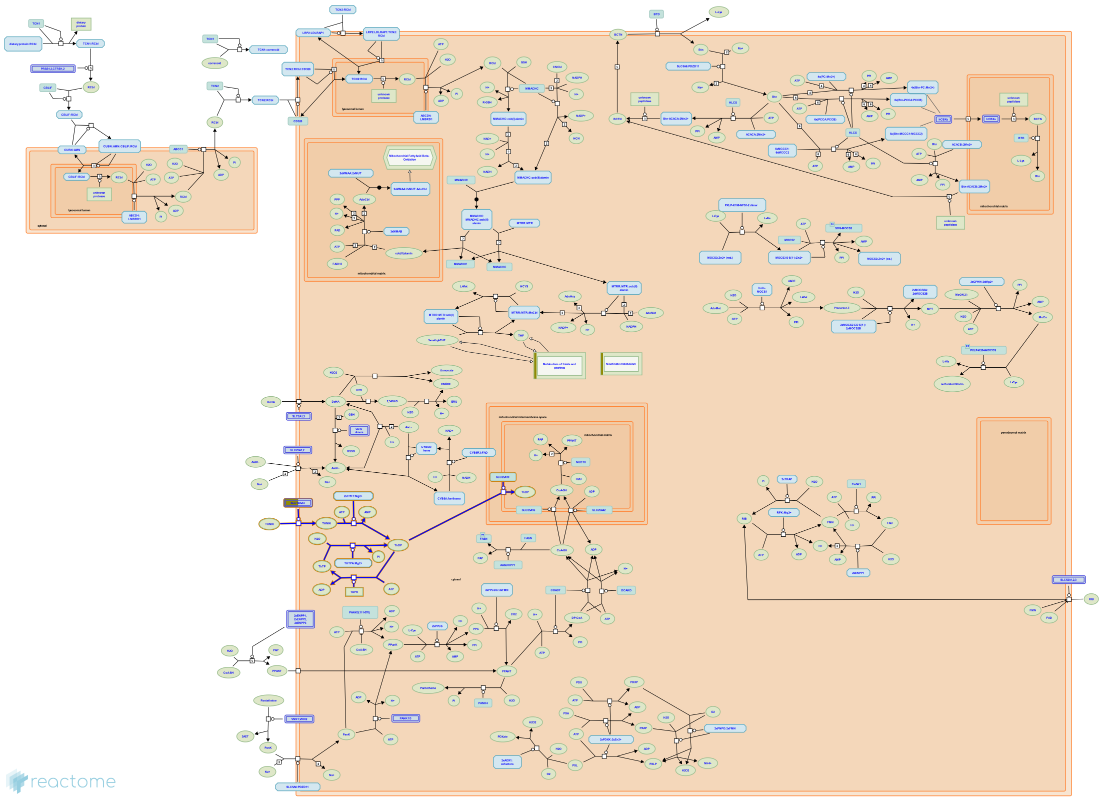

Vitamin B1 (thiamin) is found naturally in certain foodstuffs such as green peas, spinach, liver, bananas, whole grains and legumes. Human diseases associated with thiamin deficiency include beriberi, due to a thiamin-deficient diet, TMRA, due to defects in the SLC19A2 transport protein, and Wernicke-Korsakoff Syndrome, associated with thiamin deficiency in alcoholism (Haas 1988). Thiamin is water-soluble so is not stored in the body. When pyrophosphorylated, thiamin is converted into the coenzyme thiamin pyrophosphate (ThPP, codecarboxylase) which plays an essential role in oxidative decarboxylation and group transfer reactions.

References

Haas RH (1988). Thiamin and the brain. Annu Rev Nutr, 8, 483-515.

Edit history

Date	Action	Author
2007-04-24	Authored	Jassal B
2007-04-24	Created	Jassal B
2023-11-17	Modified	Wright A

1 submitted entities found in this pathway, mapping to 1 Reactome entities

Input	UniProt Id
SLC19A2	O60779

22. Signaling by GSK3beta mutants (R-HSA-5339716)

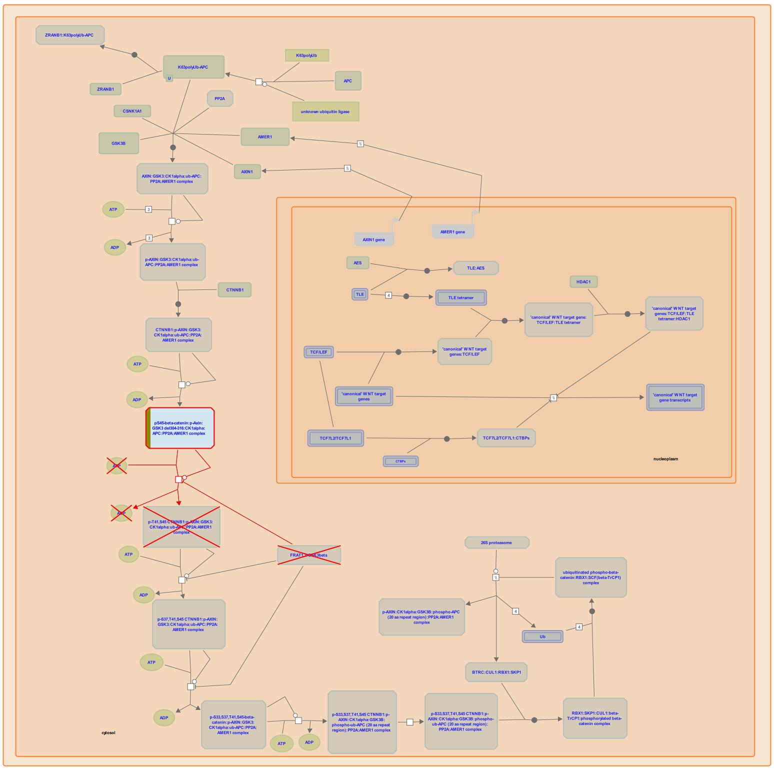

Cellular compartments: cytosol.

Diseases: chronic myeloid leukemia.

GSK3beta is subject to in-frame missplicing in CML stem cells resulting in the production of mutant protein that lacks the AXIN and FRAT binding domains. Cells containing this mutant GSK3beta show elevated levels of nuclear beta-catenin and enhanced TCF-dependent reporter activity (Jamieson et al, 2008; Abrahamsson et al, 2009).

References

Weissman IL, Jones C, Jamieson CH, Manz MG, Gotlib J, Ailles LE, ... Sawyers CL (2004). Granulocyte-macrophage progenitors as candidate leukemic stem cells in blast-crisis CML. N. Engl. J. Med., 351, 657-67.

Weissman IL, Creusot RS, Newton IG, Barroga CF, Abrahamsson AE, Negrin RS, ... Dao KH (2009). Glycogen synthase kinase 3beta missplicing contributes to leukemia stem cell generation. Proc. Natl. Acad. Sci. U.S.A., 106, 3925-9.

Edit history

Date	Action	Author
2014-02-22	Authored	Rothfels K
2014-03-08	Created	Rothfels K
2014-04-03	Edited	Matthews L
2014-05-12	Reviewed	Salahshor S
2014-05-22	Reviewed	Woodgett J

Date	Action	Author
2023-11-28	Modified	Wright A

1 submitted entities found in this pathway, mapping to 1 Reactome entities

Input	UniProt Id
PPP2CB	P62714

23. ERKs are inactivated (R-HSA-202670)

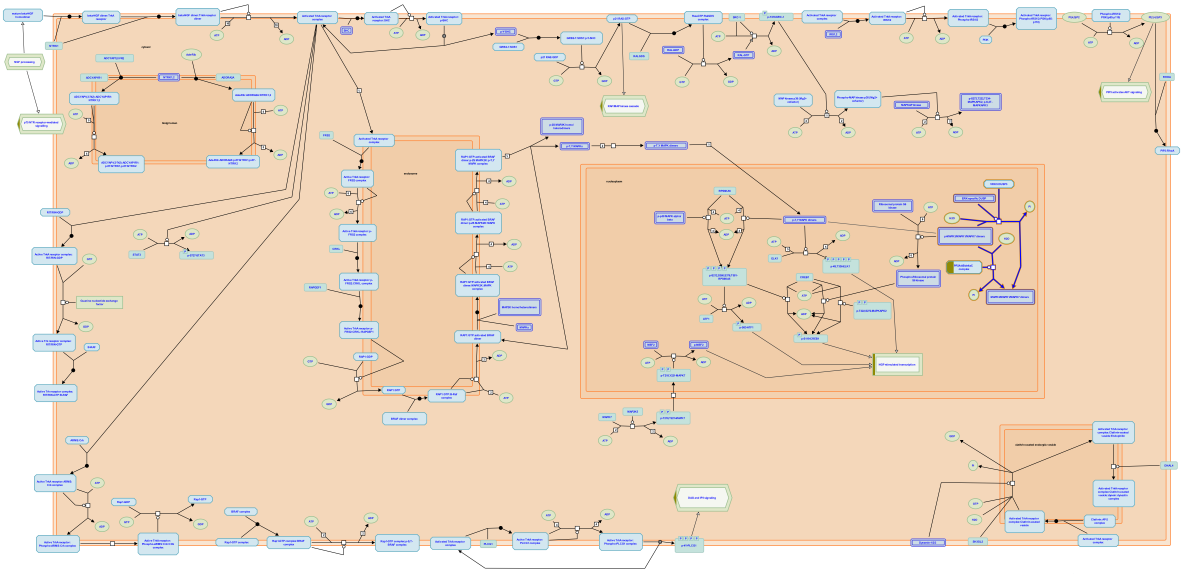

MAP Kinases are inactivated by a family of protein named MAP Kinase Phosphatases (MKPs). They act through dephosphorylation of threonine and/or tyrosine residues within the signature sequence -pTXpY- located in the activation loop of MAP kinases (pT=phosphothreonine and pY=phosphotyrosine). MKPs are divided into three major categories depending on their preference for dephosphorylating; tyrosine, serine/threonine and both the tyrosine and threonine (dual specificity phoshatases or DUSPs). The tyrosine-specific MKPs include PTP-SL, STEP and HePTP, serine/threonine-specific MKPs are PP2A and PP2C, and many DUSPs acting on MAPKs are known. Activated MAP kinases trigger activation of transcription of MKP genes. Therefore, MKPs provide a negative feedback regulatory mechanism on MAPK signaling, by inactivating MAPKs via dephosphorylation, in the cytoplasm and the nucleus. Some MKPs are more specific for ERKs, others for JNK or p38MAPK.

References

Farooq A & Zhou MM (2004). Structure and regulation of MAPK phosphatases. Cell Signal, 16, 769-79.

Siwak D, Jacob-Hirsch J, Lu Y, Amit I, Tarcic G, Segal E, ... Citri A (2007). A module of negative feedback regulators defines growth factor signaling. Nat Genet, 39, 503-12.

Edit history

Date	Action	Author
2007-11-08	Reviewed	Greene LA
2007-11-08	Created	Jassal B
2023-11-28	Modified	Wright A

1 submitted entities found in this pathway, mapping to 1 Reactome entities

Input	UniProt Id
PPP2CB	P62714

24. Abasic sugar-phosphate removal via the single-nucleotide replacement pathway (R-HSA-73930)

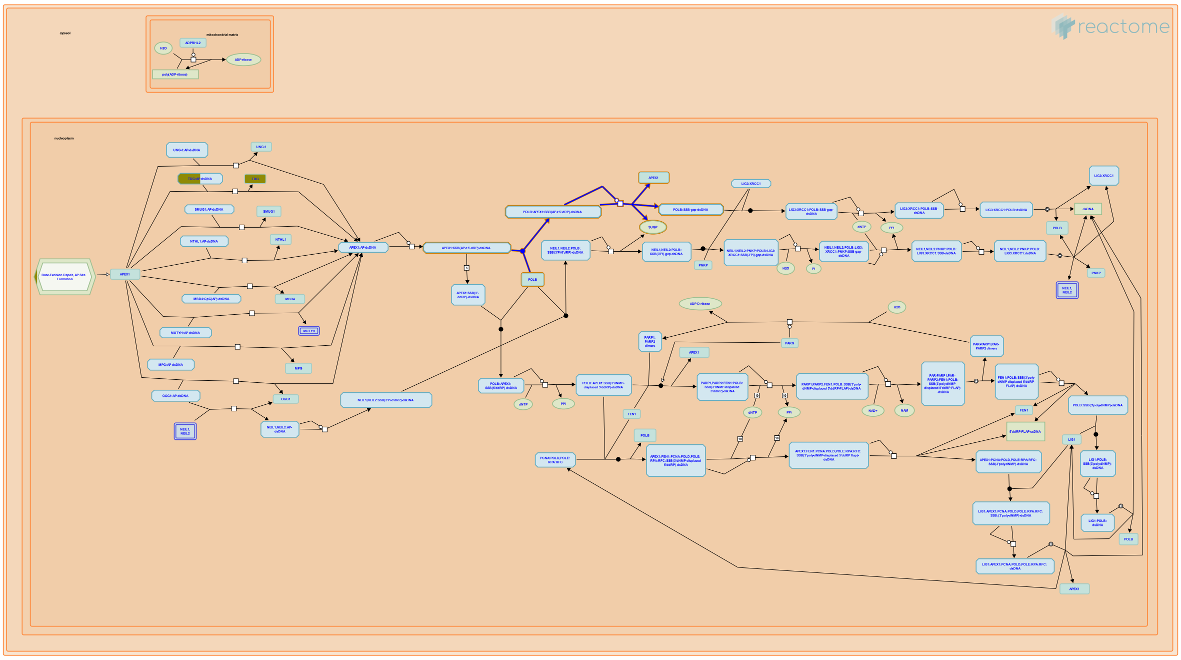

Cellular compartments: nucleoplasm.

Abasic sugar phosphate removal via the single nucleotide replacement pathway requires displacement of DNA glycosylase by APEX1, APEX1-mediated endonucleolytic cleavage at the 5' side of the base free deoxyribose residue, recruitment of POLB to the AP site and excision of the abasic sugar phosphate (5'dRP) residue at the strand break (Lindahl and Wood, 1999).

References

Lindahl T & Wood RD (1999). Quality control by DNA repair. Science, 286, 1897-905.

Edit history

Date	Action	Author
2004-01-29	Created	Matthews L
2004-02-03	Edited	Matthews L
2004-02-03	Authored	Matthews L
2014-12-04	Revised	Orlic-Milacic M
2014-12-04	Edited	Orlic-Milacic M
2014-12-22	Reviewed	Borowiec JA
2023-11-16	Modified	Wright A

Interactors found in this pathway (1)

Input	UniProt Id	Interacts with	Input	UniProt Id	Interacts with
EP300	Q09472	P27695			

25. MASTL Facilitates Mitotic Progression (R-HSA-2465910)

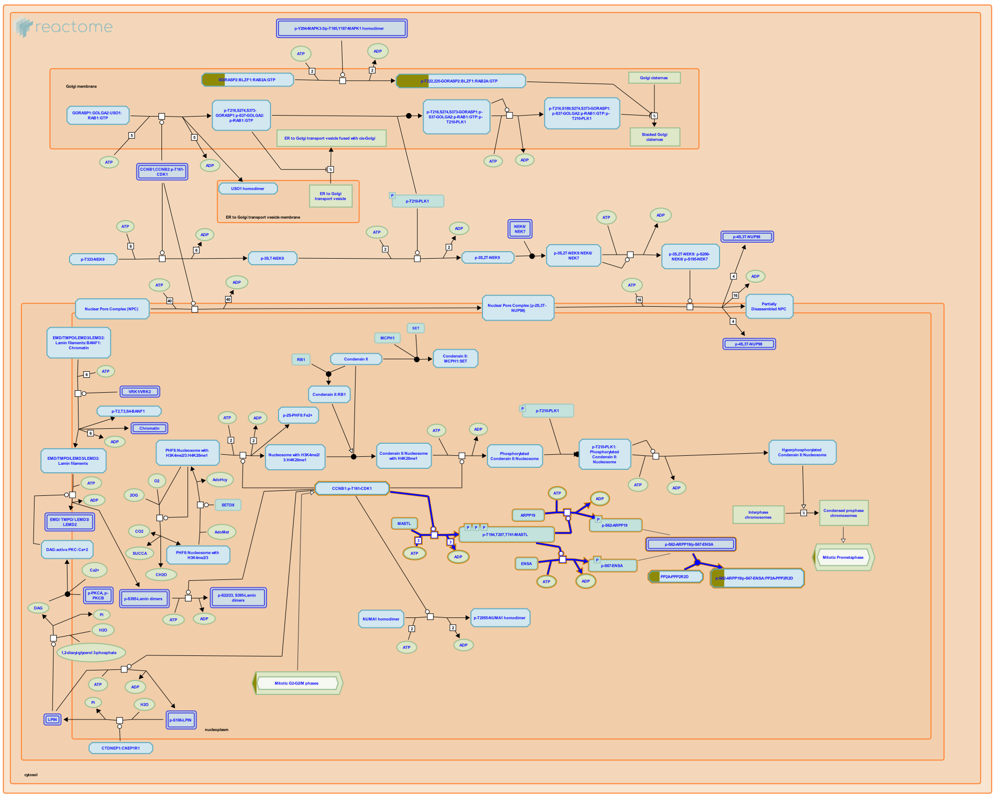

Cellular compartments: nucleoplasm.

The activity of MASTL, also known as the Greatwall kinase (GWL), is necessary for the entry and progression of mitosis. MASTL is activated by phosphorylation of several key residues during mitotic entry. Phosphorylation on the serine residue S875 (S883 in Xenopus), likely through autophosphorylation (Blake-Hodek et al. 2012) appears to be critical (Vigneron et al. 2011). Several other sites, including putative CDK1 targets T194, T207 and T741, contribute to the full activation of MASTL (Yu et al. 2006, Blake-Hodek et al. 2012). Other kinases, such as PLK1 (Vigneron et al. 2011) and other MASTL phosphorylation sites may also be functionally important (Yu et al. 2006, Blake-Hodek et al. 2012).

Activated MASTL phosphorylates ARPP19 and ENSA on serines S62 and S67, respectively, enabling them to bind to and inhibit the phosphatase activity of PP2A complexed with the regulatory subunit PPP2R2D (B55-delta). Inhibition of PP2A-PPP2R2D activity by ARPP19 or ENSA prevents dephosphorylation of CDK1 targets, hence allowing entry and maintenance of mitosis (Mochida et al. 2010, Gharbi-Ayachi et al. 2010, Burgess et al. 2010).

References

Skehel M, Mochida S, Hunt T & Maslen SL (2010). Greatwall phosphorylates an inhibitor of protein phosphatase 2A that is essential for mitosis. Science, 330, 1670-3.

Yu J, Li Z, Goldberg ML, Galas S & Zhao Y (2006). Greatwall kinase participates in the Cdc2 autoregulatory loop in Xenopus egg extracts. Mol. Cell, 22, 83-91.

Lorca T, Burgess A, Van-Dorsselaer A, Vigneron S, Strub JM, Gharbi-Ayachi A, ... Labbe JC (2010). The substrate of Greatwall kinase, Arpp19, controls mitosis by inhibiting protein phosphatase 2A. Science, 330, 1673-7.

Lorca T, Burgess A, Vigneron S, Raymond AA, Gharbi-Ayachi A, Castro A, ... Labbe JC (2011). Characterization of the mechanisms controlling Greatwall activity. Mol. Cell. Biol., 31, 2262-75.

Chen W, Castilho PV, Williams BC, Mao Y, Goldberg ML, Blake-Hodek KA, ... Zhao Y (2012). Determinants for activation of the atypical AGC kinase Greatwall during M phase entry. Mol. Cell. Biol., 32, 1337-53.

Edit history

Date	Action	Author
2012-09-04	Authored	Orlic-Milacic M
2012-09-04	Created	Orlic-Milacic M
2012-09-14	Edited	Gillespie ME
2012-09-26	Reviewed	Mochida S
2012-09-28	Reviewed	Burgess A
2023-11-17	Modified	Wright A

1 submitted entities found in this pathway, mapping to 1 Reactome entities

Input	UniProt Id
PPP2CB	P62714

6. Identifiers found

Below is a list of the input identifiers that have been found or mapped to an equivalent element in Reactome, classified by resource.

65 of the submitted entities were found, mapping to 75 Reactome entities

Input	UniProt Id	Input	UniProt Id	Input	UniProt Id
ACVR2A	P27037	ANKRD44	Q9H672	AP3B1	O00203
APBA2	Q99767	APLN	Q9ULZ1	ATP1A1	P05023
B3GALNT1	O75752	B4GALT2	O60909	BCO2	Q9BYV7
BIN2	Q9UBW5	CCNT2	O60583	CDH2	P19022
COPS7A	Q9UBW8	COQ10B	Q9H8M1	COX7A2L	O14548
CSTF3	Q12996	CTCF	P49711	DHFR	P00374
DIAPH1	O60610	EP300	Q09472	EZH2	Q15910
FLRT3	Q9NZU0	FOXG1	P55316	FUT8	Q9BYC5
GAD1	Q99259	HIBADH	P31937	IL1RAPL1	Q9NZN1
KHDRBS3	O75525	KRT83	P78385, Q14533	LPAR4	Q99677
MED14	O60244	MIS12	Q9H081	PHAX	Q9H814
PKP4	Q99569	PPP2CB	P62714	PRKCQ	Q04759
RAB2A	P61019	RAC1	P63000	RB1CC1	Q8TDY2
RBM4	Q9BWF3	RHOB	P62745	RICTOR	Q6R327
SENP3	Q9H4L4	SLC19A2	O60779	SLC39A6	Q13433
SLITRK3	O94933	SMNDC1	O75940	SOAT1	P35610
SOCS2	O14508	SPCS3	P61009	TDG	Q13569
TFG	Q92734	THSD7B	Q9C0I4	TRAIP	Q9BWF2
TYMS	P04818	UBQLN2	Q9UHD9	USP34	Q70CQ2
UTY	O14607	VCAN	P13611	VIM	P08670
WIF1	Q9Y5W5	WNK1	Q9H4A3	ZDHHC21	Q8IVQ6
ZFHX3	Q15911	ZNF131	P52739		

Input	Ensembl Id	Input	Ensembl Id	Input	Ensembl Id
DHFR	ENSG00000228716	EZH2	ENSG00000106462	GAD1	ENSG00000128683
PRKCQ	ENSG00000065675	SOCS2	ENSG00000120833	TYMS	ENSG00000176890
VIM	ENSG00000026025				

Interactors (78)

Input	UniProt Id	Interacts with	Input	UniProt Id	Interacts with
ANKHD1	Q8IWZ3-3	Q92993	ANKRD44	Q8N8A2	O00308
AP3B1	O00203	P0DTC4	APBA2	O35431	P05067-4
APLN	D3DTF8	P06396	ARFGEF1	Q9Y6D6	P19338
ATP1A1	P05023	P0DTC4	BLCAP	P62952	O95237
CA11	Q9NS71	Q53GS7	CADPS	A2RRN7	Q15436
CCNT2	O60583-1, O60583-2, O60583	P50750	CDH2	P19022	P35222
COPS7A	Q9UBW8	Q92466	CSTF3	Q12996	Q6P1J9
CTCF	P49711	Q00341	DDX50	Q9BQ39	P03372
DIAPH1	O60610	P49593	EP300	Q09472	P27695
EZH2	Q15910	P46100	FLRT3	Q9NZU0	P05067-4

Input	UniProt Id	Interacts with	Input	UniProt Id	Interacts with
FOXG1	Q60987	O43524	FUT8	Q9BYC5	P04578
GAD1	Q99259	Q13188	HIBADH	P31937	P63027
IER5	Q5VY09	Q00613	KHDRBS3	O75525	Q8WX92
KRT83	P78385	Q12837	MAP4K3	Q8IVH8	Q13094
MED14	O60244	Q15648	MIS12	Q9H081	O60566
MYO1E	Q12965	P50570	OLIG3	Q7RTU3	Q08117
OTP	Q5XKR4	O43924	PABPC4	Q13310	Q8WV24
PAWR	Q96IZ0	Q01094	PHAX	Q9H814	P52298, Q09161
PID1	Q7Z2X4	Q8N9N5	PKP4	Q99569	Q96RT1
PPP1R3D	O95685	P62136	PPP2CB	P62714	Q86XL3
PRKCQ	Q04759	Q06187	RAB2A	P61019	Q96FJ0
RAC1	P63000	P52306	RB1CC1	Q8TDY2	O75385
RBM4	Q9BWF3-1	Q99814	RICTOR	Q6R327	Q13418
SAMD4A	Q9UPU9	P31946	SENP3	Q9H4L4	Q8IZL8
SH3RF3	Q8TEJ3	Q13177	SLC39A6	Q13433-2	Q3KNW5
SLITRK3	O94933	O76024	SLMAP	Q14BN4	Q9BRV8
SOAT1	P35610	P27824	SOCS2	O14508	Q15369, Q93034
SPCS3	P61009	P42858	TDG	Q13569	Q15788
TFG	Q92734	Q8TDS5	THSD7B	Q9C0I4	Q3SXY8
TLK2	Q86UE8	Q92985	TMEM165	Q9HC07	P13569
TMEM60	Q9H2L4	Q9UBD6	TRAIP	Q9BWF2	O15160
TRIP13	Q15645	P07902	TYMS	P04818	P19320
UBQLN2	Q9UHD9	Q9UNW1	USP34	Q70CQ2	P48729
UTY	O14607	Q14686	VCAN	P13611	P14780
VIM	P08670	O60437	WDR44	Q5JSH3	P16284
WIF1	Q9Y5W5	P78560	WNK1	Q9H4A3	Q15149
YTHDF1	Q9BYJ9	Q9Y5J3	ZBTB4	Q9P1Z0	Q08379
ZDHHC21	Q8IVQ6	Q96E29	ZFHX3	Q15911-2	O14503
ZNF131	P52739-2	Q14192	ZNF536	O15090	P48431

Input	ChEBI Id	Interacts with	Input	ChEBI Id	Interacts with
RAC1	P63000	15996			

7. Identifiers not found

These 18 identifiers were not found neither mapped to any entity in Reactome.

AGAP1	ARL5A	CBLN4	EFCAB5	EHBP1	FBXO47	FRMD4B	GOLGA6B
GPR22	KBTBD2	MYPN	PCDH9	PRDM13	SALL3	SAMD13	SLC35F3
STAG3L4	TAPBPL						
